# Supplementary material for: Assessing feasibility of establishing antimicrobial stewardship programmes in two provincial-level hospitals in Vietnam: an implementation research study
Source: BMJ Open. 2021 Oct 1;11(10):e053343. doi: 10.1136/bmjopen-2021-053343 (PMC8488745; doi:10.1136/bmjopen-2021-053343)
Supplement: Supplementary data [file bmjopen-2021-053343supp001.pdf]

Supplemental Material

Manuscript title: Establishing antimicrobial stewardship programs in 2 provincial-level hospitals in Viet Nam through an implementation research approach

Supplementary Table 1. Study outcome measures and types of data collection

Outcome	Population/ sub-group	Type of data collection
Primary outcome		
Amount of targeted agent groups and total antibiotic use	Intervention and control wards	Extraction of routine data from hospital information systems for 12 months before and 12 months after the start of intervention period
Secondary outcomes		
Changes in knowledge and perceptions of doctors before and after intervention	Intervention wards and control wards	Pre and post-intervention knowledge-attitude-practice (KAP) survey among doctors and pharmacists every 3 months
Proportions of antibiotic prescriptions which:<br>- had an indication documented<br>- had review/stop date documented<br>- were used as surgical prophylaxis for greater than 24 hours<br>- were not compliant with guidelines currently endorsed at the hospital<br>- were inappropriate	Intervention wards	Retrospective medical chart review of 200 patients receiving antibiotics in 4 intervention wards (pre and post-intervention every 3 months)
Proportion of resistant isolates for priority antibiotic-organism combinations	Intervention and control wards	WHONET data – 12 months before and 12 months after intervention
Cost of antibiotic treatment and hospitalizations<br>In-hospital mortality<br>Hospital length of stay	Intervention and control departments	Extraction of routine data from hospital information systems for 12 months before and 12 months after the start of intervention period

*categorized as suboptimal (there may be a mild or non-life-threatening allergy mismatch; or antibiotic prescription is an unreasonable choice for the likely causative or cultured pathogens) or inadequate (antibiotic prescription is unlikely to treat the likely causative or cultured pathogens; or documented or presumed indication does not require any antibiotic treatment; there may be a severe or possibly life-threatening allergy mismatch, or the potential risk of toxicity due to drug interaction; for surgical prophylaxis, the duration is greater than 24 hours (except where local guidelines endorse this) (Source: National Antimicrobial Prescribing Survey (NAPS) - Australia; https://www.naps.org.au/Default.asp)

Supplementary Table 2. Assessment of criteria for clinical microbiology data reported in the study using the Microbiology Investigation Criteria for Reporting Objectively (MICRO) framework

Item	Number	Recommendation	Assessment of microbiology data reported in this manuscript
Methods			
Study design	1*	Specimen types: Describe the types of specimen included, i.e. clinical (e.g. blood cultures) or non-diagnostic surveillance (e.g. admission and other screening swabs to diagnose carriage). If specimens were obtained for diagnostic reasons, clinical syndromes should be described where possible, and specimens/isolates stratified by clinical syndrome.	We used data from microbiology laboratory on the antimicrobial susceptibility testing results (from WHONET software) of routine clinical specimens collected for diagnostic reasons from any patients admitted to the hospital. Data were not recorded on the syndromes of patients tested. Screening samples for carriage is not common practice in these hospitals, so one should assume that all samples have been taken for diagnostic purposes.
	2*	Sampling period: State the collection timeframe for specimens yielding isolates for which data is reported, e.g. from MM/YY to MM/YY to be able to identify variability between seasons.	Data reported in this manuscript were for patients admitted to the hospitals in the year 2019 (01/JAN/2019 to 31/DEC/2019).
	3*	Sampling strategy: Describe the strategy for specimen collection, e.g. asymptomatic screening, sampling of all febrile patients, sampling at clinician discretion, sampling of specific patient groups and convenience sampling (e.g. use of isolates from an existing sample repository). Specify whether sampling followed routine clinical practice or was protocol driven. Classify specimens as from community-acquired (CAI) or hospital-acquired (HAI) infections. The definition of HAI used (e.g. HAI defined by specimen collection > 48 h after hospital admission) should be provided and should use ideally an international standard (e.g. US Centers for Disease Control).	Data were from routine sampling of specimens from any patients admitted at the hospital at clinician discretion. The data didn't allow for classification in HAI vs CAI.
	4	Target organisms: Explicitly state which organisms/organism groups were included in the report. Nomenclature should follow international standards (i.e. using approved genus/species names as summarised in the International Journal of Systematic and Evolutionary Microbiology). Lists of approved bacterial names can be downloaded from Prokaryotic Nomenclature Up-to-Date (https://www.dsmz.de/bacterial-diversity/prokaryotic-nomenclature-up-	Data was collected for all organisms identified at the routine microbiology lab of each hospital. The 5 species most frequently isolated were kept in the analysis and the present report: *Escherichia coli*, *Staphylococcus aureus*, *Klebsiella pneumoniae*, *Acinetobacter baumanii*, and *Pseudomonas aeruginosa*.

Item	Number	Recommendation	Assessment of microbiology data reported in this manuscript
		to-date.html) and the List of Prokaryotic Names with Standing in Nomenclature (http://www.bacterio.net/). Organisms considered contaminants should be listed, if appropriate (e.g. coagulase negative staphylococci or *Corynebacterium* spp..	
Setting	5*	Geographical setting: Describe the geographical distribution of specimens/patients from which isolates were obtained, at least to a country level, but preferably to a sub-national level or a geoposition.	Isolates reported were from patients in the catchment area of each hospital (in Hai Phong and Dong Thap province, Viet Nam).
	6*	Clinical setting: Describe the type and level of the healthcare facilities (e.g. primary, secondary, tertiary) from which specimens were obtained. If stating a microbiology laboratory, the centres served by the laboratory should be specified.	Microbiology data were from the routine microbiology laboratory at each of the two provincial hospitals, which are general hospitals in each province in the Viet Nam public healthcare system (consisting of national, provincial and district level hospitals).
Laboratory work	7	Specimen processing: If applicable, describe specimen collection and handling, processing and sub-culture methods for all types of specimen included. For example, if reporting AST results for blood culture and cerebrospinal fluid culture isolates, the processing of these specimens by the laboratory should be briefly explained, including how specimens are sub-cultured, the media used, incubation conditions and duration. A summary of specimen processing steps (e.g. pre-processing steps, nucleic acid extraction method (if applicable), amplification platform, contamination avoidance strategy) should be provided for molecular-only workflows (e.g. to detect *Mycobacterium tuberculosis* and rifampicin resistance using the Cepheid Xpert MTB/RIF system).	Specimen collection and handling, processing and sub-culture methods followed hospital Standard Operating Procedures developed based on the guidelines issued by the Ministry of Health (MoH) in 2017, the subsequent trainings from MoH and OUCRU. Blood culture is performed with biphasic blood culture bottles (Nam Khoa Biotek, Vietnam) in Hospital 1 and with BacT-Alert automate in Hospital 2 (Biomerieux, France). Cultures are performed on homemade media (Oxoid dehydrated culture media, UK) or ready-to-use media (DEKA, Vietnam) for blood agar, chocolate agar, and Mueller-Hinton.
	8*	Target organism identification: Details of identification methodology should be reported briefly. Where identification databases were used (e.g. bioMerieux API/bioMerieux VITEK-MS/Bruker Biotyper), the version should be specified.<br>In general, all pathogens should be identified to species level. In the case of *Salmonella* species, organisms should be identified to at least the *S. Typhi*, *S. Paratyphi*, or non-typhoidal Salmonella (NTS) level. Strain subtyping methods should be reported according to STROME-ID.	Identification was based on the conventional biochemical methods in both hospitals: API galleries (Biomerieux, France) and with manual phenotypic tests in Hospital 1, and with Vitek II (Biomerieux, France; identification database version 9.02) in Hospital 2.

Item	Number	Recommendation	Assessment of microbiology data reported in this manuscript
	9*	Antimicrobial susceptibility testing: Describe the antimicrobial susceptibility testing methods used, internal quality control processes and their interpretation, with reference to a recognised international standard, e.g. CLSI, EUCAST. Where an international standard was followed, the specific edition(s) of guidelines used should be referenced. Deviations from standard methodology should be described, along with evidence of validation. Handling of any changes to interpretative criteria during the sampling period should be documented. State whether the raw AST data (zone diameters and/or minimum inhibitory concentrations) were re-categorised with updated breakpoints or left as-is.	Antimicrobial susceptibility testing methods: disc diffusion at both hospitals; Automated Vitek was also used by Hospital 2;<br>Routine AST data at the microbiology labs was entered into WHONET 5.6 by hospital technicians. Extracted files were converted to WHONET format using BacLink, a free tool included in WHONET. AST results were categorised in S/I/R according to CLSI 2020 guidelines for the present report.
	10	Additional tests performed to identify resistance mechanisms: Describe the testing methods used for adjunctive/confirmatory antimicrobial susceptibility tests, such as enzymatic/molecular assays (e.g. Xpert MTB/RIF, mecA PCR) and inducible resistance assays, with reference to a recognised international standard, where available. Where an international standard was followed, the specific edition of guidelines used should be referenced. Deviations from standard methodology should be described, along with evidence of validation.	Not applicable.
	11*	Antimicrobial resistance definitions: Define resistance for each antimicrobial class (i.e. are isolates in the 'intermediate' category included within 'susceptible' or 'resistant' or analysed as a distinct category). If using the term, define MDR (e.g. ≥ 1 agent in ≥ 3 classes tested). For each organism type, an MDR test panel must be defined, consisting of the minimum panel of individual antimicrobial agents/classes against which an isolate must be tested for that isolate to be considered tested for MDR status. Antimicrobials to which an organism is intrinsically resistant cannot be part of the test panel or contribute to MDR status.	Definitions of resistance to carbapenem and MDR were described in the main methods section of the manuscript based on the criteria proposed by Magiorakos et al [14] as follows:<br>- For *Acinetobacter baumannii* and *Acinetobacter spp.*: MDR is defined as resistant to at least 3 of the following: cephalosporin (ceftriaxone or cefepime), aminoglycosides (amikacin, gentamicin or tobramycin), ciprofloxacin and carbapenem (imipenem or meropenem); carbapenem resistance is defined as resistant to imipenem or meropenem;<br>- For *Escherichia coli, Klebsiella pneumoniae,* and *Klebsiella spp.*: MDR is defined as resistant to at least 3 of the following: carbapenem (ertapenem, imipenem or meropenem), cephalosporin (ceftriaxone or cefepime),

Item	Number	Recommendation	Assessment of microbiology data reported in this manuscript
			aminoglycosides (amikacin, gentamicin or tobramycin) and ciprofloxacin; carbapenem resistance is defined as resistant to ertapenem, imipenem, or meropenem;<br>- For *Pseudomonas aeruginosa*: MDR is defined as resistant to at least 3 of the following 4 agents: imipenem, ceftazidime, ciprofloxacin and tobramycin; carbapenem resistance is resistant to imipenem or meropenem;
Quality assurance	12*	External quality assurance: State whether the microbiology laboratory participates in an external quality control programme and, if so, provide scheme details. Examples include the UK National External Quality Assurance Scheme (www.ukneqasmicro.org.uk) and the American College of Pathologists External Quality Assurance/Proficiency Testing Program (https://www.cap.org/)	Hospital 1: participated in two external quality control programs: UK NEQAS and Ho Chi Minh City quality assurance program<br>Hospital 2: participated in two external quality control programs: UK NEQAS and Hanoi Medical University – Viet Nam
	13	Accreditation: State whether the laboratory is accredited through a national or international body (e.g. the International Standards Organisation, ISO) and specify which assays are covered in the accreditation.	No ISO certification. Hospital 2 has participated in the Strengthening Laboratory Management Toward Accreditation (SLMTA) program.
Bias	14*	Duplicate and sequential isolates: The strategy for accounting for duplicate and sequential isolates from the same patient should be clearly detailed. Duplicate isolates are multiple isolates of the same phenotypic organism (i.e. same species and same resistance profile) from the same patient on the same date cultured either from the same clinical specimen, or from two separate clinical specimens, such as blood and CSF. Sequential isolates are isolates of the same phenotypic organism from the same patient at different dates, such as blood cultures taken on different dates. Various strategies for the handling of duplicate and sequential isolates exist, and the strategy used should be transparent as it will bias pooled resistance results. For example, inclusion of all isolates (the 'all isolate strategy') has been shown to shift pooled resistance proportions toward greater resistance, whilst inclusion of only the first isolate per patient (the 'first isolate strategy') or only the first isolate per	WHONET data were de-duplicated, one isolate representing one patient. Only the first isolate per patient, per pathogen, per reporting period (i.e. for the whole year 2019) at each hospital was included.

Item	Number	Recommendation	Assessment of microbiology data reported in this manuscript
		infection episode (the 'episode-based strategy') will shift pooled results toward susceptibility.	
Results			
	15*	Population: Describe the demographics of the population from which clinical specimens and subsequent isolates have been obtained, disaggregating age and gender data.	Population: patients admitted to each hospital in one calendar year (2019) and stratified by clinical wards. Data on the isolates were described with age and gender information in the manuscript.
	16*	Denominators: Patient and isolate denominators should be used appropriately to ensure clarity regarding the numbers included in each analysis. Of particular importance is the reporting of resistance where first- and second-line AST panels were used (i.e. not all isolates of a particular species were tested against all agents). For drugs where only a subset of isolates were tested, reporting of a percentage without the numbers of isolates tested/resistant may be highly misleading.	Denominators were presented for each percentage described in the manuscript main text. No second-line AST is used in either one of the hospitals.
	17	Site/place of acquisition: AST data from CAI and HAI should be reported and analysed separately.	Not reported from the microbiology data from lab.
	18*	Reporting resistance proportions for single agent and class resistance: Proportions of resistant isolates should be reported as number of isolates susceptible or resistant to a given antimicrobial agent/class out of actual number of isolates tested for susceptibility to that agent/class.	The manuscript focuses on specific types of resistance for each pathogen and reported the proportions of resistant isolates out of the actual number of isolates tested for susceptibility to the specific agents under each type.
	19	Reporting multidrug resistance proportions: If defined, the proportion of MDR isolates should be expressed as the number of MDR isolates out of the number of isolates tested (i.e. the number undergoing the MDR test panel specific to that organism). Single agent/class resistance should be always be reported, regardless of MDR reporting.	As criterion 18. We reported proportions of resistance to single agent or class in Supplementary Table 4.
Discussion			
	20	Discuss any reasons why bias may have been introduced into the reported	We reported the baseline AST data of the study wards in each

**Item**	**Number**	**Recommendation**	**Assessment of microbiology data reported in this manuscript**
Limitations		data, due to patient/specimen selection, isolation of organisms, or otherwise. Consider factors which may have either introduced bias into the types of organisms isolated or the antimicrobial susceptibility profiles, e.g. receipt of antimicrobials prior to specimen collection will reduce the yield of certain species and also select for more resistant organisms.	hospitals using the routine data collected for diagnostic reasons. No diagnostic stewardship program existed at baseline in either one of the 2 hospitals. Therefore, it is expected that the clinicians' testing habits have impacted the selection of samples and isolates subsequently tested for antimicrobial susceptibility. Because reimbursement of laboratory test is based on strict clinical criteria, the isolates may not represent the population of pathogens, but this bias remains constant between wards, between hospitals, and with time.

*Core 'must include' items

Supplementary Table 3. Summary of National AMS Stakeholder Meeting in March 2019

**Participating stakeholders**	Medical Services Administration (MSA) Viet Nam<br>Vietnamese Pharmaceutical Association<br>Viet Nam Centers for Disease Control and Prevention<br>Hanoi Medical University, Hanoi<br>Hanoi University of Pharmacy, Hanoi<br>Drugs Administration Viet Nam, MoH<br>MSD Pharmaceuticals<br>National Hospital for Tropical Diseases, Hanoi<br>Hospital for Tropical Diseases, Ho Chi Minh City<br>Viet Nam National Children Hospital, Hanoi<br>Cho Ray Hospital, Ho Chi Minh city<br>Uong Bi Viet Nam – Sweden Hospital, Quang Ninh<br>Can Tho General Hospital<br>Da Nang General Hospital<br>Hue General Hospital<br>Children's Hospital 1, Ho Chi Minh City<br>Hoan My Private Hospital, Viet Nam<br>World Health Organization (WHO) – Viet Nam<br>Oxford University Clinical Research Unit (OUCRU)<br>US Centers for Disease Control and Prevention – Viet Nam<br>Woolcock Viet Nam<br>PATH Viet Nam
**Key presentations and discussions**	MSA/ WHO implementation of antimicrobial stewardship in hospitals: review of current state and hospital survey;<br>AMS implementation experience of Cho Ray Hospital, Hue Central Hospital, National Hospital for Tropical Diseases, and Hoan My Hospital;<br>MSD program on antimicrobial stewardship in Viet Nam: a perspective from the pharmaceutical company;<br>OUCRU qualitative study on AMS implementation in seven hospitals in the AMR surveillance network;<br>Group discussions on the challenges faced in AMS and proposed solutions to improve the implementation;<br>WHO Coordination and Finding the Way Forward.
**Main areas for actions identified**	1. Strengthening a supportive policy environment for AMS: With the National Action Plan to combat AMR and subsequent guidelines, the legal framework has been set up and shows the strong commitment of the government in AMS implementation in Viet Nam. However, national guidelines

	clearly need revisions and updates to fit in the different contexts where AMS work is required, particularly in small and resources-limited healthcare settings.<br>2. Developing AMS training curriculum and core AMS related competencies: Training modules on AMS should be standardized and formalized into the undergraduate and Continuing Professional Development programs and available in a variety of formats including online courses. These should also be available to any healthcare professionals taking on AMS roles in hospital and other settings.<br>3. Establishing coordination and collaboration in AMS activities nationally and regionally: Coordination of activities across the country and in each region is crucial to achieve a synergy of efforts and minimize duplications and wastes of resources. Smaller hospitals can learn from and utilize resources available from larger hospitals in the same region. Hospitals with experience in implementing AMS programs and those having been identified as centers of excellence can share experience with and support other hospitals in this process.

Supplementary Table 4. Baseline proportions of resistance for common pathogens to each agent/class under evaluation

**Resistant / Tested (%)**	***E. coli***		***K. pneumoniae**** or ***Klebsiella spp.*****		***A. baumannii**** or ***Acinetobacter spp.*****		***P. aeruginosa***		***S. aureus***	
	All specimens	Blood	All specimens	Blood	All specimens	Blood	All specimens	Blood	All specimens	Blood
**Hospital 1**										
Carbapenem	45/540 (8.3)	4/32 (12.5)	44/167 (26.3)	2/10 (20.0)	66/103 (64.1)	1/4 (25.0)	36/86 (41.9)	0/2 (0.0)	-	-
Cephalosporin	360/540 (66.7)	27/32 (84.4)	95/167 (56.9)	6/10 (60.0)	87/103 (84.5)	1/4 (25.0)	34/86 (39.5)	0/2 (0.0)	-	-
Aminoglycoside	245/540 (45.4)	21/32 (65.6)	71/167 (42.5)	5/10 (50.0)	86/103 (83.5)	2/4 (50.0)	47/86 (54.7)	1/2 (50.0)	115/203 (56.7)	25/40 (62.5)
Ciprofloxacin	420/539 (77.9)	29/32 (90.6)	111/167 (66.5)	5/10 (50.0)	85/102 (83.3)	1/4 (25.0)	45/86 (52.3)	0/2 (0.0)	94/210 (44.8)	26/42 (61.9)
Ceftazidime	288/540 (53.3)	24/32 (75.0)	98/167 (58.7)	4/10 (40.0)	79/103 (76.7)	1/4 (25.0)	35/85 (41.2)	0/2 (0.0)	-	-
Cefoxitin	163/540 (30.2)	12/32 (37.5)	72/167 (43.1)	3/10 (30.0)	-	-	-	-	166/210 (79.0)	-
MDR	184/539 (34.1)	21/32 (65.6)	66/167 (39.5)	4/10 (40.0)	62/102 (60.8)	0/4 (0.0)	35/86 (40.7)	0/2 (0.0)		
**Hospital 2**										
Carbapenem	22/297 (7.4)	3/96 (3.1)	151/289 (52.2)	28/62 (45.2)	260/325 (80.0)	11/19 (57.9)	89/254 (35.0)	3/14 (21.4)	-	-
Cephalosporin	69/273 (25.3)	17/83 (20.5)	141/256 (55.1)	28/61 (45.9)	226/269 (84.0)	11/17 (64.7)	41/175 (23.4)	2/11 (18.2)	-	-
Aminoglycoside	137/297 (46.1)	39/96 (40.6)	149/288 (51.7)	32/62 (51.6)	229/326 (70.2)	11/19 (57.9)	137/254 (53.9)	6/14 (42.9)	79/178 (44.4)	14/32 (46.9)
Ciprofloxacin	197/275 (71.6)	58/88 (65.9)	188/253 (74.3)	36/60 (60.0)	252/306 (82.3)	11/19 (57.9)	124/207 (59.9)	6/12 (50.0)	71/170 (41.8)	11/31 (35.5)
Ceftazidime	121/295 (41.1)	31/95 (32.6)	182/288 (63.2)	35/62 (56.5)	273/323 (84.5)	12/19 (63.2)	64/253 (25.3)	2/14 (14.3)	-	-
Cefoxitin	17/55 (30.9)	8/23 (34.8)	29/60 (48.3)	3/5 (60.0)	-	-	-	-	79/103 (76.7)	8/17 (47.1)
MDR	55/267 (20.6)	13/83 (15.7)	127/249 (51.0)	26/59 (44.1)	166/265 (62.6)	8/17 (47.1)	66/272 (24.3)	2/25 (8.0)	-	-

* in Hospital 2; ** in Hospital 1. Cephalosporin: ceftriaxone or cefepime; Aminoglycoside: amikacin, gentamicin, or tobramycin; Carbapenem: imipenem or meropenem (for *A. baumannii*, *Acinetobacter spp.*, *P. aeruginosa*); Carbapenem: ertapenem, imipenem, or meropenem (for *E. coli, K. pneumoniae, Klebsiella spp.*).
MDR for *A. baumannii* and *Acinetobacter spp.*: resistant to at least 3 of the following: cephalosporin (ceftriaxone or cefepime), aminoglycosides (amikacin, gentamicin or tobramycin), ciprofloxacin and carbapenem (imipenem or meropenem). MDR for *E. coli, K. pneumoniae, and Klebsiella spp.*: resistant to at least 3 of the following: carbapenem (ertapenem, imipenem or meropenem), cephalosporin (ceftriaxone or cefepime), aminoglycosides (amikacin, gentamicin or tobramycin) and ciprofloxacin; MDR for *P. aeruginosa*: resistant to at least 3 of the following 4 agents: imipenem, ceftazidime, ciprofloxacin and tobramycin.
