# Supplementary material for: Assessing feasibility of establishing antimicrobial stewardship programmes in two provincial-level hospitals in Vietnam: an implementation research study
Source: BMJ Open. 2021 Oct 1;11(10):e053343. doi: 10.1136/bmjopen-2021-053343 (PMC8488745; doi:10.1136/bmjopen-2021-053343)
Supplement: Author's manuscript [file bmjopen-2021-053343.draft_revisions.pdf]

BMJ Open

BMJ Open is committed to open peer review. As part of this commitment we make the peer review history of every article we publish publicly available.

When an article is published we post the peer reviewers' comments and the authors' responses online. We also post the versions of the paper that were used during peer review. These are the versions that the peer review comments apply to.

The versions of the paper that follow are the versions that were submitted during the peer review process. They are not the versions of record or the final published versions. They should not be cited or distributed as the published version of this manuscript.

BMJ Open is an open access journal and the full, final, typeset and author-corrected version of record of the manuscript is available on our site with no access controls, subscription charges or pay-per-view fees (http://bmjopen.bmj.com).

If you have any questions on BMJ Open's open peer review process please email
info.bmjopen@bmj.com

BMJ Open

Establishing antimicrobial stewardship programs in 2 provincial-level hospitals in Viet Nam through an implementation research approach

Journal:	*BMJ Open*
Manuscript ID	bmjopen-2021-053343
Article Type:	Original research
Date Submitted by the Author:	12-May-2021
Complete List of Authors:	Vu, Huong; Oxford University Clinical Research Unit, Ha Noi Unit<br>Ta, Thi Dieu Ngan; National Hospital of Tropical Diseases<br>Huynh, Phuong Thao; Hospital for Tropical Diseases<br>Le, Minh Quang; Viet Tiep Hospital<br>Tran, Thi Thu Hanh; Viet Tiep Hospital<br>Nguyen, Thi Hien; Viet Tiep Hospital<br>Tran, Duc; Viet Tiep Hospital<br>Vu, Hai Vinh; Viet Tiep Hospital<br>Chau, Minh Duc; Dong Thap Hospital<br>Vo, Thi Hoang Dung Em; Dong Thap Hospital<br>Phan, Van Be Bay; Dong Thap Hospital<br>Nguyen, Thi Thuy Oanh; Dong Thap Hospital<br>Pham Thi Thuy, Hang; Dong Thap Hospital<br>Nguyen, Cam Tu; Oxford University Clinical Research Unit<br>Truong, Anh Quan; Oxford University Clinical Research Unit, Ha Noi Unit<br>Kesteman, Thomas; Oxford University Clinical Research Unit, Ha Noi Unit<br>Dodds Ashley, Elizabeth; Duke University<br>Anderson, D; Duke University<br>van Doorn, H. Rogier; Oxford University Clinical Research Unit; Nuffield Department of Clinical Medicine
Keywords:	Health & safety < HEALTH SERVICES ADMINISTRATION & MANAGEMENT, Health policy < HEALTH SERVICES ADMINISTRATION & MANAGEMENT, Quality in health care < HEALTH SERVICES ADMINISTRATION & MANAGEMENT, PUBLIC HEALTH

SCHOLARONE™
Manuscripts

I, the Submitting Author has the right to grant and does grant on behalf of all authors of the Work (as defined in the below author licence), an exclusive licence and/or a non-exclusive licence for contributions from authors who are: i) UK Crown employees; ii) where BMJ has agreed a CC-BY licence shall apply, and/or iii) in accordance with the terms applicable for US Federal Government officers or employees acting as part of their official duties; on a worldwide, perpetual, irrevocable, royalty-free basis to BMJ Publishing Group Ltd ("BMJ") its licensees and where the relevant Journal is co-owned by BMJ to the co-owners of the Journal, to publish the Work in this journal and any other BMJ products and to exploit all rights, as set out in our licence.

The Submitting Author accepts and understands that any supply made under these terms is made by BMJ to the Submitting Author unless you are acting as an employee on behalf of your employer or a postgraduate student of an affiliated institution which is paying any applicable article publishing charge ("APC") for Open Access articles. Where the Submitting Author wishes to make the Work available on an Open Access basis (and intends to pay the relevant APC), the terms of reuse of such Open Access shall be governed by a Creative Commons licence – details of these licences and which Creative Commons licence will apply to this Work are set out in our licence referred to above.

Other than as permitted in any relevant BMJ Author's Self Archiving Policies, I confirm this Work has not been accepted for publication elsewhere, is not being considered for publication elsewhere and does not duplicate material already published. I confirm all authors consent to publication of this Work and authorise the granting of this licence.

For peer review only

Manuscript title: Establishing antimicrobial stewardship programs in 2 provincial-level hospitals in Viet Nam through an implementation research approach

Authors: Vu Thi Lan Huong[1], Ta Thi Dieu Ngan[2], Huynh Phuong Thao[3], Le Minh Quang[4], Tran Thi Thu Hanh[4], Nguyen Thi Hien[4], Tran Duc[4], Vu Hai Vinh[4], Chau Minh Duc[5], Vo Thi Hoang Dung Em[5], Phan Van Be Bay[5], Nguyen Thi Thuy Oanh[5], Pham Thi Thuy Hang[5], Nguyen Thi Cam Tu[1], Truong Anh Quan[1], Thomas Kesteman[1], Elizabeth Dodds Ashley[6], Deverick J. Anderson[6], H Rogier van Doorn[1,7]

[1]Oxford University Clinical Research Unit, 78 Giai Phong, Hanoi, Viet Nam
[2]National Hospital for Tropical Diseases, 78 Giai Phong, Hanoi, Viet Nam
[3]Hospital for Tropical Diseases, 764 Vo Van Kiet, Ho Chi Minh City, Viet Nam
[4]Viet Tiep Hospital, 1 Nha Thuong, Cat Dai, Le Chan, Hai Phong, Viet Nam
[5]Dong Thap Hospital, 144 Mai Van Khai, My Tan, Cao Lanh City, Đong Thap
[6] Duke Antimicrobial Stewardship Outreach Network, Duke Center for Antimicrobial Stewardship and Infection Prevention, Duke University, Durham, NC 27710, United States
[7]Centre for Tropical Medicine and Global Health, Nuffield Department of Medicine, University of Oxford, Oxford, UK

Correspondence to Vu Thi Lan Huong; huongvtl@oucru.org

For peer review only

ABSTRACT

Introduction: Implementation of antimicrobial stewardship (AMS) programs should be contextualized to meet specific challenges in low- and middle-income countries (LMICs). Despite the availability of AMS guidelines for hospitals, deployment has been slow in Viet Nam. We conducted this implementation research study to investigate the feasibility and impact of AMS in two provincial hospitals.

Methods: The participatory action process included planning, implementing and evaluating phases. We conducted a national stakeholder meeting and Strengths-Weaknesses-Opportunities-Threats (SWOT) analysis to identify gaps and potential interventions, and collected baseline data for assessing needs and before-intervention indicators. Hospitals developed one-year plans utilizing existing resources from the Duke Antimicrobial Stewardship Outreach Network (DASON) with adjustments to local contexts.

Results: AMS programs in study hospitals took one year for preparation and planning due to lack of hospital-specific operating procedures and relevant staff competencies and skills. Core interventions included data use to stimulate actions, educational training to prescribers, prospective audit with feedback, and periodic evaluations to inform continuing planning and implementation. Data before implementation in 2019 showed different levels of antibiotic consumption: 951 days of therapy per 1000 days present in the control and 496 in the intervention wards in Hospital 1, and 737 and 714 in Hospital 2 respectively. Proportion of bacteria harboring multidrug resistance were 34% (of 539 isolates), 39.5% (167), 60.8% (102), and 40.7% (86) of *Escherichia coli, Klebsiella spp., Acinetobacter spp.,* and *Pseudomonas aeruginosa* in Hospital 1 and 20.6% (267), 51.0% (249), 62.6% (265), and 24.3% (272) in Hospital 2 respectively.

Conclusion: Our data confirmed the need to contextualize AMS programs in LMICs and demonstrated the usefulness of implementation research design in assessing program feasibility and impact. Specifically, developing AMS staff competencies and skills, using local data to stimulate planning and actions, and integrating program activities in routine hospital work are key to success in LMICs.

For peer review only - http://bmjopen.bmj.com/site/about/guidelines.xhtml

Key words: Antimicrobial stewardship, low- and middle-income countries, Viet Nam, implementation research, action planning

Strengths and limitations of the study

- Use a participatory and teamwork approach involving hospitals as a complex adaptive system with unique characteristics and interconnections that could be leveraged to develop locally tailored interventions.
- Employ intervention strategies that could be conducted utilizing locally available resources including clinical pharmacists and routine data use.
- Include perspectives and expertise from both policy makers to hospitals at different levels and from international and local partners.
- Cannot be generalized to all healthcare settings as this implementation study targeted at community hospitals with medium levels of resources and staff available.

For peer review only

INTRODUCTION

Despite fast-growing literature on the effectiveness and potential economic impact of antimicrobial stewardship (AMS) in healthcare facilities around the world [1-5], limited data from hospitals in low- and middle-income countries (LMICs) show numerous challenges in these settings and that implementation must be tailored to the local contexts to have an effect on antibiotic use and antimicrobial resistance (AMR).[6 7] Viet Nam, like many other LMICs, is affected by high and increasing levels of AMR. Surveillance data from Viet Nam's AMR surveillance network show that the prevalence of antibiotic resistance is among the highest in Asia and the world. [8-10] Hospital-wide antibiotic consumption data from this hospital network also showed critical broad-spectrum and last-resort antibiotics are increasingly used as empiric treatment. In a 12-month point prevalence survey at 15 adult intensive care units within the AMR surveillance network in 2012, carbapenems were the third most commonly used class in all patients and the most common among those with hospital acquired infections.[11]

The Ministry of Health (MoH) in Viet Nam developed a National Action Plan on AMR in 2013 for 2013-2020. Surveillance and antimicrobial stewardship (AMS) programs were 2 of the 6 listed priorities of the plans. A nation-wide AMR hospital surveillance network and a reference laboratory have been established.[9 12] A national AMS implementation guideline was issued in 2016 [13] and updated in 2020 [14] which required each hospital to establish a multidisciplinary AMS team and implement a set of recommended activities. However, many hospitals continue to struggle to implement AMS despite implementation guidance from the Viet Nam MoH.[15] In a recent survey of 655 hospitals across the country by the MoH in 2019, 144 (22%) had developed treatment guidelines based on local evidence, 269 (41%) had adopted the pre-authorization policy (antibiotics requiring approval before use for patient treatment), 189 (29%) developed clinical microbiology guidelines, and 458 (70%) employed basic infection control policy in their facilities.[15]

In our previous qualitative study about the AMS implementation processes in seven hospitals in the surveillance network, we identified the following barriers and opportunities that hospitals in Viet Nam have encountered in setting up and running the programs: a lack of dedicated resources, ineffective communication between

For peer review only - http://bmjopen.bmj.com/site/about/guidelines.xhtml

clinicians, pharmacy and microbiology department, and prescribing habits among doctors, including not taking diagnostic samples before starting antibiotic therapy, not adjusting antibiotics based on culture results, and hesitation and lack of confidence in de-escalating and switching from intravenous to oral therapy. Antibiotics were mainly prescribed based on clinical presentation and progress, doctors' experience, the requirements for health insurance reimbursements and availability of specific drugs at the pharmacy department (manuscript under preparation). Limited data are available on the process of implementation and impact of AMS programs in Viet Nam; only one report from a large hospital has indicated AMS activities could improve patient outcomes.[16]

Here, we describe implementation of an intervention package that included action-planning, educational interventions and data feedback to assess the feasibility and potential impact of AMS programs in provincial hospital settings in Viet Nam.

METHODS

Implementation approach

Our planning process began at a national AMS stakeholder meeting organized by the Medical Services Administration of the MoH, World Health Organization and Oxford University Clinical Research Unit (OUCRU), during which we reviewed the current status of AMS implementation in the country. Based on our pre-existing understanding and network with local hospitals, on this meeting, and on subsequent discussions with MoH, we invited two provincial hospitals to join this implementation study: Viet Tiep Hospital in Hai Phong, a large port city with 4 million inhabitants, in the north (2,000-bed capacity, 400,000 out-patient and 100,000 in-patient visits per year) and Dong Thap General Hospital in Cao Lanh, Dong Thap in the Mekong Delta in the south (1000-bed capacity, 710,000 out-patient and 65,000 in-patient visits per year).

The process of setting up an AMS program at each hospital went through cycles of planning, implementing and evaluating phases (Figure 1). This was a participatory action process with hospitals regarded as a complex adaptive system consisting of evolving inter-related individuals and non-linear interactions, and conducted under the assumption that interventions cannot be applied from one hospital to another with

For peer review only - http://bmjopen.bmj.com/site/about/guidelines.xhtml

predictable results.[17] During discussions between the hospitals and the study team, we identified elements of an AMS program that should be common to both hospitals and elements that could be adapted locally. The results of interventions over time were monitored through repeated evaluations of prescription practices, knowledge and attitudes every three months, and interventions could evolve over time to achieve the maximal impact. The AMS team regularly used the collected data and discussed with the study team to identify issues and opportunities for improvement.

Each hospital selected eight clinical wards to participate in the study: four for AMS intervention and four as control wards (the number of wards was determined by the funding availability). The selection of intervention and control wards was based on discussions within the hospital AMS team with two selection criteria: 1) the amount of antibiotic used was greater than average of all wards in the hospital based on pharmacy reported data and 2) the head of ward was willing to participate in the study intervention or control group. The control wards were included to support evaluation of overall AMS outcome indicators (total antibiotic use and for main classes) using trend analysis over 12 months before and after the start of intervention. For practical reasons, there was no strict requirement to identify control wards that were similar to the intervention wards. After eight wards were identified, the decision on the wards to be in the intervention group and control group was left at the discretion of the director boards and the AMS team in each hospital.

The preparation and implementation process started with the training and mentoring component, which was then integrated throughout the study life cycle. Training, technical support and mentoring activities were modeled to international AMS models and developed with experts from Duke Antimicrobial Stewardship Outreach Network (DASON) in Durham, North Carolina, USA, and included sharing experiences and identifying opportunities for local changes and interventions to improve antibiotic use and patient outcomes. DASON is community hospital network that supports the implementation of local AMS programs and collaborative network participation has shown reductions in antibiotic use in these hospitals.[18 19]

Trainings and mentoring by local experts on infectious diseases, clinical pharmacists, and clinical microbiologists to support AMS implementation were also integrated into

For peer review only

the programs. These experts were from National Hospital for Tropical Diseases, Hanoi; Hospital for Tropical Diseases, Ho Chi Minh City, Viet Nam National Children's Hospital, Hanoi and Hanoi University of Pharmacy, Hanoi. Local meetings were organized in each hospital with the participation of AMS team members and participating intervention wards to assess the current gaps and needs and develop plans for implementation.

Evaluation as part of the implementation cycle

Evaluation was integrated throughout starting with the first round of data collection and analysis before the AMS intervention and every 3 months after program implementation. The data were used to design and tailor the intervention activities toward the goal in reducing unnecessary antibiotic use in each hospital. Following baseline indicators were evaluated in each hospital (Supplementary Table 1):

- Antibiotic use in total and from targeted agent groups;
- Knowledge and perceptions of doctors;
- Proportions of antibiotic prescriptions which had an indication documented, had review/stop date documented, were used as surgical prophylaxis for greater than 24 hours, were not compliant with guidelines currently endorsed at the hospital, and were inappropriate;
- Proportion of resistant isolates for priority antibiotic-organism combinations;
- Cost of antibiotic treatment and hospitalization;
- In-hospital mortality;
- Hospital length of stay.

Data were from the existing routine hospital information systems for 12 months baseline period, knowledge-attitude-practice (KAP) surveys of all doctors in 8 study wards and relevant AMS staff (pharmacists and microbiologists), and reviews of medical charts in the 4 intervention wards by the doctors at each of the wards.

The key process indicators of the project were: 1) a functioning AMS team was formed and was able to lead the AMS activities; 2) a 1-year implementation plan for AMS program was developed and carried out; 3) baseline data and subsequent data from evaluation rounds were used to inform planning and implementation. We

For peer review only - http://bmjopen.bmj.com/site/about/guidelines.xhtml

recorded implementation data to monitor the intervention activities, specifically the prospective audit and feedback (PAF) implemented by the clinical pharmacists. Other data about trainings and meetings (quantity, number of participants, content) and resources required for the activities (costs, time, staff) were also recorded for monitoring the process of program implementation and support parallel economic evaluation.

Data analysis and feedback

We reviewed contextual information collected from the national meeting and the baseline needs assessment at the two hospitals and performed an analysis of strengths, weaknesses, opportunities and threats (SWOT) of AMS implementation to support the AMS team in initial planning of the activities at each hospital. We used the following DASON recommendations for the key AMS components to guide our analysis, which include the core elements described by the US CDC [20] and the global recommendations[21]:

Leadership: An individual who is qualified through education, training, or experience in infectious diseases (ID) and/or antibiotic stewardship as leader of AMS program; Committee structures for oversight and executive authority for AMS activities; Formal policy outlining AMS program

Pharmacy: Automated systems (automated dispensing system, bar-code medication administration, computerized physician order entry, electronic medical record, electronic medication administration record); Pharmacy-based dose optimization activities; Regular antibiotic use evaluations; Antibiotic formulary; Pharmacy staffing with clinical pharmacist with specialized training and interest in ID

Microbiology: Laboratory reporting system; Cumulative antibiogram; Microbiology personnel; Testing and incidence of multi-drug resistant organisms

Infection management: ID clinician presence

We performed descriptive data analysis for each hospital on baseline data and data from the subsequent evaluation rounds to identify issues that required attention and opportunities for intervention in the AMS program. Results were summarized and presented to provide feedback to the hospital management board, AMS team, and intervention wards through reports and project progress meetings and to use as evidence for identifying further actions. We characterized antibiotic use by subgroups,

For peer review only - http://bmjopen.bmj.com/site/about/guidelines.xhtml

types of clinical wards, and international classification of diseases (ICD-10) code groups. Hospital-wide antibiotic susceptibility testing results entered into WHONET were extracted and analyzed following the same analysis protocols performed in previous studies for AMR surveillance data from VINARES hospital network.[9 10] We reported microbiology data following the recommendations of the MICRO checklist [22] as shown in Supplementary Table 2.

Resistance results were expressed as proportions of resistant isolates out of the number of tested isolates. We classified multidrug resistance (MDR) and carbapenem resistance based on the criteria proposed by Magiorakos et al [23] as follows:

- For *Acinetobacter baumannii* and *Acinetobacter spp.*: MDR is defined as resistant to at least 3 of the following: cephalosporin (ceftriaxone or cefepime), aminoglycosides (amikacin, gentamicin or tobramycin), ciprofloxacin and carbapenem (imipenem or meropenem); carbapenem resistance is defined as resistant to imipenem or meropenem;
- For *Escherichia coli, Klebsiella pneumoniae,* and *Klebsiella spp.*: MDR is defined as resistant to at least 3 of the following: carbapenem (ertapenem, imipenem or meropenem), cephalosporin (ceftriaxone or cefepime), aminoglycosides (amikacin, gentamicin or tobramycin) and ciprofloxacin; carbapenem resistance is defined as resistant to ertapenem, imipenem, or meropenem;
- For *Pseudomonas aeruginosa*: MDR is defined as resistant to at least 3 of the following 4 agents: imipenem, ceftazidime, ciprofloxacin and tobramycin; carbapenem resistance is resistant to imipenem or meropenem;

Ethical approval

The study protocol was approved by the Oxford University Tropical Research Ethics Committee (OxTREC Reference 526-19), and the Ethics Committee of National Hospital of Tropical Diseases (08/HĐĐĐ-NĐTƯ 31 May 2019). The conduct of this study conformed to the principles embodied in the Declaration of Helsinki.

Patient and Public Involvement

Patient and public involvement was not included in this study as this is a new aspect in the local hospital settings and requires additional research to explore its feasibility

For peer review only - http://bmjopen.bmj.com/site/about/guidelines.xhtml

and the most appropriate approaches. Initial efforts are being undertaken in parallel to this research for involving patients and the public in AMS in the local hospitals.

Here we present the results of the initial steps in setting up the program and describe the baseline summary indicators from the routine data of the two hospitals. This paper was reported following the StaRI checklist "Standards for Reporting Implementation Studies: the StaRI checklist for completion".[24]

RESULTS

1. Baseline analysis from the national AMS stakeholder meeting

The meeting was organized in March 2019 to review the current state of AMS implementation nation-wide with participation of a wide range of stakeholders representative for both public and private sectors, and governmental and international partners working or interested in AMS implementation (Supplementary Table 3). The meeting opened a platform for shared discussions on different aspects of implementation and identified the three major areas for actions at the national level: strengthening a supportive policy environment for AMS, developing AMS training curriculum and core AMS related competencies, and establishing coordination and collaboration in AMS within Viet Nam and the region. Further stakeholder meetings were recommended for continued sharing of experience and promote networking and collaboration in AMS locally and regionally, with one such meeting subsequently organized in December 2019 by OUCRU.

2. Baseline analysis at two hospitals and implementation plan

Data on existing processes and structures were reviewed to provide input for initial planning of AMS intervention. In Table 1, we summarized the results of a SWOT analysis conducted during the preparation phase at both hospitals in 2020 of the current state of key components, the needs identified for each component, and the corresponding AMS interventions to be implemented. There was a large gap between the baseline situation in each hospital and the recommendations by DASON in all components. Leadership commitment was identified as a prerequisite for site selection, however the level of participation was different between the two hospitals. At one hospital, a vice director directly leads the AMS program together with the General Planning Department, which drives the implementation activities and

For peer review only - http://bmjopen.bmj.com/site/about/guidelines.xhtml

encourages the clinical wards to actively be engaged in the program. The process was slower at the other hospital where the program was managed directly by the General Planning Department and International Collaborations Department.

Both hospitals provide care and treatment to all types of clinical conditions and diseases in the catchment areas. Each hospital has a department of infectious diseases, providing care and treatment to patients who are admitted to this department, and provide consultation in managing patients with signs of infection in other clinical wards if required. Each hospital also has a department of pharmacy and a microbiology laboratory. In both hospitals, pharmacists were mostly involved in drug inventory and bidding, and some support to doctors in dose adjustment in special cases, and microbiologists' main roles were to inform doctors of test results. Engagement of microbiology, pharmacy and infectious diseases departments in antibiotic treatment was generally passive, mostly through hospital-wide consultation meetings on an ad-hoc basis when there were difficult cases. Local AMR surveillance data was not readily available for use to inform treatment guidelines or updating drug formularies.

From the gaps and needs identified, specific strategies were developed within the first-year plan for interventions targeting each of the key components (Table 1).

These strategies included: 1) establishing leadership commitment and forming an AMS coordinating team that can connect all relevant wards and units within each hospital. At both hospitals, the planning department plays a key and coordinating role with a linkage to the specific clinical and functional departments and reporting responsibility to the hospital management board; 2) using data to inform planning and evaluating AMS activities and stimulate the actions among leaders and staff through regular AMS meetings organized at each hospital. Data were from the KAP surveys, review of patient medical charts and routine data on antimicrobial treatment, clinical diseases, antimicrobial susceptibility data and denominator data (days present at each study ward); 3) training activities to update the knowledge and skill gaps for doctors in the intervention wards based on the findings from quarterly KAP surveys and retrospective reviews of patients charts for antibiotic prescribing practices. These trainings were delivered by national experts on infectious diseases, clinical

For peer review only

microbiology and clinical pharmacy; technical training for AMS staff on specific skills including setting up an AMS program, using data for planning and monitoring, prospective audit and feedback, communication skills, and developing guidelines provided by DASON experts and local experts; 4) implementing prospective audit and feedback (PAF) at the intervention wards by clinical pharmacists with support from the AMS doctor and team members. On-site training and shadowing experienced clinical pharmacists in HTD during their PAF activities were organized to develop the capacity for these new teams.

Some strategies differed by site. At Hospital 1, due to limited microbiology data available for developing treatment guidelines the hospital decided to focus firstly on increasing the quantity and quality of specimen cultures through updating the current SOPs and organizing trainings for technicians and nurses at each clinical ward. At Hospital 2 where there are more surgical wards, the hospital AMS team put more focus on surgical prophylaxis with an initial aim to reduce the number of two-drug combination orders.

3. Baseline summary indicators before intervention

Antibiotic use data

Total number of patients admitted at the intervention and control wards at the two hospitals in 2019 and the proportions with antibiotic treatment during the whole hospital admission are summarized in Table 2 following the ICD-10 codes for the main diagnosis recorded for each patient. The spectrum of diseases was by definition heterogeneous among the patients admitted at the intervention and control wards but also in both absolute number and the proportions being prescribed antibiotics. The diagnosis groups with highest amount of antibiotic use in DOT in 2019 for both intervention and control group in two hospitals also differed. The top three for each group were (in brackets, the average number of DOT per 1000 days present):

- Control group in Hospital 1: genitourinary diseases (1057), respiratory diseases (1028), and certain infectious/parasitic diseases (927);
- Intervention group in Hospital 1: respiratory diseases (897), certain infectious/parasitic diseases (863), and injury, poisoning and certain other consequences of external causes (729);

For peer review only - http://bmjopen.bmj.com/site/about/guidelines.xhtml

- Control group in Hospital 2: digestive diseases (800), certain infectious/parasitic diseases (797), and neoplasms (553);
- Intervention group in Hospital 2: respiratory diseases (888), injury, poisoning and certain other consequences of external causes (736), and digestive (707).

Figure 2 displays the monthly distribution of antibiotic use in patients in the intervention and control wards. The amount of antibiotic consumption varied significantly by month in 2019 in the study wards for both hospitals: 951 DOT per 1000 days present in the control compared to 496 in the intervention group in Hospital 1, and 737 in the control compared to 714 in the intervention group in Hospital 2. Hospital 1 used more third-generation cephalosporins than Hospital 2 while Hospital 2 used more penicillin combinations than Hospital 1. In addition, there were more variations across the months in the amount of use for each antibiotic subgroup in Hospital 2 than Hospital 1.

Microbiology data

There were 2430 isolates reported from culture of 10,784 specimens (all types of specimens, 22.5% positive rate) in Hospital 1, with 289 isolates from 3583 blood specimens (8.1% positivity rate) in 2019. Hospital 2 reported 5463 isolates recovered from 23,731 specimens (all types of specimens, 23.0% positive rate) and 1642 isolates recovered from 14,539 blood specimens (11.3% positive rate) in 2019.

Among the study wards, there were 1392 isolates (5.5 isolates per 1000 days present) reported for eight study wards in Hospital 1 (198 blood isolates, 14.2%) in 2019. Slightly more isolates were from male patients (56.6%) and from older age range (median age 63; interquartile range 48-74) in the 8 wards of this hospital. Hospital 2 reported 2433 isolates (13.4 isolates per 1000 days present) for eight study wards in 2019, of which 802 (33.0%) were blood isolates. These isolates were also mainly from male patients (64.3%) and older age range (median age 64; interquartile range 52-76).

E. coli was the most commonly isolated bacterium in the study wards in both hospitals (Figure 3), and harboured 8.3% (n=540) carbapenem resistance and 34.1% (n=539) MDR (resistant to at least 3 of the following: carbapenem, cephalosporin,

For peer review only - http://bmjopen.bmj.com/site/about/guidelines.xhtml

aminoglycosides, and ciprofloxacin) in Hospital 1 and 7.4% (n=297) and 20.6% (n=267) respectively in Hospital 2 (Supplementary Table 4). *K. pneumoniae* in Hospital 2 showed a high level of resistance to carbapenem (in all specimens 52.2% n=289 and in blood 45.2% n=62) and MDR (51.0% n=249 and 44.1% n=59 respectively). For Hospital 1, *Klebsiella spp.* showed lower carbapenem resistance (26.3% n=167) and MDR (39.5% n=167) in all specimens.

High levels of carbapenem resistance in all specimens (80.0% n=325) and in blood (57.9% n=19) and MDR (resistant to at least 3 of the following: cephalosporin, aminoglycosides, ciprofloxacin and carbapenem) in all specimens (62.6% n=265) and in blood (47.1% n=17) were reported for *A. baumannii* in Hospital 2 and *Acinetobacter spp.* in Hospital 1 (in all specimens 64.1% n=103 carbapenem resistance and 60.8% n=102 MDR).

For *P. aeruginosa* in all specimens, the proportions were 25.3% n=253, 35.0% n=254 and 24.3% n=272 for ceftazidime resistance, carbapenem resistance and MDR (resistant to at least 3 of the following 4 agents: imipenem, ceftazidime, ciprofloxacin and tobramycin), respectively in Hospital 2, lower than those in Hospital 1 (41.2% n=85, 41.9% n=86 and 40.7% n=86 respectively).

Proportions of methicillin-resistant *Staphylococcus aureus* were 79.0% (n=210) in Hospital 1 and 76.7% (n=103) in Hospital 2 based on the cefoxitin test.

DISCUSSION

We used an implementation research approach to evaluate the feasibility and understand the process of AMS implementation in two pilot hospitals in Viet Nam. Firstly, this study showed that building a functioning multidisciplinary AMS team is an important factor for the success of the program. In Viet Nam, AMS committees have been formed in 334 out of 655 hospitals (51%) in a recent MoH report in 2019, however the majority of these committees had irregular activity (72%).[15] In our study, AMS teams were led by hospital director/vice-director and the planning department, which has the advising role to the director-board. These combinations were routed from the traditional set-up of Vietnamese hospitals and appeared to work effectively in most hospital-wide programs. SWOT analysis, a recommended tool in the WHO

For peer review only - http://bmjopen.bmj.com/site/about/guidelines.xhtml

Resources for AMS programs in health-care facilities in LMICs[6], was used in our needs assessment and has proved to effectively help our hospital AMS teams identify the needs and focus priority interventions.

Secondly, the partnership approach combining local and international partners in our study demonstrated an effective synergy of experience, expertise and resources to ensure the appropriate selection of strategies and methods in setting up AMS programs in LMICs. This approach has been advocated in a recent call for international collaborations in AMS implementation in LMICs, which can be achieved through the dissemination of high-quality research and educational resources recognizing the needs to contextualize to the local conditions.[25]

Across a number of AMS programs worldwide, pharmacists are often included to manage the prescription-based strategies including pre-authorization formularies and post-prescription audits and feedback (PAF). This was reflected in the national guideline on AMS implementation originally issued in 2016 and updated in 2020.[13 14] Sustained reductions have been achieved in antibiotic use by AMS teams across different resource settings including the example of non-specialist pharmacist-led program.[26] In our study, despite having no experience in clinical interactions, our junior pharmacists have been building their PAF capacity and gained promising results in the number of audits with feedback to the doctors: 726 cases reviewed with 10% having feedback to prescribers in Hospital 2 in 4 months (4 fulltime clinical pharmacists) compared to 977 cases with 3% feedback in 6 months in Hospital 1 (1.5 fulltime clinical pharmacists). Although the results were highly variable between hospitals, this shows the feasibility and potential of involving pharmacists in the prescription-based strategies for AMS programs in LMICs. A recent prospective multicenter study in community hospitals in the USA has also shown the effective role of pharmacists in implementing the pre-authorization and post-prescription audit and feedback in improving antibiotic use in resource-limited settings.[27]

AMS programs in LMICs generally encounter challenges of limited financial and physical resources available for implementation including substandard lab practices, lack of well-trained staff, insufficient opportunities for continuing professional development, and difficulties in deploying prescription-based stewardship strategies.[25]

For peer review only - http://bmjopen.bmj.com/site/about/guidelines.xhtml

[26] Despite numerous challenges and limited, poor-quality evidence available [28], effective AMS programs can be feasible in these settings although contextualization is essential in this implementation.[7] The need for contextualization in LMICs was also highlighted in several qualitative and mixed-methods AMS studies.[29-31] In our program, AMS implementation relied on external financial support with the funded intervention period planned for one year. Considerations were made regarding choosing the most effective strategies that can support ongoing AMS team operations and continued AMS activities after the funding period ends.

Among these considerations, capacity building and training for the AMS team in program planning and evaluation and hospital pharmacists to conduct PAF activities were identified as the key components. As recommended in a recent review of AMS program designs, practically integrating the planned changes into the normal routine clinical practices is the best way to achieve sustainability apart from securing long-term resources.[32] Making PAF activities routine can contribute to the sustainability of the AMS program. In addition, developing methods and staff capacity to utilize the existing routinely collected data (clinical, pharmacy, and microbiology) was also a priority in our program. Nevertheless, at both study hospitals, the outstanding issue remained in the use of local evidence to inform practices in antibiotic treatment and management, and a lack of information technology and epidemiological capacity partly contributes to this issue. This issue will need to be addressed in the next longer-term AMS program plan at both hospitals.

AMS programs have been traditionally “pharmacy-centric” with minimal or no input from microbiology; participation of clinical microbiologists in AMS teams varied widely around the globe (20-90% across the surveys in Europe, Australia, US and Canada).[33] Microbiology capacity and surveillance and control of multi-drug resistant bacteria is a common challenge in AMS implementation for hospitals in LIMCs [32 34], and this is particularly the case for provincial and community level hospitals in Viet Nam. From the MoH survey in 2019, 90% of hospitals had a pharmacist while only 39% included a microbiologist in their AMS team. The current status of microbiology in LMICs would not allow for a desirable level of support to AMS programs.[25] However, improvements can be made even within resource-constrained conditions. Our study showed that simply compiling the current data and presenting the summary

For peer review only

For peer review only - http://bmjopen.bmj.com/site/about/guidelines.xhtml

data in the AMS meetings has stimulated the discussions on the immediate and long-term actions to improve the problem. The participants realized the need to have good microbiology data as a foundation for more effective and targeted interventions, and the first simple step was to review the procedures, organize trainings, and encourage doctors to perform specimen cultures prior to initiating antibiotic treatment. Such changes have been made feasible by an active AMS team with motivated team leaders which is considered as a prerequisite for the success of AMR containment interventions in LMIC settings.[35]

The strengths of our study include the participatory approach in AMS implementation where hospitals were considered as a complex adaptive system with unique characteristics and interconnections between players that could be leveraged to develop locally tailored interventions for maximal results.[17] These included identifying strategies that could be sustained relying on local resources as routine practices including PAF and data use. Secondly, the study has employed inter-disciplinary and team activities in both implementation and evaluation of AMS program, and emphasized the need to improve the capacity of all stakeholders involved in each hospital setting. Thirdly, our implementation approach was designed to include perspectives and expertise from both policy makers to hospitals at different levels and from international and local partners. Our data from the implementation process were consistent with and consolidating the outcomes from the discussions in the national stakeholder meeting.

Our study, however, shares the common limitation of implementation research, which is the ability to generalize the obtained knowledge of AMS implementation to different healthcare settings. Our evidence in two provincial hospitals in Viet Nam will be generalizable to the community hospitals with medium levels of resources and staff available for implementation but lack of staff training and capacity in carrying out AMS activities. Nevertheless, the nature of implementation research in recognizing and tailoring activities to the local contexts is an important aspect in bridging the gap in our understanding of what is known to be effective and what works in the actual patient care settings.[17] Further implementation research in other hospital and healthcare settings is required to provide more data to fill the know-how gap in AMS programs in LMICs.

For peer review only - http://bmjopen.bmj.com/site/about/guidelines.xhtml

In conclusion, AMS implementation requires an in-depth understanding of the local conditions and practices and the contextualization of the intervention strategies in Viet Nam and similar LMIC settings. We showed that using implementation research design is an effective approach in assessing the feasibility and evaluation of the AMS program. It also helps local hospitals understand their needs and start prioritizing AMS in the local agenda, paving the solid steps for a long-term institutionalization and improvement of the program. As evident in our process, it is important to develop the skills and capacity of the multidisciplinary AMS team and identify creative combinations of strategies that could be carried out with locally available resources for maximal and sustainable outcomes.

Acknowledgements

We acknowledge the invaluable support and collaboration of the Medical Services Administration of the Viet Nam Ministry of Health and the World Health Organization Office in Viet Nam during the planning of this study.

Funding

Pfizer Independent Grants for Learning & Change (IGLC) provides all project funding and The Joint Commission provides administrative oversight for the program; H.V.T.L was also supported by the National Institute for Health Research (NIHR) (using the UK's Official Development Assistance (ODA) Funding) and Wellcome (Grant Reference Number: 216367/Z/19/Z) under the NIHR-Wellcome Partnership for Global Health Research. The views expressed are those of the authors and not necessarily those of Wellcome, the NIHR or the Department of Health and Social Care.

Competing interests

None declared.

Contributors

VTLH, HRvD, and DJA obtained funding and contributed to all aspects of the study design, with input from LMQ, VHV, and CMD. VTLH and HRvD had overall responsibility for the study. TTTH, VHV, VTHDE, TTDN and HPT coordinated the

For peer review only

running of the study with support from NTH, TD, PVBB, NTTO, PTTH, NTCT, and TAQ. VTLH analysed the data with input from HRvD, DJA, EDA, and TK. VTLH was responsible for the drafting of the manuscript. All authors gave approval for the final version of the manuscript.

Patient consent for publication

Not required for this research.

ORCID iDs

Vu Thi Lan Huong 0000-0002-9579-5576

H. Rogier van Doorn 0000-0002-9807-1821

Data availability

De-identified antibiotic use and resistance baseline data may be obtained from the hospitals participating in this study when a data sharing agreement is in place.

For peer review only

REFERENCES

1. Schuts EC, Hulscher MEJL, Mouton JW, et al. Current evidence on hospital antimicrobial stewardship objectives: a systematic review and meta-analysis. The Lancet Infectious Diseases 2016;**16**(7):847-56.

2. Davey P, Marwick CA, Scott CL, et al. Interventions to improve antibiotic prescribing practcies for hospitals inpatents. Cochrane Database Syst Rev 2017;**2: CD003543**.

3. Wagner B, Filice GA, Drekonja D, et al. Antimicrobial Stewardship Programs in Inpatient Hospital Settings: A Systematic Review. Infection Control & Hospital Epidemiology 2016;**35**(10):1209-28.

4. Dik J-WH, Vemer P, Friedrich AW, et al. Financial evaluations of antibiotic stewardship programs—a systematic review. Frontiers in Microbiology 2015;**6**(317).

5. Kaki R, Elligsen M, Walker S, et al. Impact of antimicrobial stewardship in critical care: a systematic review. Journal of Antimicrobial Chemotherapy 2011;**66**(6):1223-30.

6. Pierce J, Apisarnthanarak A, Schellack N, et al. Global Antimicrobial Stewardship with a Focus on Low- and Middle-Income Countries. Int J Infect Dis 2020;**96**:621-29.

7. Cox JA, Vlieghe E, Mendelson M, et al. Antibiotic stewardship in low- and middle-income countries: the same but different? Clin Microbiol Infect 2017;**23**(11):812-18.
8. Kim SH, Song JH, Chung DR, et al. Changing trends in antimicrobial resistance and serotypes of Streptococcus pneumoniae isolates in Asian countries: an Asian Network for Surveillance of Resistant Pathogens (ANSORP) study. Antimicrob Agents Chemother 2012;**56**(3):1418-26.
9. Vu TVD, Do TTN, Rydell U, et al. Antimicrobial susceptibility testing and antibiotic consumption results from 16 hospitals in Viet Nam: The VINARES project 2012–2013. J Glob Antimicrob Resist 2019;**18**:269-78.
10. Dung VTV, et al. Antimicrobial susceptibility testing results from 13 hospitals in Viet Nam: VINARES 2016-2017. Antimicrob Resist Infect Control 2021.
11. Phu VD, Wertheim HFL, Larsson M, et al. Burden of Hospital Acquired Infections and Antimicrobial Use in Vietnamese Adult Intensive Care Units. PLoS One 2016;**11**(1):e0147544.
12. Kinh NV, Wertheim HFL, Thwaites GE, et al. Developing an antimicrobial resistance reference laboratory and surveillance programme in Vietnam. Lancet Glob Health 2017;**5**(12):e1186-e87.
13. Viet Nam Ministry of Health. Quyết định về việc ban hành tài liệu "Hướng dẫn thực hiện quản lý sử dụng kháng sinh trong bệnh viện" [Decision on the issuance of the document "Guideline on implementing antimicrobial stewardship in hospitals"]. Hanoi: Viet Nam Ministry of Health, 2016.
14. Viet Nam Ministry of Health. Quyết định về việc ban hành tài liệu "Hướng dẫn thực hiện quản lý sử dụng kháng sinh trong bệnh viện" [Decision on the issuance of the document "Guideline on implementing antimicrobial stewardship in hospitals"] Numbered 5631/QD-BYT. In: Health Mo, ed. Ha Noi: Ministry of Health, 2020.
15. Hanoi Hospital Pharmacy Association, ed. Thực trạng triển khai chương trình quản lý sử dụng kháng sinh tại các bệnh viện trên toàn quốc [Status of implementing antimicrobial stewardship programs in hospitals across Viet Nam]. Open Hanoi Hospital Pharmacy Scientific Conference; 2019; Hanoi. Hanoi Hospital Pharmacy Association.
16. Son NT, Tra TT, Thao PTN. Antimicrobial Stewardship Program at a tertiary teaching hospital in Vietnam: A longitudinal observational study. Clin Microbiol Infect Dis 2017;**2**:in press.

For peer review only

For peer review only - http://bmjopen.bmj.com/site/about/guidelines.xhtml

17. Leykum LK, Pugh Ja Fau - Lanham HJ, Lanham Hj Fau - Harmon J, et al. Implementation research design: integrating participatory action research into randomized controlled trials. Implement Sci 2009;**4**(69).

18. Sexton DJ, Moehring RW. Implementation of Antimicrobial Stewardship Programs in Small Community Hospitals: Recognizing the Barriers and Meeting the Challenge. Clin Infect Dis;**65**(4):697-98.

19. Yarrington M, Dodds Ashley E, Johnson M, et al. 2089. Effect of the Duke Antimicrobial Stewardship Outreach Network (DASON): A Multi-Center Time Series Analysis. Open Forum Infectious Diseases 2019;**6**(Suppl 2):S705-S06.

20. (CDC) CfDCaP. Core Elements of Hospital Antibiotic Stewardship Programs, https://www.cdc.gov/antibiotic-use/core-elements/hospital.html. 2019.

21. Pulcini C, Binda F, Lamkang AS, et al. Developing core elements and checklist items for global hospital antimicrobial stewardship programmes: a consensus approach. Clin Microbiol Infect 2018.

22. Turner P, Fox-Lewis A, Shrestha P, et al. Microbiology Investigation Criteria for Reporting Objectively (MICRO): a framework for the reporting and interpretation of clinical microbiology data. BMC Med 2019;**17**(1):70.

23. Magiorakos AP, Srinivasan A, Carey RB, et al. Multidrug-resistant, extensively drug-resistant and pandrug-resistant bacteria: an international expert proposal for interim standard definitions for acquired resistance. Clin Microbiol Infect 2012;**18**(3):268-81.

24. Pinnock H, Barwick M, Carpenter CR, et al. Standards for Reporting Implementation Studies (StaRI) Statement. BMJ 2017;**356**(i6795).

25. Tattevin P, Levy Hara G, Toumi A, et al. Advocacy for Increased International Efforts for Antimicrobial Stewardship Actions in Low-and Middle-Income Countries on Behalf of Alliance for the Prudent Use of Antimicrobials (APUA), Under the Auspices of the International Society of Antimicrobial Chemotherapy (ISAC). Front Med (Lausanne) 2020;**7**:503-03.

26. Laxminarayan R, Van Boeckel T, Frost I, et al. The Lancet Infectious Diseases commission on antimicrobial resistance: 6 years later. Lancet Infect Dis 2020;**20**(4):e51-e60.

27. Anderson DJ, Watson S, Moehring RW, et al. Feasibility of Core Antimicrobial Stewardship Interventions in Community Hospitals. JAMA Netw Open 2019;**2(8)**(2574-3805 (Electronic)):e199369.

For peer review only - http://bmjopen.bmj.com/site/about/guidelines.xhtml

28. Van Dijck C, Vlieghe E, Cox JA. Antibiotic stewardship interventions in hospitals in low-and middle-income countries: a systematic review. Bull World Health Organ 2018;**96**(4):266-80.
29. Charani E, Smith I, Skodvin B, et al. Investigating the cultural and contextual determinants of antimicrobial stewardship programmes across low-, middle- and high-income countries-A qualitative study. PLoS One 2019;**14**(1):e0209847.
30. Gebretekle G, Haile Mariam D, Abebe W, et al. Opportunities and barriers to implementing antibiotic stewardship in low and middle-income countries: Lessons from a mixed-methods study in a tertiary care hospital in Ethiopia. PLoS One 2018;**13**(12):e0208447.
31. Mathew P, Ranjalkar J, Chandy SJ. Challenges in Implementing Antimicrobial Stewardship Programmes at Secondary Level Hospitals in India: An Exploratory Study. Front Public Health 2020;**8**(542):493904.
32. Resman F. Antimicrobial stewardship programs; a two-part narrative review of step-wise design and issues of controversy Part I: step-wise design of an antimicrobial stewardship program. Ther Adv Infect Dis 2020;**7**:2049936120945083.
33. Morency-Potvin P, Schwartz DN, Weinstein RA. Antimicrobial Stewardship: How the Microbiology Laboratory Can Right the Ship. Clin Microbiol Rev 2017;**30**(1):381.
34. Nkengasong JN, Yao K, Onyebujoh P. Laboratory medicine in low-income and middle-income countries: progress and challenges. Lancet 2018;**391**(10133):1873-75.
35. Singh N, Manchanda V. Control of multidrug-resistant Gram-negative bacteria in low- and middle-income countries-high impact interventions without much resources. Clin Microbiol Infect 2017;**23**(4):216-18.

Tables

For peer review only

For peer review only - http://bmjopen.bmj.com/site/about/guidelines.xhtml

Table 1. SWOT analysis of current status of key components, assessment of needs and proposed interventions at two provincial hospitals

Component	SWOT analysis (Strength – Weakness – Opportunity – Threat)	Assessment of needs	Proposed interventions
**Hospital 1**			
Leadership	S: Leadership commitment; Vice-director had an active role in AMS committee;<br>W: No AMS experience, large committee with no dedicated AMS coordinator for day-to-day activities<br>O: Guidelines from MoH and experience from other higher-level hospitals; leadership intention to provide AMS outreach support to district hospitals in long term<br>T: Disease outbreaks (e.g. COVID-19) shifting management priorities	Knowledge and skills in planning and implementing AMS;<br>Identify clear roles in planning and implementing AMS activities;<br>Identify targets for interventions	Establishing AMS action team for day-to-day activities with active coordination from Planning Department;<br>Regular minuted team meetings;<br>Training for AMS committee by international and local experts;<br>Collecting and reviewing data to inform interventions
Pharmacy	S: Drug inventories and bidding;<br>W: No clinical experience; only tracking drug purchasing but not actual drug use data; lack of analytical skills<br>O: Electronic data on patient-level drug administration available from clinical database; Government policy and guidelines (clinical pharmacy, AMS guidelines)<br>T: High staff turnover, lack of clinical pharmacists, bidding process and insurance limits (can affect drug choices and prescribing behaviors)	Identify staff for clinical pharmacist roles;<br>Build clinical pharmacy capacity and data analysis skills	Training for clinical pharmacists in antibiotic treatment, microbiology, pharmacology, and review of antibiotic prescriptions (class-room, hands-on);<br>Implemented prospective audit and feedback as a routine activity of clinical pharmacy program;<br>Participation of clinical pharmacists in clinical ward monthly meeting for which they had to analyze audit data and present to the doctors;<br>Support from DASON on using web-based data visualization and benchmarking tool;
Microbiology	S: In-house microbiology lab; technical support by OUCRU; use WHONET database<br>W: Contamination issues, low test utilization; lack of clinical interactions; no clinical microbiologist; performing manual culture; limited quality control<br>O: Close connection with OUCRU lab; a new lab under construction<br>T: Lack of compliance in sample collection from clinical wards; Weak voice in planning and ordering reagents and tests; insurance limits (can affect culture ordering behavior)	Improve quantity and quality of routine clinical specimens, and quality of microbiology testing;<br>Active planning from microbiology lab;<br>Active communication from lab to clinical wards on patient's specimens and test results;<br>More regular tailored updates on local AMR data to support doctors in empiric treatment;	Training on clinical microbiology for lab staff by locally recognized experts;<br>Review/update SOPs with support from OUCRU lab;<br>Nurse training on collecting clinical specimens;<br>Support from OUCRU lab to review the current microbiology practices and planning for improvement;<br>Analysis of WHONET data twice yearly and creating readable summary of AMR patterns to doctors;
Infection management	S: Hospital-wide consultations with ID doctor available; Head of ID is in the AMS committee<br>W: Limited access to training<br>O: Established connection with HCMC Hospital for Tropical Diseases	Update knowledge and skills in management of specific and locally relevant infectious diseases, clinical pharmacology, and antibiotic treatment guidelines;	Training on infectious diseases management and antibiotic treatment for main clinical syndromes, principals of clinical pharmacology and microbiology, and surgical prophylaxis.

For peer review only - http://bmjopen.bmj.com/site/about/guidelines.xhtml

Component	SWOT analysis (Strength – Weakness – Opportunity – Threat)	Assessment of needs	Proposed interventions
	T: Disease outbreaks (e.g. COVID-19)		
**Hospital 2**			
Leadership	S: Leadership commitment;<br>W: No AMS experience, large committee with no dedicated AMS coordinator for day-to-day activities;<br>O: MoH guidelines and experience from other higher-level hospitals; Leadership dedication to high-quality hospital performance<br>T: Disease outbreaks (e.g. COVID-19) shifting management priorities; Hospital's focus on surgical services and cancer treatment	Knowledge and skills in planning and implementing AMS;<br>Identify clear roles in planning and implementing AMS activities;<br>Raise the awareness and stimulate interests of surgical doctors in AMS<br>Identify targets for interventions;	Establishing AMS action team for day-to-day activities with active coordination from Planning Department and International Relations Department;<br>Select four surgical wards to participate in the study (2 intervention and 2 control wards)<br>Training for AMS team by international & local experts;<br>Collecting and reviewing data to inform interventions
Pharmacy	S: Drug inventories and bidding; Young pharmacists available; Hospital has interest in improving clinical pharmacy program;<br>W: Limited clinical experience; only tracking drug purchasing but not actual drug use data; lack of analytical skills<br>O: Electronic data on patient-level drug administration available from clinical database; Government policy and guidelines (clinical pharmacy, AMS guidelines); Close connection with the Hanoi University of Pharmacy for clinical pharmacy training;<br>T: Non-compliance from surgical departments; bidding process and insurance limits (can affect drug choices and prescribing behaviors)	Identify staff for clinical pharmacist roles;<br>Build clinical pharmacy capacity and data analysis skills	Training for clinical pharmacists in antibiotic treatment, microbiology, pharmacology, and review of antibiotic prescriptions (class-room, hands-on);<br>Implemented prospective audit and feedback as a routine activity of clinical pharmacy program;<br>Participation of clinical pharmacists in clinical ward monthly meeting for which they had to analyze audit data and present to the doctors;<br>Join doctors in their clinical ward rounds to get more clinical experience and interactions with doctors;<br>Support from DASON on using web-based data visualization and benchmarking tool;
Microbiology	S: In-house good-quality microbiology lab; technical support by OUCRU and US CDC; use WHONET database<br>W: Limited clinical interactions;<br>O: Close connection with OUCRU lab; have access to advanced techniques and skills;<br>T: Lack of compliance in sample collection from some clinical wards;	Increase communication with doctors on patient's specimens and test results;<br>Increase clinical experience for clinical microbiologists;<br>More regular tailored updates on AMR data to support doctors in empiric treatment;	Experience sharing and discussions for lab staff with locally recognized experts;<br>Analysis of WHONET data twice yearly and creating readable summary of AMR patterns to doctors;<br>Join doctors in their clinical ward rounds to get more experience and interactions with doctors;
Infection management	S: Hospital-wide consultations with ID doctor and specific specialists available; Strong experience from ICU doctors in difficult cases;<br>W: No presence of ID doctor in AMS committee;<br>O: Established connection with national-level hospitals<br>T: Disease outbreaks (e.g. COVID-19); Dominance of surgical and ICU doctors in determining antibiotic choices for patients	Update knowledge and skills in management of specific and locally relevant infectious diseases, clinical pharmacology, and antibiotic treatment guidelines;<br>Increase the interests and awareness of surgical doctors in AMS	Training on infectious diseases management and antibiotic treatment for main clinical syndromes, principals of clinical pharmacology and microbiology, and surgical prophylaxis for young doctors.<br>Set the target for reducing use of two or more antibiotics for surgical prophylaxis in traumatology department;

Table 2. Number of patients and proportions of antibiotic use by diagnosis groups at intervention and control wards at 2 hospitals in 2019

Diagnosis	Hospital 1				Hospital 2			
	Intervention wards*		Control wards**		Intervention wards#		Control wards##	
	n	%	n	%	n	%	n	%
**ICD Diagnosis Group**								
Certain conditions originating in the perinatal period	35	45.7%	1292	81.4%	10	30.0%	3	66.7%
Certain infectious and parasitic diseases	4756	47.3%	3712	79.3%	308	77.3%	2131	59.6%
Congenital malformations, deformations and chromosomal abnormalities					19	47.4%	8	75.0%
Diseases of the blood and blood-forming organs and certain disorders involving the immune mechanism	20	60.0%	405	23.2%	41	22.0%	19	47.4%
Diseases of the circulatory system	146	79.5%	1828	36.8%	818	35.5%	223	84.8%
Diseases of the digestive system	2568	87.3%	2673	25.0%	676	71.3%	2451	83.6%
Diseases of the ear and mastoid process	38	81.6%	26	26.9%	510	12.4%	3	33.3%
Diseases of the eye and adnexa	2	100.0%	5	100.0%	7	42.9%	1	0.0%
Diseases of the genitourinary system	852	63.0%	1790	71.5%	253	93.3%	82	86.6%
Diseases of the musculoskeletal system and connective tissue	473	35.3%	213	26.8%	83	25.3%	17	58.8%
Diseases of the nervous system	136	72.8%	115	49.6%	136	25.0%	95	86.3%
Diseases of the respiratory system	2039	82.9%	7366	95.2%	813	93.0%	856	93.3%
Diseases of the skin and subcutaneous tissue	195	87.7%	433	68.4%	35	65.7%	20	90.0%
Endocrine, nutritional and metabolic diseases	203	74.9%	322	25.5%	140	30.0%	39	69.2%
External causes of morbidity and mortality	13	69.2%	302	35.4%	5	20.0%	41	70.7%
Factors influencing health status and contact with health services	17	100.0%	3	66.7%	6	16.7%	3	66.7%
Injury, poisoning and certain other consequences of external causes	3504	79.9%	202	40.1%	327	83.5%	120	70.8%
Mental, Behavioral and Neurodevelopmental disorders	3	100.0%	17	52.9%	35	8.6%	8	37.5%
Neoplasms	912	79.3%	409	62.6%	252	61.1%	9186	21.8%
Pregnancy, childbirth and the puerperium	3905	79.3%	4	100.0%	7	71.4%	3	66.7%
Symptoms, signs and abnormal clinical and laboratory findings, not elsewhere classified	224	79.3%	716	76.8%	178	78.1%	673	92.3%
Patients diagnosed with viral causes								
Dengue	1503	9.2%	187	28.9%	28	21.4%	408	21.6%
Influenza	1310	92.1%	4497	87.1%	574	93.0%	673	97.3%
Other viral diagnoses	3213	27.1%	1769	63.4%	169	68.6%	1316	40.7%

n: total number of patients, %: proportion of patients with antibiotic treatment; *include surgical intensive care unit, traumatology, respiratory/ musculoskelectal system, and infectious diseases ward; **include internal intensive care unit, surgical nephro-urology, pediatrics and general internal medicine; #include surgical intensive care unit, general internal medicine, traumatology, and high-quality-serviced ward (provide treatment services with better quality based on patient’s request); ##include internal intensive care unit, oncology, infectious diseases and surgical gastroenterology ward

For peer review only

Figures

Figure 1. Approach to AMS implementation in provincial hospitals and roles of partners

Figure 2. Monthly distribution of antibiotic use in Days of Therapy (DOT) calculated for all patients with antibiotic treatment administered during their admission at the study wards in each hospital per 1000 days present at these wards stratified by study group and antibiotic subgroups

Figure 3. Carbapenem resistance (CR) and multi-drug resistance (MDR) proportions among key bacteria isolated in study wards at two hospitals in 2019. Bacteria include: *Acinetobacter baumannii* (aba) or *Acinetobacter spp.* (ac-), *Escherichia coli* (eco), *Klebsiella pneumoniae* (kpn), or *Klebsiella spp.* (kl-), and *Pseudomonas aeruginosa* (pae).

For peer review only

For peer review only - http://bmjopen.bmj.com/site/about/guidelines.xhtml

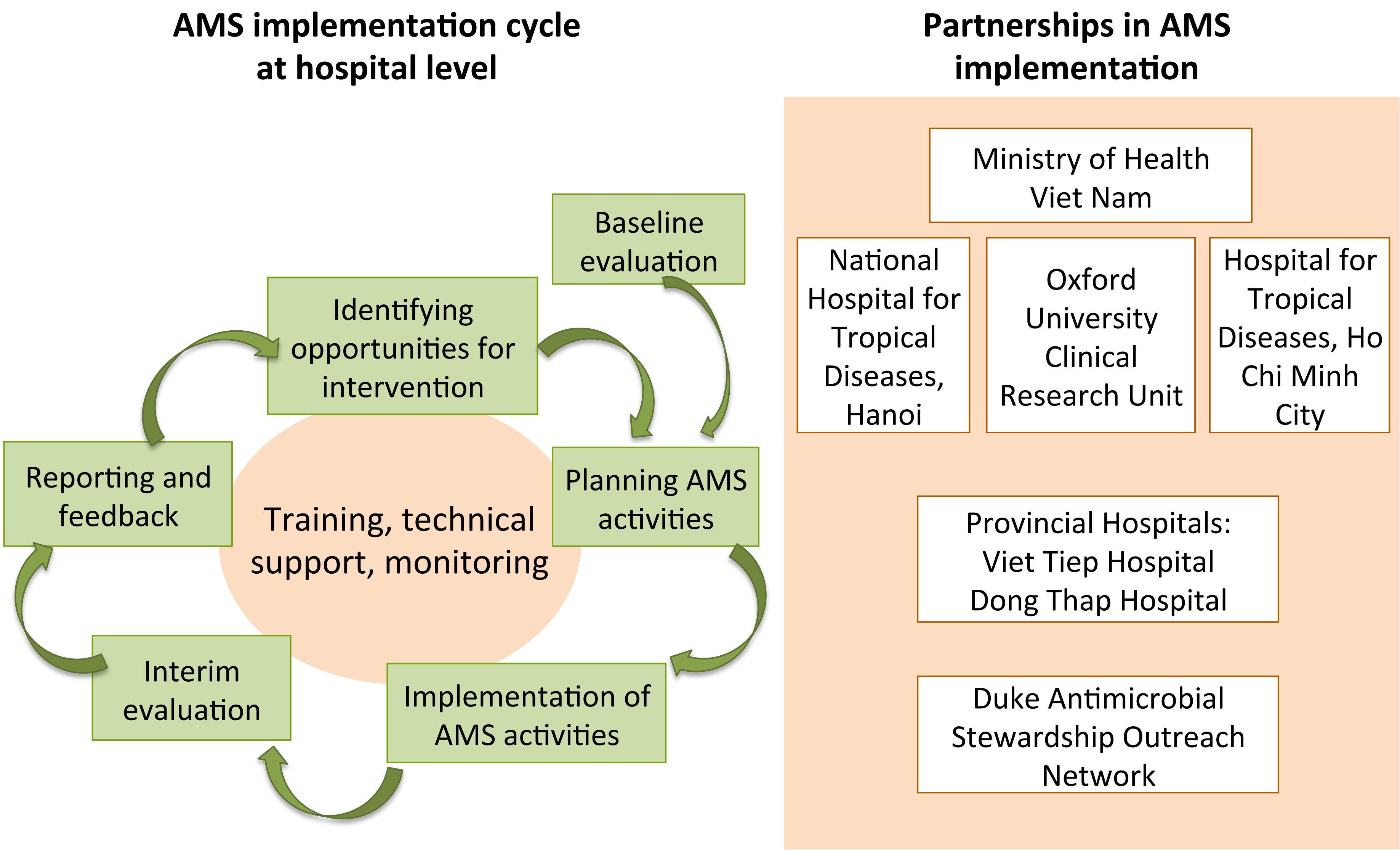
AMS implementation cycle at hospital level
Baseline evaluation
Identifying opportunities for intervention
Planning AMS activities
Implementation of AMS activities
Interim evaluation
Reporting and feedback
Training, technical support, monitoring
Partnerships in AMS implementation
Ministry of Health Viet Nam
National Hospital for Tropical Diseases, Hanoi
Oxford University Clinical Research Unit
Hospital for Tropical Diseases, Ho Chi Minh City
Provincial Hospitals: Viet Tiep Hospital Dong Thap Hospital
Duke Antimicrobial Stewardship Outreach Network

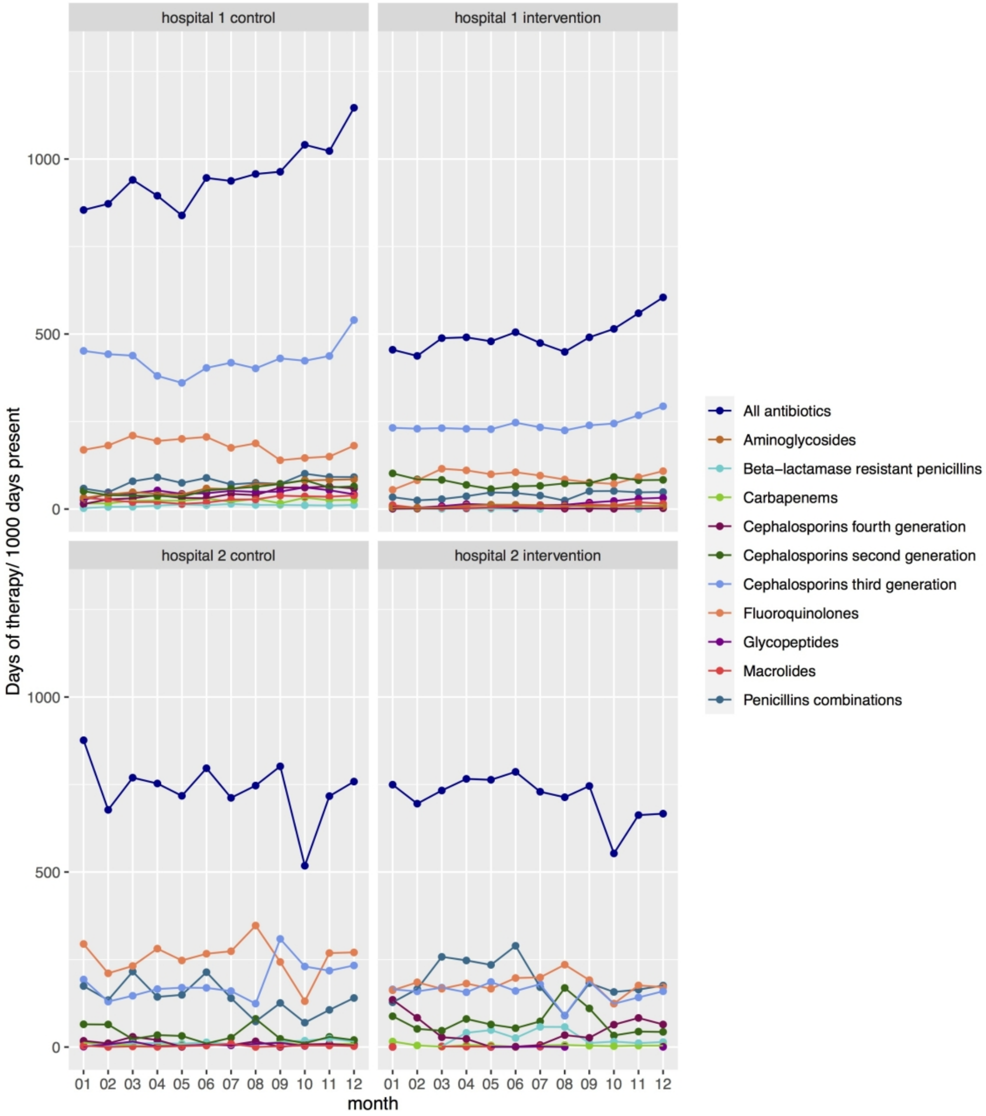

Figure 2. Monthly distribution of antibiotic use in Days of Therapy (DOT) calculated for all patients with antibiotic treatment administered during their admission at the study wards in each hospital per 1000 days present at these wards stratified by study group and antibiotic subgroups

210x238mm (300 x 300 DPI)

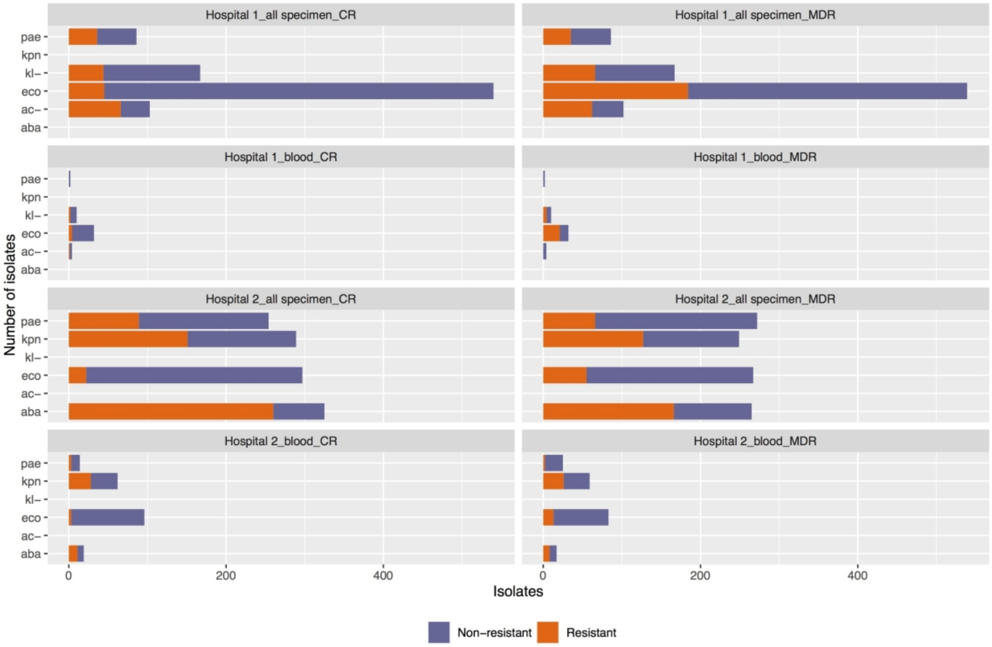

Figure 3. Carbapenem resistance (CR) and multi-drug resistance (MDR) proportions among key bacteria isolated in study wards at two hospitals in 2019. Bacteria include: Acinetobacter baumannii (aba) or Acinetobacter spp. (ac-), Escherichia coli (eco), Klebsiella pneumoniae (kpn), or Klebsiella spp. (kl-), and Pseudomonas aeruginosa (pae).

265x175mm (300 x 300 DPI)

Supplemental Material

Manuscript title: Establishing antimicrobial stewardship programs in 2 provincial-level hospitals in Viet Nam through an implementation research approach

For peer review only

Supplementary Table 1. Study outcome measures and types of data collection

Outcome	Population/ sub-group	Type of data collection
Primary outcome		
Amount of targeted agent groups and total antibiotic use	Intervention and control wards	Extraction of routine data from hospital information systems for 12 months before and 12 months after the start of intervention period
Secondary outcomes		
Changes in knowledge and perceptions of doctors before and after intervention	Intervention wards and control wards	Pre and post-intervention knowledge-attitude-practice (KAP) survey among doctors and pharmacists every 3 months
Proportions of antibiotic prescriptions which:<br>- had an indication documented<br>- had review/stop date documented<br>- were used as surgical prophylaxis for greater than 24 hours<br>- were not compliant with guidelines currently endorsed at the hospital<br>- were inappropriate	Intervention wards	Retrospective medical chart review of 200 patients receiving antibiotics in 4 intervention wards (pre and post-intervention every 3 months)
Proportion of resistant isolates for priority antibiotic-organism combinations	Intervention and control wards	WHONET data – 12 months before and 12 months after intervention
Cost of antibiotic treatment and hospitalizations<br>In-hospital mortality<br>Hospital length of stay	Intervention and control departments	Extraction of routine data from hospital information systems for 12 months before and 12 months after the start of intervention period

*categorized as suboptimal (there may be a mild or non-life-threatening allergy mismatch; or antibiotic prescription is an unreasonable choice for the likely causative or cultured pathogens) or inadequate (antibiotic prescription is unlikely to treat the likely causative or cultured pathogens; or documented or presumed indication does not require any antibiotic treatment; there may be a severe or possibly life-threatening allergy mismatch, or the potential risk of toxicity due to drug interaction; for surgical prophylaxis, the duration is greater than 24 hours (except where local guidelines endorse this) (Source: National Antimicrobial Prescribing Survey (NAPS) - Australia; https://www.naps.org.au/Default.asp)

For peer review only

Supplementary Table 2. Assessment of criteria for clinical microbiology data reported in the study using the Microbiology Investigation Criteria for Reporting Objectively (MICRO) framework

**Item**	**Number**	**Recommendation**	**Assessment of microbiology data reported in this manuscript**
Methods			
Study design	1*	Specimen types: Describe the types of specimen included, i.e. clinical (e.g. blood cultures) or non-diagnostic surveillance (e.g. admission and other screening swabs to diagnose carriage). If specimens were obtained for diagnostic reasons, clinical syndromes should be described where possible, and specimens/isolates stratified by clinical syndrome.	We used data from microbiology laboratory on the antimicrobial susceptibility testing results (from WHONET software) of routine clinical specimens collected for diagnostic reasons from any patients admitted to the hospital. Data were not recorded on the syndromes of patients tested. Screening samples for carriage is not common practice in these hospitals, so one should assume that all samples have been taken for diagnostic purposes.
	2*	Sampling period: State the collection timeframe for specimens yielding isolates for which data is reported, e.g. from MM/YY to MM/YY to be able to identify variability between seasons.	Data reported in this manuscript were for patients admitted to the hospitals in the year 2019 (01/JAN/2019 to 31/DEC/2019).
	3*	Sampling strategy: Describe the strategy for specimen collection, e.g. asymptomatic screening, sampling of all febrile patients, sampling at clinician discretion, sampling of specific patient groups and convenience sampling (e.g. use of isolates from an existing sample repository). Specify whether sampling followed routine clinical practice or was protocol driven. Classify specimens as from community-acquired (CAI) or hospital-acquired (HAI) infections. The definition of HAI used (e.g. HAI defined by specimen collection > 48 h after hospital admission) should be provided and should use ideally an international standard (e.g. US Centers for Disease Control).	Data were from routine sampling of specimens from any patients admitted at the hospital at clinician discretion. The data didn't allow for classification in HAI vs CAI.
	4	Target organisms: Explicitly state which organisms/organism groups were included in the report. Nomenclature should follow international standards (i.e. using approved genus/species names as summarised in the International Journal of Systematic and Evolutionary Microbiology). Lists of approved bacterial names can be downloaded from Prokaryotic Nomenclature Up-to-Date (https://www.dsmz.de/bacterial-diversity/prokaryotic-nomenclature-up-	Data was collected for all organisms identified at the routine microbiology lab of each hospital. The 5 species most frequently isolated were kept in the analysis and the present report: *Escherichia coli*, *Staphylococcus aureus*, *Klebsiella pneumoniae*, *Acinetobacter baumanii*, and *Pseudomonas aeruginosa*.

Item	Number	Recommendation	Assessment of microbiology data reported in this manuscript
		to-date.html) and the List of Prokaryotic Names with Standing in Nomenclature (http://www.bacterio.net/). Organisms considered contaminants should be listed, if appropriate (e.g. coagulase negative staphylococci or *Corynebacterium* spp..	
Setting	5*	Geographical setting: Describe the geographical distribution of specimens/patients from which isolates were obtained, at least to a country level, but preferably to a sub-national level or a geoposition.	Isolates reported were from patients in the catchment area of each hospital (in Hai Phong and Dong Thap province, Viet Nam).
	6*	Clinical setting: Describe the type and level of the healthcare facilities (e.g. primary, secondary, tertiary) from which specimens were obtained. If stating a microbiology laboratory, the centres served by the laboratory should be specified.	Microbiology data were from the routine microbiology laboratory at each of the two provincial hospitals, which are general hospitals in each province in the Viet Nam public healthcare system (consisting of national, provincial and district level hospitals).
Laboratory work	7	Specimen processing: If applicable, describe specimen collection and handling, processing and sub-culture methods for all types of specimen included. For example, if reporting AST results for blood culture and cerebrospinal fluid culture isolates, the processing of these specimens by the laboratory should be briefly explained, including how specimens are sub-cultured, the media used, incubation conditions and duration. A summary of specimen processing steps (e.g. pre-processing steps, nucleic acid extraction method (if applicable), amplification platform, contamination avoidance strategy) should be provided for molecular-only workflows (e.g. to detect *Mycobacterium tuberculosis* and rifampicin resistance using the Cepheid Xpert MTB/RIF system).	Specimen collection and handling, processing and sub-culture methods followed hospital Standard Operating Procedures developed based on the guidelines issued by the Ministry of Health (MoH) in 2017, the subsequent trainings from MoH and OUCRU. Blood culture is performed with biphasic blood culture bottles (Nam Khoa Biotek, Vietnam) in Hospital 1 and with BacT-Alert automate in Hospital 2 (Biomerieux, France). Cultures are performed on homemade media (Oxoid dehydrated culture media, UK) or ready-to-use media (DEKA, Vietnam) for blood agar, chocolate agar, and Mueller-Hinton.
	8*	Target organism identification: Details of identification methodology should be reported briefly. Where identification databases were used (e.g. bioMerieux API/bioMerieux VITEK-MS/Bruker Biotyper), the version should be specified.<br>In general, all pathogens should be identified to species level. In the case of *Salmonella* species, organisms should be identified to at least the *S. Typhi*, *S. Paratyphi*, or non-typhoidal Salmonella (NTS) level. Strain subtyping methods should be reported according to STROME-ID.	Identification was based on the conventional biochemical methods in both hospitals: API galleries (Biomerieux, France) and with manual phenotypic tests in Hospital 1, and with Vitek II (Biomerieux, France; identification database version 9.02) in Hospital 2.

For peer review only

Item	Number	Recommendation	Assessment of microbiology data reported in this manuscript
	9*	Antimicrobial susceptibility testing: Describe the antimicrobial susceptibility testing methods used, internal quality control processes and their interpretation, with reference to a recognised international standard, e.g. CLSI, EUCAST. Where an international standard was followed, the specific edition(s) of guidelines used should be referenced. Deviations from standard methodology should be described, along with evidence of validation. Handling of any changes to interpretative criteria during the sampling period should be documented. State whether the raw AST data (zone diameters and/or minimum inhibitory concentrations) were re-categorised with updated breakpoints or left as-is.	Antimicrobial susceptibility testing methods: disc diffusion at both hospitals; Automated Vitek was also used by Hospital 2;<br>Routine AST data at the microbiology labs was entered into WHONET 5.6 by hospital technicians. Extracted files were converted to WHONET format using BacLink, a free tool included in WHONET. AST results were categorised in S/I/R according to CLSI 2020 guidelines for the present report.
	10	Additional tests performed to identify resistance mechanisms: Describe the testing methods used for adjunctive/confirmatory antimicrobial susceptibility tests, such as enzymatic/molecular assays (e.g. Xpert MTB/RIF, mecA PCR) and inducible resistance assays, with reference to a recognised international standard, where available. Where an international standard was followed, the specific edition of guidelines used should be referenced. Deviations from standard methodology should be described, along with evidence of validation.	Not applicable.
	11*	Antimicrobial resistance definitions: Define resistance for each antimicrobial class (i.e. are isolates in the 'intermediate' category included within 'susceptible' or 'resistant' or analysed as a distinct category). If using the term, define MDR (e.g. ≥ 1 agent in ≥ 3 classes tested). For each organism type, an MDR test panel must be defined, consisting of the minimum panel of individual antimicrobial agents/classes against which an isolate must be tested for that isolate to be considered tested for MDR status. Antimicrobials to which an organism is intrinsically resistant cannot be part of the test panel or contribute to MDR status.	Definitions of resistance to carbapenem and MDR were described in the main methods section of the manuscript based on the criteria proposed by Magiorakos et al [14] as follows:<br>- For *Acinetobacter baumannii* and *Acinetobacter spp.*: MDR is defined as resistant to at least 3 of the following: cephalosporin (ceftriaxone or cefepime), aminoglycosides (amikacin, gentamicin or tobramycin), ciprofloxacin and carbapenem (imipenem or meropenem); carbapenem resistance is defined as resistant to imipenem or meropenem;<br>- For *Escherichia coli, Klebsiella pneumoniae,* and *Klebsiella spp.*: MDR is defined as resistant to at least 3 of the following: carbapenem (ertapenem, imipenem or meropenem), cephalosporin (ceftriaxone or cefepime),

Item	Number	Recommendation	Assessment of microbiology data reported in this manuscript
			aminoglycosides (amikacin, gentamicin or tobramycin) and ciprofloxacin; carbapenem resistance is defined as resistant to ertapenem, imipenem, or meropenem;<br>- For *Pseudomonas aeruginosa*: MDR is defined as resistant to at least 3 of the following 4 agents: imipenem, ceftazidime, ciprofloxacin and tobramycin; carbapenem resistance is resistant to imipenem or meropenem;
Quality assurance	12*	External quality assurance: State whether the microbiology laboratory participates in an external quality control programme and, if so, provide scheme details. Examples include the UK National External Quality Assurance Scheme (www.ukneqasmicro.org.uk) and the American College of Pathologists External Quality Assurance/Proficiency Testing Program (https://www.cap.org/)	Hospital 1: participated in two external quality control programs: UK NEQAS and Ho Chi Minh City quality assurance program<br>Hospital 2: participated in two external quality control programs: UK NEQAS and Hanoi Medical University – Viet Nam
	13	Accreditation: State whether the laboratory is accredited through a national or international body (e.g. the International Standards Organisation, ISO) and specify which assays are covered in the accreditation.	No ISO certification. Hospital 2 has participated in the Strengthening Laboratory Management Toward Accreditation (SLMTA) program.
Bias	14*	Duplicate and sequential isolates: The strategy for accounting for duplicate and sequential isolates from the same patient should be clearly detailed. Duplicate isolates are multiple isolates of the same phenotypic organism (i.e. same species and same resistance profile) from the same patient on the same date cultured either from the same clinical specimen, or from two separate clinical specimens, such as blood and CSF. Sequential isolates are isolates of the same phenotypic organism from the same patient at different dates, such as blood cultures taken on different dates. Various strategies for the handling of duplicate and sequential isolates exist, and the strategy used should be transparent as it will bias pooled resistance results. For example, inclusion of all isolates (the 'all isolate strategy') has been shown to shift pooled resistance proportions toward greater resistance, whilst inclusion of only the first isolate per patient (the 'first isolate strategy') or only the first isolate per	WHONET data were de-duplicated, one isolate representing one patient. Only the first isolate per patient, per pathogen, per reporting period (i.e. for the whole year 2019) at each hospital was included.

For peer review only

Item	Number	Recommendation	Assessment of microbiology data reported in this manuscript
		infection episode (the ‘episode-based strategy’) will shift pooled results toward susceptibility.	
Results			
	15*	Population: Describe the demographics of the population from which clinical specimens and subsequent isolates have been obtained, disaggregating age and gender data.	Population: patients admitted to each hospital in one calendar year (2019) and stratified by clinical wards. Data on the isolates were described with age and gender information in the manuscript.
	16*	Denominators: Patient and isolate denominators should be used appropriately to ensure clarity regarding the numbers included in each analysis. Of particular importance is the reporting of resistance where first- and second-line AST panels were used (i.e. not all isolates of a particular species were tested against all agents). For drugs where only a subset of isolates were tested, reporting of a percentage without the numbers of isolates tested/resistant may be highly misleading.	Denominators were presented for each percentage described in the manuscript main text. No second-line AST is used in either one of the hospitals.
	17	Site/place of acquisition: AST data from CAI and HAI should be reported and analysed separately.	Not reported from the microbiology data from lab.
	18*	Reporting resistance proportions for single agent and class resistance: Proportions of resistant isolates should be reported as number of isolates susceptible or resistant to a given antimicrobial agent/class out of actual number of isolates tested for susceptibility to that agent/class.	The manuscript focuses on specific types of resistance for each pathogen and reported the proportions of resistant isolates out of the actual number of isolates tested for susceptibility to the specific agents under each type.
	19	Reporting multidrug resistance proportions: If defined, the proportion of MDR isolates should be expressed as the number of MDR isolates out of the number of isolates tested (i.e. the number undergoing the MDR test panel specific to that organism). Single agent/class resistance should be always be reported, regardless of MDR reporting.	As criterion 18. We reported proportions of resistance to single agent or class in Supplementary Table 4.
Discussion			
	20	Discuss any reasons why bias may have been introduced into the reported	We reported the baseline AST data of the study wards in each

For peer review only

Item	Number	Recommendation	Assessment of microbiology data reported in this manuscript
Limitations		data, due to patient/specimen selection, isolation of organisms, or otherwise. Consider factors which may have either introduced bias into the types of organisms isolated or the antimicrobial susceptibility profiles, e.g. receipt of antimicrobials prior to specimen collection will reduce the yield of certain species and also select for more resistant organisms.	hospitals using the routine data collected for diagnostic reasons. No diagnostic stewardship program existed at baseline in either one of the 2 hospitals. Therefore, it is expected that the clinicians' testing habits have impacted the selection of samples and isolates subsequently tested for antimicrobial susceptibility. Because reimbursement of laboratory test is based on strict clinical criteria, the isolates may not represent the population of pathogens, but this bias remains constant between wards, between hospitals, and with time.

*Core 'must include' items

For peer review only

Supplementary Table 3. Summary of National AMS Stakeholder Meeting in March 2019

**Participating stakeholders**	Medical Services Administration (MSA) Viet Nam<br>Vietnamese Pharmaceutical Association<br>Viet Nam Centers for Disease Control and Prevention<br>Hanoi Medical University, Hanoi<br>Hanoi University of Pharmacy, Hanoi<br>Drugs Administration Viet Nam, MoH<br>MSD Pharmaceuticals<br>National Hospital for Tropical Diseases, Hanoi<br>Hospital for Tropical Diseases, Ho Chi Minh City<br>Viet Nam National Children Hospital, Hanoi<br>Cho Ray Hospital, Ho Chi Minh city<br>Uong Bi Viet Nam – Sweden Hospital, Quang Ninh<br>Can Tho General Hospital<br>Da Nang General Hospital<br>Hue General Hospital<br>Children's Hospital 1, Ho Chi Minh City<br>Hoan My Private Hospital, Viet Nam<br>World Health Organization (WHO) – Viet Nam<br>Oxford University Clinical Research Unit (OUCRU)<br>US Centers for Disease Control and Prevention – Viet Nam<br>Woolcock Viet Nam<br>PATH Viet Nam
**Key presentations and discussions**	MSA/ WHO implementation of antimicrobial stewardship in hospitals: review of current state and hospital survey;<br>AMS implementation experience of Cho Ray Hospital, Hue Central Hospital, National Hospital for Tropical Diseases, and Hoan My Hospital;<br>MSD program on antimicrobial stewardship in Viet Nam: a perspective from the pharmaceutical company;<br>OUCRU qualitative study on AMS implementation in seven hospitals in the AMR surveillance network;<br>Group discussions on the challenges faced in AMS and proposed solutions to improve the implementation;<br>WHO Coordination and Finding the Way Forward.
**Main areas for actions identified**	1. Strengthening a supportive policy environment for AMS: With the National Action Plan to combat AMR and subsequent guidelines, the legal framework has been set up and shows the strong commitment of the government in AMS implementation in Viet Nam. However, national guidelines

For peer review only

	clearly need revisions and updates to fit in the different contexts where AMS work is required, particularly in small and resources-limited healthcare settings.<br>2. Developing AMS training curriculum and core AMS related competencies: Training modules on AMS should be standardized and formalized into the undergraduate and Continuing Professional Development programs and available in a variety of formats including online courses. These should also be available to any healthcare professionals taking on AMS roles in hospital and other settings.<br>3. Establishing coordination and collaboration in AMS activities nationally and regionally: Coordination of activities across the country and in each region is crucial to achieve a synergy of efforts and minimize duplications and wastes of resources. Smaller hospitals can learn from and utilize resources available from larger hospitals in the same region. Hospitals with experience in implementing AMS programs and those having been identified as centers of excellence can share experience with and support other hospitals in this process.

For peer review only

Supplementary Table 4. Baseline proportions of resistance for common pathogens to each agent/class under evaluation

**Resistant / Tested (%)**	***E. coli***		***K. pneumoniae**** **or *Klebsiella spp.*****		***A. baumannii**** **or *Acinetobacter spp.*****		***P. aeruginosa***		***S. aureus***	
	All specimens	Blood	All specimens	Blood	All specimens	Blood	All specimens	Blood	All specimens	Blood
**Hospital 1**										
Carbapenem	45/540 (8.3)	4/32 (12.5)	44/167 (26.3)	2/10 (20.0)	66/103 (64.1)	1/4 (25.0)	36/86 (41.9)	0/2 (0.0)	-	-
Cephalosporin	360/540 (66.7)	27/32 (84.4)	95/167 (56.9)	6/10 (60.0)	87/103 (84.5)	1/4 (25.0)	34/86 (39.5)	0/2 (0.0)	-	-
Aminoglycoside	245/540 (45.4)	21/32 (65.6)	71/167 (42.5)	5/10 (50.0)	86/103 (83.5)	2/4 (50.0)	47/86 (54.7)	1/2 (50.0)	115/203 (56.7)	25/40 (62.5)
Ciprofloxacin	420/539 (77.9)	29/32 (90.6)	111/167 (66.5)	5/10 (50.0)	85/102 (83.3)	1/4 (25.0)	45/86 (52.3)	0/2 (0.0)	94/210 (44.8)	26/42 (61.9)
Ceftazidime	288/540 (53.3)	24/32 (75.0)	98/167 (58.7)	4/10 (40.0)	79/103 (76.7)	1/4 (25.0)	35/85 (41.2)	0/2 (0.0)	-	-
Cefoxitin	163/540 (30.2)	12/32 (37.5)	72/167 (43.1)	3/10 (30.0)	-	-	-	-	166/210 (79.0)	-
MDR	184/539 (34.1)	21/32 (65.6)	66/167 (39.5)	4/10 (40.0)	62/102 (60.8)	0/4 (0.0)	35/86 (40.7)	0/2 (0.0)		
**Hospital 2**										
Carbapenem	22/297 (7.4)	3/96 (3.1)	151/289 (52.2)	28/62 (45.2)	260/325 (80.0)	11/19 (57.9)	89/254 (35.0)	3/14 (21.4)	-	-
Cephalosporin	69/273 (25.3)	17/83 (20.5)	141/256 (55.1)	28/61 (45.9)	226/269 (84.0)	11/17 (64.7)	41/175 (23.4)	2/11 (18.2)	-	-
Aminoglycoside	137/297 (46.1)	39/96 (40.6)	149/288 (51.7)	32/62 (51.6)	229/326 (70.2)	11/19 (57.9)	137/254 (53.9)	6/14 (42.9)	79/178 (44.4)	14/32 (46.9)
Ciprofloxacin	197/275 (71.6)	58/88 (65.9)	188/253 (74.3)	36/60 (60.0)	252/306 (82.3)	11/19 (57.9)	124/207 (59.9)	6/12 (50.0)	71/170 (41.8)	11/31 (35.5)
Ceftazidime	121/295 (41.1)	31/95 (32.6)	182/288 (63.2)	35/62 (56.5)	273/323 (84.5)	12/19 (63.2)	64/253 (25.3)	2/14 (14.3)	-	-
Cefoxitin	17/55 (30.9)	8/23 (34.8)	29/60 (48.3)	3/5 (60.0)	-	-	-	-	79/103 (76.7)	8/17 (47.1)
MDR	55/267 (20.6)	13/83 (15.7)	127/249 (51.0)	26/59 (44.1)	166/265 (62.6)	8/17 (47.1)	66/272 (24.3)	2/25 (8.0)	-	-

* in Hospital 2; ** in Hospital 1. Cephalosporin: ceftriaxone or cefepime; Aminoglycoside: amikacin, gentamicin, or tobramycin; Carbapenem: imipenem or meropenem (for *A. baumannii*, *Acinetobacter spp.*, *P. aeruginosa*); Carbapenem: ertapenem, imipenem, or meropenem (for *E. coli, K. pneumoniae, Klebsiella spp.*).
MDR for *A. baumannii* and *Acinetobacter spp.*: resistant to at least 3 of the following: cephalosporin (ceftriaxone or cefepime), aminoglycosides (amikacin, gentamicin or tobramycin), ciprofloxacin and carbapenem (imipenem or meropenem). MDR for *E. coli, K. pneumoniae, and Klebsiella spp.*: resistant to at least 3 of the following: carbapenem (ertapenem, imipenem or meropenem), cephalosporin (ceftriaxone or cefepime), aminoglycosides (amikacin, gentamicin or tobramycin) and ciprofloxacin; MDR for *P. aeruginosa*: resistant to at least 3 of the following 4 agents: imipenem, ceftazidime, ciprofloxacin and tobramycin.

For peer review only

Standards for Reporting Implementation Studies: the StaRI checklist for completion

Notes: A key concept of the StaRI standards is the dual strands of describing, on the one hand, the implementation strategy and, on the other, the clinical, healthcare, or public health intervention that is being implemented. These strands are represented as two columns in the checklist.

The primary focus of implementation science is the implementation strategy (column 1) and the expectation is that this will always be completed.

The evidence about the impact of the intervention on the targeted population should always be considered (column 2) and either health outcomes reported or robust evidence cited to support a known beneficial effect of the intervention on the health of individuals or populations.

The StaRI standardsrefers to the broad range of study designs employed in implementation science. Authors should refer to other reporting standards for advice on reporting specific methodological features. Conversely, whilst all items are worthy of consideration, not all items will be applicable to, or feasible within every study.

Checklist item		Reported on page #	Implementation Strategy	Reported on page #	Intervention
			"Implementation strategy" refers to how the intervention was implemented		"Intervention" refers to the healthcare or public health intervention that is being implemented.
**Title and abstract**					
Title	**1**	1	Identification as an implementation study, and description of the methodology in the title and/or keywords		
Abstract	**2**	2	Identification as an implementation study, including a description of the implementation strategy to be tested, the evidence-based intervention being implemented, and defining the key implementation and health outcomes.		
**Introduction**					
Introduction	**3**	4-5	Description of the problem, challenge or deficiency in healthcare or public health that the intervention being implemented aims to address.		
Rationale	**4**	6-7	The scientific background and rationale for the implementation strategy (including any underpinning theory/framework/model, how it is expected to achieve its effects and any pilot work).	4	The scientific background and rationale for the intervention being implemented (including evidence about its effectiveness and how it is expected to achieve its effects).
Aims and objectives	**5**	5	The aims of the study, differentiating between implementation objectives and any intervention objectives.		
**Methods: description**					
Design	**6**	5-8	The design and key features of the evaluation, (cross referencing to any appropriate methodology reporting standards) and any changes to study protocol, with reasons		

For peer review only - http://bmjopen.bmj.com/site/about/guidelines.xhtml

Context	**7**	5	The context in which the intervention was implemented. (Consider social, economic, policy, healthcare, organisational barriers and facilitators that might influence implementation elsewhere).		
Targeted 'sites'	**8**	5-6	The characteristics of the targeted 'site(s)' (e.g locations/personnel/resources etc.) for implementation and any eligibility criteria.	6	The population targeted by the intervention and any eligibility criteria.
Description	**9**	5-8	A description of the implementation strategy	10-11	A description of the intervention
Sub-groups	**10**	6	Any sub-groups recruited for additional research tasks, and/or nested studies are described		
**Methods: evaluation**					
Outcomes	**11**	7	Defined pre-specified primary and other outcome(s) of the implementation strategy, and how they were assessed. Document any pre-determined targets	7	Defined pre-specified primary and other outcome(s) of the intervention (if assessed), and how they were assessed. Document any pre-determined targets
Process evaluation	**12**	7-8	Process evaluation objectives and outcomes related to the mechanism by which the strategy is expected to work		
Economic evaluation	**13**	*	Methods for resource use, costs, economic outcomes and analysis for the implementation strategy	*	Methods for resource use, costs, economic outcomes and analysis for the intervention
Sample size	**14**	6**	Rationale for sample sizes (including sample size calculations, budgetary constraints, practical considerations, data saturation, as appropriate)		
Analysis	**15**	8-9	Methods of analysis (with reasons for that choice)		
Sub-group analyses	**16**	8-9	Any a priori sub-group analyses (e.g. between different sites in a multicentre study, different clinical or demographic populations), and sub-groups recruited to specific nested research tasks		

For peer review only - http://bmjopen.bmj.com/site/about/guidelines.xhtml

**Results**					
Characteristics	**17**	11-14	Proportion recruited and characteristics of the recipient population for the implementation strategy	NA	Proportion recruited and characteristics (if appropriate) of the recipient population for the intervention
Outcomes	**18**	12-14#	Primary and other outcome(s) of the implementation strategy	NA##	Primary and other outcome(s) of the Intervention (if assessed)
Process outcomes	**19**	10-12	Process data related to the implementation strategy mapped to the mechanism by which the strategy is expected to work		
Economic evaluation	**20**	*	Resource use, costs, economic outcomes and analysis for the implementation strategy	*	Resource use, costs, economic outcomes and analysis for the intervention
Sub-group analyses	**21**	NA##	Representativeness and outcomes of subgroups including those recruited to specific research tasks		
Fidelity/ adaptation	**22**	10-12	Fidelity to implementation strategy as planned and adaptation to suit context and preferences	NA	Fidelity to delivering the core components of intervention (where measured)
Contextual changes	**23**	10-12	Contextual changes (if any) which may have affected outcomes		
Harms	**24**	NA##	All important harms or unintended effects in each group		
**Discussion**					
Structured discussion	**25**	14-17	Summary of findings, strengths and limitations, comparisons with other studies, conclusions and implications		
Implications	**26**	18	Discussion of policy, practice and/or research implications of the implementation strategy (specifically including scalability)	18	Discussion of policy, practice and/or research implications of the intervention (specifically including sustainability)
**General**					
Statements	**27**	9-10	Include statement(s) on regulatory approvals (including, as appropriate, ethical approval, confidential use of routine data, governance approval), trial/study registration (availability of protocol), funding and conflicts of interest		

* We mentioned about recording the economic indicators, however methods and analysis will be done and reported separately; the costing methodology employed will be the time-driven activity based costing approach. ** We selected 4 intervention wards and 4 control wards based on the funding availability. #We presented in this manuscript the outcomes of our implementation strategy that has

For peer review only

a description of the process in setting up the program at each hospital and baseline data for outcome indicators of antibiotic use and resistance. ##These will be described at the end of the intervention period in the next report.

For peer review only

BMJ Open

Assessing feasibility of establishing antimicrobial stewardship programs in two provincial-level hospitals in Viet Nam; an implementation research study

Journal:	*BMJ Open*
Manuscript ID	bmjopen-2021-053343.R1
Article Type:	Original research
Date Submitted by the Author:	23-Aug-2021
Complete List of Authors:	Vu, Huong; Oxford University Clinical Research Unit, Ha Noi Unit<br>Ta, Thi Dieu Ngan; National Hospital of Tropical Diseases<br>Huynh, Phuong Thao; Hospital for Tropical Diseases<br>Le, Minh Quang; Viet Tiep Hospital<br>Tran, Thi Thu Hanh; Viet Tiep Hospital<br>Nguyen, Thi Hien; Viet Tiep Hospital<br>Tran, Duc; Viet Tiep Hospital<br>Vu, Hai Vinh; Viet Tiep Hospital<br>Chau, Minh Duc; Dong Thap Hospital<br>Vo, Thi Hoang Dung Em; Dong Thap Hospital<br>Phan, Van Be Bay; Dong Thap Hospital<br>Nguyen, Thi Thuy Oanh; Dong Thap Hospital<br>Pham Thi Thuy, Hang; Dong Thap Hospital<br>Nguyen, Cam Tu; Oxford University Clinical Research Unit<br>Truong, Anh Quan; Oxford University Clinical Research Unit, Ha Noi Unit<br>Kesteman, Thomas; Oxford University Clinical Research Unit, Ha Noi Unit<br>Dodds Ashley, Elizabeth; Duke University<br>Anderson, D; Duke University<br>van Doorn, H. Rogier; Oxford University Clinical Research Unit; Nuffield Department of Clinical Medicine
**Primary Subject Heading**:	Infectious diseases
Secondary Subject Heading:	Evidence based practice, Global health, Health policy, Public health
Keywords:	Health & safety < HEALTH SERVICES ADMINISTRATION & MANAGEMENT, Health policy < HEALTH SERVICES ADMINISTRATION & MANAGEMENT, Quality in health care < HEALTH SERVICES ADMINISTRATION & MANAGEMENT, PUBLIC HEALTH, HEALTH SERVICES ADMINISTRATION & MANAGEMENT, INTERNAL MEDICINE

SCHOLARONE™
Manuscripts

I, the Submitting Author has the right to grant and does grant on behalf of all authors of the Work (as defined in the below author licence), an exclusive licence and/or a non-exclusive licence for contributions from authors who are: i) UK Crown employees; ii) where BMJ has agreed a CC-BY licence shall apply, and/or iii) in accordance with the terms applicable for US Federal Government officers or employees acting as part of their official duties; on a worldwide, perpetual, irrevocable, royalty-free basis to BMJ Publishing Group Ltd ("BMJ") its licensees and where the relevant Journal is co-owned by BMJ to the co-owners of the Journal, to publish the Work in this journal and any other BMJ products and to exploit all rights, as set out in our licence.

The Submitting Author accepts and understands that any supply made under these terms is made by BMJ to the Submitting Author unless you are acting as an employee on behalf of your employer or a postgraduate student of an affiliated institution which is paying any applicable article publishing charge ("APC") for Open Access articles. Where the Submitting Author wishes to make the Work available on an Open Access basis (and intends to pay the relevant APC), the terms of reuse of such Open Access shall be governed by a Creative Commons licence – details of these licences and which Creative Commons licence will apply to this Work are set out in our licence referred to above.

Other than as permitted in any relevant BMJ Author's Self Archiving Policies, I confirm this Work has not been accepted for publication elsewhere, is not being considered for publication elsewhere and does not duplicate material already published. I confirm all authors consent to publication of this Work and authorise the granting of this licence.

For peer review only

Manuscript title: Assessing feasibility of establishing antimicrobial stewardship programs in two provincial-level hospitals in Viet Nam; an implementation research study

Authors: Vu Thi Lan Huong[1], Ta Thi Dieu Ngan[2], Huynh Phuong Thao[3], Le Minh Quang[4], Tran Thi Thu Hanh[4], Nguyen Thi Hien[4], Tran Duc[4], Vu Hai Vinh[4], Chau Minh Duc[5], Vo Thi Hoang Dung Em[5], Phan Van Be Bay[5], Nguyen Thi Thuy Oanh[5], Pham Thi Thuy Hang[5], Nguyen Thi Cam Tu[1], Truong Anh Quan[1], Thomas Kesteman[1], Elizabeth Dodds Ashley[6], Deverick J. Anderson[6], H Rogier van Doorn[1,7]

[1]Oxford University Clinical Research Unit, 78 Giai Phong, Hanoi, Viet Nam
[2]National Hospital for Tropical Diseases, 78 Giai Phong, Hanoi, Viet Nam
[3]Hospital for Tropical Diseases, 764 Vo Van Kiet, Ho Chi Minh City, Viet Nam
[4]Viet Tiep Hospital, 1 Nha Thuong, Cat Dai, Le Chan, Hai Phong, Viet Nam
[5]Dong Thap Hospital, 144 Mai Van Khai, My Tan, Cao Lanh City, Đong Thap
[6] Duke Antimicrobial Stewardship Outreach Network, Duke Center for Antimicrobial Stewardship and Infection Prevention, Duke University, Durham, NC 27710, United States
[7]Centre for Tropical Medicine and Global Health, Nuffield Department of Medicine, University of Oxford, Oxford, UK

Correspondence to Vu Thi Lan Huong; huongvtl@oucru.org

For peer review only - http://bmjopen.bmj.com/site/about/guidelines.xhtml

ABSTRACT

Objectives: To investigate the feasibility of establishing hospital-based antimicrobial stewardship (AMS) programs comprising action-planning, educational interventions and data feedback in two provincial-level hospitals in Viet Nam.

Design and setting: This was an implementation research using participatory action process and existing resources from the Duke Antimicrobial Stewardship Outreach Network (DASON) with local adjustments. A national stakeholder meeting and Strengths-Weaknesses-Opportunities-Threats (SWOT) analysis were conducted to identify gaps and potential interventions.

Participants: Hospital AMS staff implemented activities throughout the study phases. Routinely collected patient data were analyzed to support planning, implementation and evaluation.

Interventions: Hospitals were considered as a complex adaptive system and leveraged their unique characteristics and interconnections to develop one-year plans containing core interventions (data use, educational training, prospective audit with feedback (PAF), and evaluations).

Outcome measures: We assessed feasibility using outputs from stakeholder meeting, SWOT analysis, baseline data, planning process and implementation.

Results: The stakeholder meeting identified three gaps for AMS at national level: supportive policies, AMS training and core competencies, and collaboration. At the hospitals, AMS programs took one year for planning due to lack of hospital-specific procedures and relevant staff competencies. Baseline data (January-December 2019) showed variations in antibiotic consumption: 951 days of therapy per 1000 days present in the control and 496 in the intervention wards in Hospital 1, and 737 and 714 in Hospital 2 respectively. During one-year implementation, clinical pharmacists audited 1890 antibiotic prescriptions in Hospital 1 (June 2020-May 2021) and 1628 in Hospital 2 (July 2020-July 2021), and will continue PAF in their daily work.

Conclusion: Our data confirmed the need to contextualize AMS programs in LMICs and demonstrated the usefulness of implementation research design in assessing program feasibility. Developing staff competencies, using local data to stimulate actions, and integrating program activities in routine hospital work are key to success in LMICs.

For peer review only

For peer review only - http://bmjopen.bmj.com/site/about/guidelines.xhtml

Key words: Antimicrobial stewardship, low- and middle-income countries, Viet Nam, implementation research, action planning

Strengths and limitations of the study

- Our study used a participatory and teamwork approach involving hospitals as a complex adaptive system with unique characteristics and interconnections that can be leveraged to develop locally tailored interventions.
- We employed intervention strategies that were conducted using locally available staff and resources, specifically clinical pharmacists and routinely collected data.
- In the process of setting up AMS programs, we incorporated perspectives and expertise from both policy makers and hospitals at different levels and from international and local partners.
- Contextualization and tailoring is a strength but this also affects the study's external validity, and the results cannot be generalized to all healthcare settings as this implementation study targeted community hospitals with medium levels of resources and staff available.

For peer review only

INTRODUCTION

Despite fast-growing literature on the effectiveness and potential economic impact of antimicrobial stewardship (AMS) in healthcare facilities around the world [1-5], limited data from hospitals in low- and middle-income countries (LMICs) show numerous challenges in these settings and that implementation must be tailored to the local contexts to have an effect on antibiotic use and antimicrobial resistance (AMR).[6 7] Viet Nam, like many other LMICs, is affected by high and increasing levels of AMR. Surveillance data from Viet Nam's AMR surveillance network show that the prevalence of antibiotic resistance is among the highest in Asia and the world. [8-10] Hospital-wide antibiotic consumption data from this hospital network also showed critical broad-spectrum and last-resort antibiotics are increasingly used as empiric treatment. In a 12-month point prevalence survey at 15 adult intensive care units within the AMR surveillance network in 2012, carbapenems were the third most commonly used class in all patients and the most common among those with hospital acquired infections.[11]

The Ministry of Health (MoH) in Viet Nam developed a National Action Plan on AMR in 2013 for 2013-2020. Surveillance and antimicrobial stewardship (AMS) programs were two of the six listed priorities of the plans. A nation-wide AMR hospital surveillance network and a reference laboratory have been established.[9 12] A national AMS implementation guideline was issued in 2016 [13] and updated in 2020 [14] which required each hospital to establish a multidisciplinary AMS team and implement a set of recommended activities. However, many hospitals continue to struggle to implement AMS despite implementation guidance from the Viet Nam MoH.[15] In a recent survey of 655 hospitals across the country by the MoH in 2019, 144 (22%) had developed treatment guidelines based on local evidence, 269 (41%) had adopted the pre-authorization policy (antibiotics requiring approval before use for patient treatment), 189 (29%) developed clinical microbiology guidelines, and 458 (70%) employed basic infection control policy in their facilities.[15]

In a previous qualitative study about AMS implementation in seven hospitals in the national AMR surveillance network, [12 16] we identified the following barriers and opportunities that hospitals in Viet Nam have encountered in setting up and running the programs: a lack of dedicated resources, ineffective communication between

For peer review only - http://bmjopen.bmj.com/site/about/guidelines.xhtml

clinicians, pharmacy and microbiology departments, prescribing habits among doctors, including not taking diagnostic samples before starting antibiotic therapy, not adjusting antibiotics based on culture results, and hesitation and lack of confidence in de-escalating and switching from intravenous to oral therapy. Antibiotics were mainly prescribed based on clinical presentation and progress, doctors' experience, the requirements for health insurance reimbursements and availability of specific drugs at the pharmacy department (unpublished data). Limited data are available on the process of implementation and impact of AMS programs in Viet Nam; only one report from a large hospital has indicated AMS activities could improve patient outcomes.[17]

In this study, we aim to investigate the feasibility of establishing hospital-based AMS programs comprising action-planning, educational interventions and data feedback in two provincial-level hospitals in Viet Nam. We describe the process of preparation, planning and implementation and identify the factors that are important in setting up such a program in hospital settings in the country.

METHODS

Study design and setting

In this implementation research study we employed a participatory action process to establish AMS programs at two provincial-level hospitals in Viet Nam. In this process, hospitals were considered a complex adaptive system consisting of evolving inter-related individuals and non-linear interactions, and the study was conducted under the assumption that interventions cannot be applied in different hospitals with predictable results.[18] The process also included assessments at both national and local levels, and adapted from existing resources from Duke Antimicrobial Stewardship Outreach Network (DASON) in Durham, North Carolina, USA and local guidelines. The implementation period of AMS programs was between 29 July 2020 - 28 July 2021 in one hospital and 01 June 2020 – 31 May 2021 in the other hospital. Baseline assessments were conducted during one year before the implementation period, and evaluation activities were integrated throughout the implementation process.

Implementation approach

For peer review only - http://bmjopen.bmj.com/site/about/guidelines.xhtml

Our planning process began at a national AMS stakeholder meeting organized by the Medical Services Administration of the MoH, World Health Organization and Oxford University Clinical Research Unit (OUCRU), during which we reviewed the current status of AMS implementation in the country. Based on our pre-existing understanding and network with local hospitals, on this meeting, and on subsequent discussions with MoH, we invited two provincial hospitals to join this implementation study: Viet Tiep Hospital in Hai Phong, a large port city with 2 million inhabitants, in the north (2,000-bed capacity, 400,000 out-patient and 100,000 in-patient visits per year) and Dong Thap General Hospital in Cao Lanh, Dong Thap in the Mekong Delta in the south (1000-bed capacity, 710,000 out-patient and 65,000 in-patient visits per year). Hereinafter, these two study hospitals will be intentionally referred to as Hospital 1 and Hospital 2 (in no particular order) to avoid identification of the hospital name in the data presented.

The process of setting up an AMS program at each hospital went through cycles of planning, implementing and evaluating phases (Figure 1). During discussions between the hospitals and the study team, we identified elements of an AMS program that should be common to both hospitals and elements that could be adapted locally. The results of interventions over time were monitored through repeated evaluations of prescription practices, knowledge and attitudes every three months, and interventions could evolve over time to achieve the maximal impact. The AMS team regularly used the collected data and discussed with the study team to identify issues and opportunities for improvement.

Each hospital selected eight clinical wards to participate in the study: four for AMS intervention and four as control wards (the number of wards was determined by the funding availability). The selection of intervention and control wards was based on discussions within the hospital AMS team with two selection criteria: 1) the amount of antibiotic used was greater than average of all wards in the hospital based on pharmacy reported data and 2) the head of ward was willing to participate in the study intervention or control group. The control wards were included to support evaluation of overall AMS outcome indicators (total antibiotic use and for main classes) using trend analysis over 12 months before and after the start of intervention. For practical reasons, there was no strict requirement to identify control wards that were similar to

For peer review only

the intervention wards. After eight wards were identified, the decision on the wards to be in the intervention group and control group was left at the discretion of the director boards and the AMS team in each hospital. At both hospitals, the directors and the AMS teams decided to select the wards that were more likely to accept the AMS interventions based on their judgment and internal relationships. The allocation of the wards to each study group was as follows:

At Hospital 1:

- Intervention group included surgical intensive care unit, traumatology, respiratory/ musculoskelectal system, and infectious diseases ward;
- Control group included internal intensive care unit, surgical nephro-urology, pediatrics and general internal medicine ward;

At Hospital 2:

- Intervention group included surgical intensive care unit, general internal medicine, traumatology, and high-quality-serviced ward (provide treatment services with better quality based on patient's request);
- Control group included internal intensive care unit, oncology, infectious diseases and surgical gastroenterology ward

The preparation and implementation process started with the training and mentoring component, which was then integrated throughout the study life cycle. Training, technical support and mentoring activities were modeled to international AMS models and developed with experts from DASON, and included sharing experiences and identifying opportunities for local changes and interventions to improve antibiotic use and patient outcomes. DASON is community hospital network that supports the implementation of local AMS programs and collaborative network participation has shown reductions in antibiotic use in these hospitals.[19 20]

Training and mentoring

Training and mentoring by local experts on infectious diseases, clinical pharmacists, and clinical microbiologists to support AMS implementation were also integrated into the programs. These experts were from National Hospital for Tropical Diseases, Hanoi (NHTD); Hospital for Tropical Diseases, Ho Chi Minh City (HTD), Viet Nam National Children's Hospital, Hanoi and Hanoi University of Pharmacy, Hanoi.

For peer review only - http://bmjopen.bmj.com/site/about/guidelines.xhtml

The following training events were organized throughout the planning and implementation phases, with topics discussed and agreed upon by all partners involved and timing decided based on practical considerations, including the impact of COVID-19 on routine care in each location:

- A study tour to Duke University was organized for AMS staff of two study hospitals and local AMS experts from NHTD and HTD in July-August 2019. Participants attended lectures presented by DASON experts on AMS principles and practical guidance and experiences on specific AMS interventions. Participants also visited four DASON community hospitals to learn about the AMS models implemented at these sites. Participants also discussed about the feasibility and applicability of the interventions to the Vietnamese context.
- Visits by DASON experts and OUCRU staff at each study hospital in Viet Nam were organized in December 2019 to provide on-site training and technical support in assessing the needs and planning of activities.
- Training courses by local experts on antibiotic treatment, clinical pharmacy and clinical microbiology were organized in a class-room style for doctors, pharmacists and microbiologists. These courses were provided at two time points: one initial course in June 2020 to cover the broad concepts and one follow-up course in October 2020 focusing on the specific needs and knowledge gaps of each hospital.
- One-week training and shadowing for clinical pharmacists on prospective audit and feedback (PAF) at HTD was organized in December 2020.
- Training course on microbiological specimen sampling was organized at Hospital 1 for nurses in April 2021.
- Training and on-going technical support were provided by OUCRU for AMS staff on specific skills in planning and implementation of AMS activities.

Local meetings were organized in each hospital with the participation of AMS team members and participating intervention wards to assess the current gaps and needs and develop plans for implementation. Mid-term meetings and project monitoring meetings were also organized by OUCRU to review on the progress and upcoming activities with the AMS team at each hospital.

For peer review only

Evaluation as part of the implementation cycle

Evaluation was integrated throughout starting with the first round of data collection and analysis before the AMS intervention and every three months after program implementation. The data were used to design and tailor the intervention activities toward the goal in reducing unnecessary antibiotic use in each hospital. Following baseline indicators were evaluated in each hospital (Supplementary Table 1):

- Antibiotic use in total and from targeted agent groups;
- Knowledge and perceptions of doctors;
- Proportions of antibiotic prescriptions which had an indication documented, had review/stop date documented, were used as surgical prophylaxis for greater than 24 hours, were not compliant with guidelines currently endorsed at the hospital, and were inappropriate;
- Proportion of resistant isolates for priority antibiotic-organism combinations;
- Cost of antibiotic treatment and hospitalization;
- In-hospital mortality;
- Hospital length of stay.

Data were collected from the existing routine hospital information systems for 12 months baseline period, knowledge-attitude-practice (KAP) surveys of all doctors in eight study wards and relevant AMS staff (pharmacists and microbiologists), and reviews of medical charts in the four intervention wards by the doctors at each of the wards.

The key process indicators of the project were: 1) a functioning AMS team was formed and was able to lead the AMS activities; 2) a 1-year implementation plan for AMS program was developed and carried out; 3) baseline data and subsequent data from evaluation rounds were used to inform planning and implementation. We recorded implementation data to monitor the intervention activities, specifically PAF implemented by the clinical pharmacists. Other data about trainings and meetings (quantity, number of participants, content) and resources required for the activities (costs, time, staff) were also recorded for monitoring the process of program implementation and support parallel economic evaluation.

For peer review only - http://bmjopen.bmj.com/site/about/guidelines.xhtml

Data analysis and feedback

We reviewed contextual information collected from the national meeting and the baseline needs assessment at the two hospitals and performed an analysis of strengths, weaknesses, opportunities and threats (SWOT) of AMS implementation to support the AMS team in initial planning of the activities at each hospital. We used the following DASON recommendations for the key AMS components to guide our analysis, which include the core elements described by the US CDC [21] and the global recommendations[22]:

Leadership: An individual who is qualified through education, training, or experience in infectious diseases (ID) and/or antibiotic stewardship as leader of AMS program; Committee structures for oversight and executive authority for AMS activities; Formal policy outlining AMS program

Pharmacy: Automated systems (automated dispensing system, bar-code medication administration, computerized physician order entry, electronic medical record, electronic medication administration record); Pharmacy-based dose optimization activities; Regular antibiotic use evaluations; Antibiotic formulary; Pharmacy staffing with clinical pharmacist with specialized training and interest in ID

Microbiology: Laboratory reporting system; Cumulative antibiogram; Microbiology personnel; Testing and incidence of multi-drug resistant organisms

Infection management: ID clinician presence

We performed descriptive data analysis for each hospital on baseline data and data from the subsequent evaluation rounds to identify issues that required attention and opportunities for intervention in the AMS program. Results were summarized and presented to provide feedback to the hospital management board, AMS team, and intervention wards through reports and project progress meetings and to use as evidence for identifying further actions. We characterized antibiotic use by subgroups, types of clinical wards, and international classification of diseases (ICD-10) code groups. Hospital-wide antibiotic susceptibility testing results entered into WHONET were extracted and analyzed following the same analysis protocols performed in previous studies for AMR surveillance data from VINARES hospital network.[9 10] We reported microbiology data following the recommendations of the MICRO checklist [23] as shown in Supplementary Table 2.

For peer review only - http://bmjopen.bmj.com/site/about/guidelines.xhtml

Resistance results were expressed as proportions of resistant isolates out of the number of tested isolates. We classified multidrug resistance (MDR) and carbapenem resistance based on the criteria proposed by Magiorakos et al [24] as follows:

- For *Acinetobacter baumannii* and *Acinetobacter spp.*: MDR is defined as resistant to at least three of the following: cephalosporin (ceftriaxone or cefepime), aminoglycosides (amikacin, gentamicin or tobramycin), ciprofloxacin and carbapenem (imipenem or meropenem); carbapenem resistance is defined as resistant to imipenem or meropenem;
- For *Escherichia coli, Klebsiella pneumoniae,* and *Klebsiella spp.*: MDR is defined as resistant to at least three of the following: carbapenem (ertapenem, imipenem or meropenem), cephalosporin (ceftriaxone or cefepime), aminoglycosides (amikacin, gentamicin or tobramycin) and ciprofloxacin; carbapenem resistance is defined as resistant to ertapenem, imipenem, or meropenem;
- For *Pseudomonas aeruginosa*: MDR is defined as resistant to at least three of the following four agents: imipenem, ceftazidime, ciprofloxacin and tobramycin; carbapenem resistance is resistant to imipenem or meropenem;

Ethical approval

The study protocol was approved by the Oxford University Tropical Research Ethics Committee (OxTREC Reference 526-19), and the Ethics Committee of National Hospital of Tropical Diseases (08/HĐĐĐ-NĐTƯ 31 May 2019). The conduct of this study conformed to the principles embodied in the Declaration of Helsinki.

Patient and Public Involvement

Patient and public involvement was not included in this study as this is a new aspect in the local hospital settings and requires additional research to explore its feasibility and the most appropriate approaches. Initial efforts are being undertaken in parallel to this research for involving patients and the public in AMS in the local hospitals.

Here we present the results of the initial steps in setting up the program and describe the baseline summary indicators from the routine data of the two hospitals. This paper was reported following the StaRI checklist "Standards for Reporting Implementation Studies: the StaRI checklist for completion".[25]

For peer review only - http://bmjopen.bmj.com/site/about/guidelines.xhtml

RESULTS

1. Baseline analysis from the national AMS stakeholder meeting

The meeting was organized in March 2019 to review the current state of AMS implementation nation-wide with participation of a wide range of stakeholders representative for both public and private sectors, and governmental and international partners working or interested in AMS implementation (Supplementary Table 3). The meeting opened a platform for shared discussions on different aspects of implementation and identified the three major areas for actions at the national level: strengthening a supportive policy environment for AMS, developing AMS training curriculum and core AMS related competencies, and establishing coordination and collaboration in AMS within Viet Nam and the region. Further stakeholder meetings were recommended for continued sharing of experience and promote networking and collaboration in AMS locally and regionally, with one such meeting subsequently organized in December 2019 by OUCRU.

2. Baseline analysis at two hospitals and implementation plan

Data on existing processes and structures were reviewed to provide input for initial planning of AMS intervention. In Table 1, we summarized the results of a SWOT analysis conducted during the preparation phase at both hospitals in 2020 of the current state of key components, the needs identified for each component, and the corresponding AMS interventions to be implemented. There was a large gap between the baseline situation in each hospital and the recommendations by DASON in all components. Leadership commitment was identified as a prerequisite for site selection, however the level of participation was different between the two hospitals. At one hospital, a vice director directly leads the AMS program together with the General Planning Department, which drives the implementation activities and encourages the clinical wards to actively be engaged in the program. The process was slower at the other hospital where the program was managed directly by the General Planning Department and International Collaborations Department.

Both hospitals provide care and treatment to all types of clinical conditions and diseases in the catchment areas. Each hospital has a department of infectious diseases, providing care and treatment to patients who are admitted to this department, and

For peer review only - http://bmjopen.bmj.com/site/about/guidelines.xhtml

provide consultation in managing patients with signs of infection in other clinical wards if required. Each hospital also has a department of pharmacy and a microbiology laboratory. In both hospitals, pharmacists were mostly involved in drug inventory and bidding, and some support to doctors in dose adjustment in special cases, and microbiologists' main roles were to inform doctors of test results. Engagement of microbiology, pharmacy and infectious diseases departments in antibiotic treatment was generally passive, mostly through hospital-wide consultation meetings on an ad-hoc basis when there were difficult cases. Local AMR surveillance data was not readily available for use to inform treatment guidelines or updating drug formularies.

From the gaps and needs identified, specific strategies were developed within the first-year plan for interventions targeting each of the key components (Table 1). These strategies included:

1) establishing leadership commitment and forming an AMS coordinating team that can connect all relevant wards and units within each hospital. At both hospitals, the planning department plays a key and coordinating role with a linkage to the specific clinical and functional departments and reporting responsibility to the hospital management board;

2) using data to inform planning and evaluating AMS activities and stimulate the actions among leaders and staff through regular AMS meetings organized at each hospital. Data were from the KAP surveys, review of patient medical charts and routine data on antimicrobial treatment, clinical diseases, antimicrobial susceptibility data and denominator data (days present at each study ward);

3) training/ mentoring activities to update the knowledge and skill gaps for doctors in the intervention wards based on the findings from quarterly KAP surveys and retrospective reviews of patients charts for antibiotic prescribing practices. These trainings were delivered by national experts on infectious diseases, clinical microbiology and clinical pharmacy; technical training for AMS staff on specific skills including setting up an AMS program, using data for planning and monitoring, prospective audit and feedback, communication skills, and developing guidelines provided by DASON experts and local experts;

For peer review only

4) implementing PAF at the intervention wards by clinical pharmacists with support from the AMS doctor and team members. On-site training and shadowing experienced clinical pharmacists in HTD during their PAF activities were organized to develop the capacity for these new teams. By the end of the implementation period, 1890 antibiotic prescriptions were audited in Hospital 1 (time period: June 2020-May 2021; 1.5 fulltime clinical pharmacists) and 1628 in Hospital 2 (July 2020-July 2021; four fulltime clinical pharmacists). Recommendations were made in 82/1890 (4.3%) of the audited cases in Hospital 1 and 128/1628 (7.9%) in Hospital 2. The hospitals planned to continue PAF in the daily work of the clinical pharmacists.

Some strategies differed by site. At Hospital 1, due to limited microbiology data available for developing treatment guidelines the hospital decided to focus firstly on increasing the quantity and quality of specimen cultures through updating the current SOPs and organizing trainings for technicians and nurses at each clinical ward. At Hospital 2 where there are more surgical wards, the hospital AMS team put more focus on surgical prophylaxis with an initial aim to reduce the number of two-drug combination orders.

3. Baseline summary indicators before intervention

Antibiotic use data

Total number of patients admitted at the intervention and control wards at the two hospitals in 2019 and the proportions with antibiotic treatment during the whole hospital admission are summarized in Table 2 following the ICD-10 codes for the main diagnosis recorded for each patient. The spectrum of diseases was by definition heterogeneous among the patients admitted at the intervention and control wards but also in both absolute number and the proportions being prescribed antibiotics. The diagnosis groups with highest amount of antibiotic use in DOT in 2019 for both intervention and control group in two hospitals also differed. The top three for each group were (in brackets, the average number of DOT per 1000 days present):

- Control group in Hospital 1: genitourinary diseases (1057), respiratory diseases (1028), and certain infectious/parasitic diseases (927);

For peer review only - http://bmjopen.bmj.com/site/about/guidelines.xhtml

- Intervention group in Hospital 1: respiratory diseases (897), certain infectious/parasitic diseases (863), and injury, poisoning and certain other consequences of external causes (729);
- Control group in Hospital 2: digestive diseases (800), certain infectious/parasitic diseases (797), and neoplasms (553);
- Intervention group in Hospital 2: respiratory diseases (888), injury, poisoning and certain other consequences of external causes (736), and digestive (707).

Figure 2 displays the monthly distribution of antibiotic use in patients in the intervention and control wards. The amount of antibiotic consumption varied significantly by month in 2019 in the study wards for both hospitals: 951 DOT per 1000 days present in the control compared to 496 in the intervention group in Hospital 1, and 737 in the control compared to 714 in the intervention group in Hospital 2. Hospital 1 used more third-generation cephalosporins than Hospital 2 while Hospital 2 used more penicillin combinations than Hospital 1. In addition, there were more variations across the months in the amount of use for each antibiotic subgroup in Hospital 2 than Hospital 1.

Microbiology data

There were 2430 isolates reported from culture of 10,784 specimens (all types of specimens, 22.5% positive rate) in Hospital 1, with 289 isolates from 3583 blood specimens (8.1% positivity rate) in 2019. Hospital 2 reported 5463 isolates recovered from 23,731 specimens (all types of specimens, 23.0% positive rate) and 1642 isolates recovered from 14,539 blood specimens (11.3% positive rate) in 2019.

Among the study wards, there were 1392 isolates (5.5 isolates per 1000 days present) reported for eight study wards in Hospital 1 (198 blood isolates, 14.2%) in 2019. Slightly more isolates were from male patients (56.6%) and from older age range (median age 63; interquartile range 48-74) in the 8 wards of this hospital. Hospital 2 reported 2433 isolates (13.4 isolates per 1000 days present) for eight study wards in 2019, of which 802 (33.0%) were blood isolates. These isolates were also mainly from male patients (64.3%) and older age range (median age 64; interquartile range 52-76).

For peer review only - http://bmjopen.bmj.com/site/about/guidelines.xhtml
For peer review only

E. coli was the most commonly isolated bacterium in the study wards in both hospitals (Figure 3), and harboured 8.3% (n=540) carbapenem resistance and 34.1% (n=539) MDR (resistant to at least three of the following: carbapenem, cephalosporin, aminoglycosides, and ciprofloxacin) in Hospital 1 and 7.4% (n=297) and 20.6% (n=267) respectively in Hospital 2 (Supplementary Table 4). *K. pneumoniae* in Hospital 2 showed a high level of resistance to carbapenem (in all specimens 52.2% n=289 and in blood 45.2% n=62) and MDR (51.0% n=249 and 44.1% n=59 respectively). For Hospital 1, *Klebsiella spp.* showed lower carbapenem resistance (26.3% n=167) and MDR (39.5% n=167) in all specimens.

High levels of carbapenem resistance in all specimens (80.0% n=325) and in blood (57.9% n=19) and MDR (resistant to at least three of the following: cephalosporin, aminoglycosides, ciprofloxacin and carbapenem) in all specimens (62.6% n=265) and in blood (47.1% n=17) were reported for *A. baumannii* in Hospital 2 and *Acinetobacter spp.* in Hospital 1 (in all specimens 64.1% n=103 carbapenem resistance and 60.8% n=102 MDR).

For *P. aeruginosa* in all specimens, the proportions were 25.3% n=253, 35.0% n=254 and 24.3% n=272 for ceftazidime resistance, carbapenem resistance and MDR (resistant to at least three of the following four agents: imipenem, ceftazidime, ciprofloxacin and tobramycin), respectively in Hospital 2, lower than those in Hospital 1 (41.2% n=85, 41.9% n=86 and 40.7% n=86 respectively).

Proportions of methicillin-resistant *Staphylococcus aureus* were 79.0% (n=210) in Hospital 1 and 76.7% (n=103) in Hospital 2 based on the cefoxitin test.

DISCUSSION

We used an implementation research approach to evaluate the feasibility and understand the process of AMS implementation in two pilot hospitals in Viet Nam. Firstly, this study showed that building a functioning multidisciplinary AMS team is an important factor for the success of the program. In Viet Nam, AMS committees have been formed in 334 out of 655 hospitals (51%) in a recent MoH report in 2019, however the majority of these committees had irregular activity (72%).[15] In our study, AMS teams were led by hospital director/vice-director and the planning department,

For peer review only

which has the advising role to the director-board. These combinations were routed from the traditional set-up of Vietnamese hospitals and appeared to work effectively in most hospital-wide programs. SWOT analysis, a recommended tool in the WHO Resources for AMS programs in health-care facilities in LMICs[6], was used in our needs assessment and has proved to effectively help our hospital AMS teams identify the needs and focus priority interventions.

Secondly, the partnership approach combining local and international partners in our study demonstrated an effective synergy of experience, expertise and resources to ensure the appropriate selection of strategies and methods in setting up AMS programs in LMICs. This approach has been advocated in a recent call for international collaborations in AMS implementation in LMICs, which can be achieved through the dissemination of high-quality research and educational resources recognizing the needs to contextualize to the local conditions.[26] Our study provided further evidence to support this call for greater international collaboration and partnerships to facilitate shared expertise and knowledge for AMS.

Across a number of AMS programs worldwide, pharmacists are often included to manage the prescription-based strategies including pre-authorization formularies and post-prescription audits and feedback (PAF). This was reflected in the national guideline on AMS implementation originally issued in 2016 and updated in 2020.[13 14] Sustained reductions have been achieved in antibiotic use by AMS teams across different settings including the example of non-specialist pharmacist-led program in South Africa[27] and clinician-led and clinical pharmacist-driven AMS program in India.[28] In our study, despite having no experience in clinical interactions, our junior pharmacists have been building their PAF capacity and gained promising results in the number of audits with feedback to the doctors. Although the results were highly variable between hospitals, this shows the feasibility and potential of involving pharmacists in the prescription-based strategies for AMS programs in LMICs. A recent prospective multicenter study in community hospitals in the USA has also shown the effective role of pharmacists in implementing the pre-authorization and post-prescription audit and feedback in improving antibiotic use in resource-limited settings.[29]

For peer review only - http://bmjopen.bmj.com/site/about/guidelines.xhtml

AMS programs in LMICs generally encounter challenges of limited financial and physical resources available for implementation including substandard lab practices, lack of well-trained staff, insufficient opportunities for continuing professional development, and difficulties in deploying prescription-based stewardship strategies.[26 27] Despite numerous challenges and limited, poor-quality evidence available [30], effective AMS programs can be feasible in these settings although contextualization is essential in this implementation.[7] The need for contextualization in LMICs was also highlighted in several qualitative and mixed-methods AMS studies.[31-33] In our program, AMS implementation relied on external financial support with the funded intervention period planned for one year. Considerations were made regarding choosing the most effective strategies that can support ongoing AMS team operations and continued AMS activities after the funding period ends.

Among these considerations, capacity building and training for the AMS team in program planning and evaluation and hospital pharmacists to conduct PAF activities were identified as the key components. As recommended in a recent review of AMS program designs, practically integrating the planned changes into the normal routine clinical practices is the best way to achieve sustainability apart from securing long-term resources.[34] Making PAF activities routine can contribute to the sustainability of the AMS program. In addition, developing methods and staff capacity to utilize the existing routinely collected data (clinical, pharmacy, and microbiology) was also a priority in our program. Nevertheless, at both study hospitals, the outstanding issue remained in the use of local evidence to inform practices in antibiotic treatment and management, and a lack of information technology and epidemiological capacity partly contributes to this issue. This issue will need to be addressed in the next longer-term AMS program plan at both hospitals.

AMS programs have been traditionally "pharmacy-centric" with minimal or no input from microbiology; participation of clinical microbiologists in AMS teams varied widely around the globe (20-90% across the surveys in Europe, Australia, US and Canada).[35] Microbiology capacity and surveillance and control of multi-drug resistant bacteria is a common challenge in AMS implementation for hospitals in LIMCs [34 36], and this is particularly the case for provincial and community level hospitals in Viet Nam. From the MoH survey in 2019, 90% of hospitals had a pharmacist while only

For peer review only - http://bmjopen.bmj.com/site/about/guidelines.xhtml

39% included a microbiologist in their AMS team. The current status of microbiology in LMICs would not allow for a desirable level of support to AMS programs.[26] However, improvements can be made even within resource-constrained conditions. Our study showed that simply compiling the current data and presenting the summary data in the AMS meetings has stimulated the discussions on the immediate and long-term actions to improve the problem. The participants realized the need to have good microbiology data as a foundation for more effective and targeted interventions, and the first simple step was to review the procedures, organize trainings, and encourage doctors to perform specimen cultures prior to initiating antibiotic treatment. Such changes have been made feasible by an active AMS team with motivated team leaders which is considered as a prerequisite for the success of AMR containment interventions in LMIC settings.[37]

The strengths of our study include the participatory approach in AMS implementation where hospitals were considered as a complex adaptive system with unique characteristics and interconnections between players that could be leveraged to develop locally tailored interventions for maximal results.[18] These included identifying strategies that could be sustained relying on local resources as routine practices including PAF and data use. Secondly, the study has employed inter-disciplinary and team activities in both implementation and evaluation of AMS program, and emphasized the need to improve the capacity of all stakeholders involved in each hospital setting. Thirdly, our implementation approach was designed to include perspectives and expertise from both policy makers to hospitals at different levels and from international and local partners. Our data from the implementation process were consistent with and consolidating the outcomes from the discussions in the national stakeholder meeting.

Our study, however, shares the common limitation of implementation research, which is the ability to generalize the obtained knowledge of AMS implementation to different healthcare settings. Our evidence in two provincial hospitals in Viet Nam will be generalizable to the community hospitals with medium levels of resources and staff available for implementation but lack of staff training and capacity in carrying out AMS activities. Nevertheless, the nature of implementation research in recognizing and tailoring activities to the local contexts is an important aspect in

For peer review only - http://bmjopen.bmj.com/site/about/guidelines.xhtml

bridging the gap in our understanding of what is known to be effective and what works in the actual patient care settings.[18] Further implementation research in other hospital and healthcare settings is required to provide more data to fill the know-how gap in AMS programs in LMICs.

In conclusion, AMS implementation requires an in-depth understanding of the local conditions and practices and the contextualization of the intervention strategies in Viet Nam and similar LMIC settings. We showed that using implementation research design is an effective approach in assessing the feasibility and evaluation of the AMS program. It also helps local hospitals understand their needs and start prioritizing AMS in the local agenda, paving the solid steps for a long-term institutionalization and improvement of the program. As evident in our process, it is important to develop the skills and capacity of the multidisciplinary AMS team and identify creative combinations of strategies that could be carried out with locally available resources for maximal and sustainable outcomes.

Acknowledgements

We acknowledge the invaluable support and collaboration of the Medical Services Administration of the Viet Nam Ministry of Health and the World Health Organization Office in Viet Nam during the planning of this study.

Funding

Pfizer Independent Grants for Learning & Change (IGLC) provides all project funding and The Joint Commission provides administrative oversight for the program; H.V.T.L was also supported by the National Institute for Health Research (NIHR) (using the UK's Official Development Assistance (ODA) Funding) and Wellcome (Grant Reference Number: 216367/Z/19/Z) under the NIHR-Wellcome Partnership for Global Health Research. The views expressed are those of the authors and not necessarily those of Wellcome, the NIHR or the Department of Health and Social Care.

Competing interests

None declared.

For peer review only - http://bmjopen.bmj.com/site/about/guidelines.xhtml

Contributors

VTLH, HRvD, and DJA obtained funding and contributed to all aspects of the study design, with input from LMQ, VHV, and CMD. VTLH and HRvD had overall responsibility for the study. TTTH, VHV, VTHDE, TTDN and HPT coordinated the running of the study with support from NTH, TD, PVBB, NTTO, PTTH, NTCT, and TAQ. VTLH analysed the data with input from HRvD, DJA, EDA, and TK. VTLH was responsible for the drafting of the manuscript. All authors gave approval for the final version of the manuscript.

Patient consent for publication

Not required for this research.

ORCID IDs

Vu Thi Lan Huong 0000-0002-9579-5576

H. Rogier van Doorn 0000-0002-9807-1821

Vu Hai Vinh 0000-0001-6130-7864

Data availability

Summary data from this study could be made available upon reasonable request.

REFERENCES

1. Schuts EC, Hulscher MEJL, Mouton JW, et al. Current evidence on hospital antimicrobial stewardship objectives: a systematic review and meta-analysis. The Lancet Infectious Diseases 2016;**16**(7):847-56.

2. Davey P, Marwick CA, Scott CL, et al. Interventions to improve antibiotic prescribing practcies for hospitals inpatents. Cochrane Database Syst Rev 2017;**2: CD003543**.

3. Wagner B, Filice GA, Drekonja D, et al. Antimicrobial Stewardship Programs in Inpatient Hospital Settings: A Systematic Review. Infection Control & Hospital Epidemiology 2016;**35**(10):1209-28.

4. Dik J-WH, Vemer P, Friedrich AW, et al. Financial evaluations of antibiotic stewardship programs—a systematic review. Frontiers in Microbiology 2015;**6**(317).

For peer review only - http://bmjopen.bmj.com/site/about/guidelines.xhtml

5. Kaki R, Elligsen M, Walker S, et al. Impact of antimicrobial stewardship in critical care: a systematic review. Journal of Antimicrobial Chemotherapy 2011;**66**(6):1223-30.
6. Pierce J, Apisarnthanarak A, Schellack N, et al. Global Antimicrobial Stewardship with a Focus on Low- and Middle-Income Countries. Int J Infect Dis 2020;**96**:621-29.
7. Cox JA, Vlieghe E, Mendelson M, et al. Antibiotic stewardship in low- and middle-income countries: the same but different? Clin Microbiol Infect 2017;**23**(11):812-18.
8. Kim SH, Song JH, Chung DR, et al. Changing trends in antimicrobial resistance and serotypes of Streptococcus pneumoniae isolates in Asian countries: an Asian Network for Surveillance of Resistant Pathogens (ANSORP) study. Antimicrob Agents Chemother 2012;**56**(3):1418-26.
9. Vu TVD, Do TTN, Rydell U, et al. Antimicrobial susceptibility testing and antibiotic consumption results from 16 hospitals in Viet Nam: The VINARES project 2012–2013. J Glob Antimicrob Resist 2019;**18**:269-78.
10. Dung VTV, et al. Antimicrobial susceptibility testing results from 13 hospitals in Viet Nam: VINARES 2016-2017. Antimicrob Resist Infect Control 2021.
11. Phu VD, Wertheim HFL, Larsson M, et al. Burden of Hospital Acquired Infections and Antimicrobial Use in Vietnamese Adult Intensive Care Units. PLoS One 2016;**11**(1):e0147544.
12. Kinh NV, Wertheim HFL, Thwaites GE, et al. Developing an antimicrobial resistance reference laboratory and surveillance programme in Vietnam. Lancet Glob Health 2017;**5**(12):e1186-e87.
13. Viet Nam Ministry of Health. Quyết định về việc ban hành tài liệu "Hướng dẫn thực hiện quản lý sử dụng kháng sinh trong bệnh viện" [Decision on the issuance of the document "Guideline on implementing antimicrobial stewardship in hospitals"]. Hanoi: Viet Nam Ministry of Health, 2016.
14. Viet Nam Ministry of Health. Quyết định về việc ban hành tài liệu "Hướng dẫn thực hiện quản lý sử dụng kháng sinh trong bệnh viện" [Decision on the issuance of the document "Guideline on implementing antimicrobial stewardship in hospitals"] Numbered 5631/QD-BYT. In: Health Mo, ed. Ha Noi: Ministry of Health, 2020.
15. Hanoi Hospital Pharmacy Association, ed. Thực trạng triển khai chương trình quản lý sử dụng kháng sinh tại các bệnh viện trên toàn quốc [Status of implementing antimicrobial stewardship programs in hospitals across Viet Nam]. Open Hanoi

For peer review only

Hospital Pharmacy Scientific Conference; 2019; Hanoi. Hanoi Hospital Pharmacy Association.

16. Wertheim, H., et al., Providing impetus, tools and guidance to strengthen national capacity for antimicrobial stewardship in Viet Nam. PLoS Med 2013; **10**(5): e1001429.

17. Son NT, Tra TT, Thao PTN. Antimicrobial Stewardship Program at a tertiary teaching hospital in Vietnam: A longitudinal observational study. Clin Microbiol Infect Dis 2017;**2**: doi: 10.15761/CMID.1000121.

18. Leykum LK, Pugh Ja Fau - Lanham HJ, Lanham Hj Fau - Harmon J, et al. Implementation research design: integrating participatory action research into randomized controlled trials. Implement Sci 2009;**4**(69).

19. Sexton DJ, Moehring RW. Implementation of Antimicrobial Stewardship Programs in Small Community Hospitals: Recognizing the Barriers and Meeting the Challenge. Clin Infect Dis;**65**(4):697-98.

20. Yarrington M, Dodds Ashley E, Johnson M, et al. 2089. Effect of the Duke Antimicrobial Stewardship Outreach Network (DASON): A Multi-Center Time Series Analysis. Open Forum Infectious Diseases 2019;**6**(Suppl 2):S705-S06.

21. (CDC) CfDCaP. Core Elements of Hospital Antibiotic Stewardship Programs, https://www.cdc.gov/antibiotic-use/core-elements/hospital.html. 2019.

22. Pulcini C, Binda F, Lamkang AS, et al. Developing core elements and checklist items for global hospital antimicrobial stewardship programmes: a consensus approach. Clin Microbiol Infect 2018.

23. Turner P, Fox-Lewis A, Shrestha P, et al. Microbiology Investigation Criteria for Reporting Objectively (MICRO): a framework for the reporting and interpretation of clinical microbiology data. BMC Med 2019;**17**(1):70.

24. Magiorakos AP, Srinivasan A, Carey RB, et al. Multidrug-resistant, extensively drug-resistant and pandrug-resistant bacteria: an international expert proposal for interim standard definitions for acquired resistance. Clin Microbiol Infect 2012;**18**(3):268-81.

25. Pinnock H, Barwick M, Carpenter CR, et al. Standards for Reporting Implementation Studies (StaRI) Statement. BMJ 2017;**356**(i6795).

26. Tattevin P, Levy Hara G, Toumi A, et al. Advocacy for Increased International Efforts for Antimicrobial Stewardship Actions in Low-and Middle-Income Countries on Behalf of Alliance for the Prudent Use of Antimicrobials (APUA), Under the

For peer review only - http://bmjopen.bmj.com/site/about/guidelines.xhtml

Auspices of the International Society of Antimicrobial Chemotherapy (ISAC). Front Med (Lausanne) 2020;**7**:503-03.

27. Laxminarayan R, Van Boeckel T, Frost I, et al. The Lancet Infectious Diseases commission on antimicrobial resistance: 6 years later. Lancet Infect Dis 2020;**20**(4):e51-e60.

28. Nampoothiri V, Sudhir AS, Joseph MV, et al. Mapping the Implementation of a Clinical Pharmacist-Driven Antimicrobial Stewardship Programme at a Tertiary Care Centre in South India. Antibiotics 2021; 10(2): 220.

29. Anderson DJ, Watson S, Moehring RW, et al. Feasibility of Core Antimicrobial Stewardship Interventions in Community Hospitals. JAMA Netw Open 2019;**2(8)**(2574-3805 (Electronic)):e199369.

30. Van Dijck C, Vlieghe E, Cox JA. Antibiotic stewardship interventions in hospitals in low-and middle-income countries: a systematic review. Bull World Health Organ 2018;**96**(4):266-80.

31. Charani E, Smith I, Skodvin B, et al. Investigating the cultural and contextual determinants of antimicrobial stewardship programmes across low-, middle- and high-income countries-A qualitative study. PLoS One 2019;**14**(1):e0209847.

32. Gebretekle G, Haile Mariam D, Abebe W, et al. Opportunities and barriers to implementing antibiotic stewardship in low and middle-income countries: Lessons from a mixed-methods study in a tertiary care hospital in Ethiopia. PLoS One 2018;**13**(12):e0208447.

33. Mathew P, Ranjalkar J, Chandy SJ. Challenges in Implementing Antimicrobial Stewardship Programmes at Secondary Level Hospitals in India: An Exploratory Study. Front Public Health 2020;**8**(542):493904.

34. Resman F. Antimicrobial stewardship programs; a two-part narrative review of step-wise design and issues of controversy Part I: step-wise design of an antimicrobial stewardship program. Ther Adv Infect Dis 2020;**7**:2049936120945083.

35. Morency-Potvin P, Schwartz DN, Weinstein RA. Antimicrobial Stewardship: How the Microbiology Laboratory Can Right the Ship. Clin Microbiol Rev 2017;**30**(1):381.

36. Nkengasong JN, Yao K, Onyebujoh P. Laboratory medicine in low-income and middle-income countries: progress and challenges. Lancet 2018;**391**(10133):1873-75.

For peer review only - http://bmjopen.bmj.com/site/about/guidelines.xhtml

37. Singh N, Manchanda V. Control of multidrug-resistant Gram-negative bacteria in low- and middle-income countries-high impact interventions without much resources. Clin Microbiol Infect 2017;**23**(4):216-18.

Tables

For peer review only

Table 1. SWOT analysis of current status of key components, assessment of needs and interventions implemented at two provincial hospitals

**Component**	**SWOT analysis (Strength – Weakness – Opportunity – Threat)**	**Assessment of needs**	**Interventions implemented**
**Hospital 1**			
Leadership	S: Leadership commitment; Vice-director had an active role in AMS committee;<br>W: No AMS experience, large committee with no dedicated AMS coordinator for day-to-day activities<br>O: Guidelines from MoH and experience from other higher-level hospitals; leadership intention to provide AMS outreach support to district hospitals in long term<br>T: Disease outbreaks (e.g. COVID-19) shifting management priorities	Knowledge and skills in planning and implementing AMS;<br>Identify clear roles in planning and implementing AMS activities;<br>Identify targets for interventions	Establishing AMS action team for day-to-day activities with active coordination from Planning Department;<br>Regular minuted team meetings;<br>Training for AMS committee by international and local experts;<br>Collecting and reviewing data to inform interventions
Pharmacy	S: Drug inventories and bidding;<br>W: No clinical experience; only tracking drug purchasing but not actual drug use data; lack of analytical skills<br>O: Electronic data on patient-level drug administration available from clinical database; Government policy and guidelines (clinical pharmacy, AMS guidelines)<br>T: High staff turnover, lack of clinical pharmacists, bidding process and insurance limits (can affect drug choices and prescribing behaviors)	Identify staff for clinical pharmacist roles;<br>Build clinical pharmacy capacity and data analysis skills	Training for clinical pharmacists in antibiotic treatment, microbiology, pharmacology, and review of antibiotic prescriptions (class-room, hands-on);<br>Implemented prospective audit and feedback as a routine activity of clinical pharmacy program;<br>Participation of clinical pharmacists in clinical ward monthly meeting for which they had to analyze audit data and present to the doctors;<br>Support from DASON on using web-based data visualization and benchmarking tool*;
Microbiology	S: In-house microbiology lab; technical support by OUCRU; use WHONET database<br>W: Contamination issues, low test utilization; lack of clinical interactions; no clinical microbiologist; performing manual culture; limited quality control<br>O: Close connection with OUCRU lab; a new lab under construction<br>T: Lack of compliance in sample collection from clinical wards; Weak voice in planning and ordering reagents and tests; insurance limits (can affect culture ordering behavior)	Improve quantity and quality of routine clinical specimens, and quality of microbiology testing;<br>Active planning from microbiology lab;<br>Active communication from lab to clinical wards on patient's specimens and test results;<br>More regular tailored updates on local AMR data to support doctors in empiric treatment;	Training on clinical microbiology for lab staff by locally recognized experts;<br>Review/update SOPs with support from OUCRU lab;<br>Nurse training on collecting clinical specimens;<br>Support from OUCRU lab to review the current microbiology practices and planning for improvement;<br>Analysis of WHONET data twice yearly and creating readable summary of AMR patterns to doctors;
Infection management	S: Hospital-wide consultations with ID doctor available; Head of ID is in the AMS committee<br>W: Limited access to training<br>O: Established connection with HCMC Hospital for Tropical Diseases	Update knowledge and skills in management of specific and locally relevant infectious diseases, clinical pharmacology, and antibiotic treatment guidelines;	Training on infectious diseases management and antibiotic treatment for main clinical syndromes, principals of clinical pharmacology and microbiology, and surgical prophylaxis.

For peer review only

Component	SWOT analysis (Strength – Weakness – Opportunity – Threat)	Assessment of needs	Interventions implemented
	T: Disease outbreaks (e.g. COVID-19)		
**Hospital 2**			
Leadership	S: Leadership commitment;<br>W: No AMS experience, large committee with no dedicated AMS coordinator for day-to-day activities;<br>O: MoH guidelines and experience from other higher-level hospitals; Leadership dedication to high-quality hospital performance<br>T: Disease outbreaks (e.g. COVID-19) shifting management priorities; Hospital's focus on surgical services and cancer treatment	Knowledge and skills in planning and implementing AMS;<br>Identify clear roles in planning and implementing AMS activities;<br>Raise the awareness and stimulate interests of surgical doctors in AMS<br>Identify targets for interventions;	Establishing AMS action team for day-to-day activities with active coordination from Planning Department and International Relations Department;<br>Select four surgical wards to participate in the study (2 intervention and 2 control wards)<br>Training for AMS team by international & local experts;<br>Collecting and reviewing data to inform interventions
Pharmacy	S: Drug inventories and bidding; Young pharmacists available; Hospital has interest in improving clinical pharmacy program;<br>W: Limited clinical experience; only tracking drug purchasing but not actual drug use data; lack of analytical skills<br>O: Electronic data on patient-level drug administration available from clinical database; Government policy and guidelines (clinical pharmacy, AMS guidelines); Close connection with the Hanoi University of Pharmacy for clinical pharmacy training;<br>T: Non-compliance from surgical departments; bidding process and insurance limits (can affect drug choices and prescribing behaviors)	Identify staff for clinical pharmacist roles;<br>Build clinical pharmacy capacity and data analysis skills	Training for clinical pharmacists in antibiotic treatment, microbiology, pharmacology, and review of antibiotic prescriptions (class-room, hands-on);<br>Implemented prospective audit and feedback as a routine activity of clinical pharmacy program;<br>Participation of clinical pharmacists in clinical ward monthly meeting for which they had to analyze audit data and present to the doctors;<br>Join doctors in their clinical ward rounds to get more clinical experience and interactions with doctors;<br>Support from DASON on using web-based data visualization and benchmarking tool*;
Microbiology	S: In-house good-quality microbiology lab; technical support by OUCRU and US CDC; use WHONET database<br>W: Limited clinical interactions;<br>O: Close connection with OUCRU lab; have access to advanced techniques and skills;<br>T: Lack of compliance in sample collection from some clinical wards;	Increase communication with doctors on patient's specimens and test results;<br>Increase clinical experience for clinical microbiologists;<br>More regular tailored updates on AMR data to support doctors in empiric treatment;	Experience sharing and discussions for lab staff with locally recognized experts;<br>Analysis of WHONET data twice yearly and creating readable summary of AMR patterns to doctors;<br>Join doctors in their clinical ward rounds to get more experience and interactions with doctors;
Infection management	S: Hospital-wide consultations with ID doctor and specific specialists available; Strong experience from ICU doctors in difficult cases;<br>W: No presence of ID doctor in AMS committee;<br>O: Established connection with national-level hospitals<br>T: Disease outbreaks (e.g. COVID-19); Dominance of surgical and ICU doctors in determining antibiotic choices for patients	Update knowledge and skills in management of specific and locally relevant infectious diseases, clinical pharmacology, and antibiotic treatment guidelines;<br>Increase the interests and awareness of surgical doctors in AMS	Training on infectious diseases management and antibiotic treatment for main clinical syndromes, principals of clinical pharmacology and microbiology, and surgical prophylaxis for young doctors.<br>Set the target for reducing use of two or more antibiotics for surgical prophylaxis in traumatology department;

For peer review only - http://bmjopen.bmj.com/site/about/guidelines.xhtml

*delayed due to discussions on data sending outside of Viet Nam and where the tool could be located.

For peer review only

Table 2. Number of patients and proportions of antibiotic use by diagnosis groups at intervention and control wards at 2 hospitals between January and December in 2019 before the intervention period

Diagnosis	Hospital 1				Hospital 2			
	Intervention wards*		Control wards**		Intervention wards#		Control wards##	
	n	%	n	%	n	%	n	%
**ICD Diagnosis Group**								
Certain conditions originating in the perinatal period	35	45.7%	1292	81.4%	10	30.0%	3	66.7%
Certain infectious and parasitic diseases	4756	47.3%	3712	79.3%	308	77.3%	2131	59.6%
Congenital malformations, deformations and chromosomal abnormalities					19	47.4%	8	75.0%
Diseases of the blood and blood-forming organs and certain disorders involving the immune mechanism	20	60.0%	405	23.2%	41	22.0%	19	47.4%
Diseases of the circulatory system	146	79.5%	1828	36.8%	818	35.5%	223	84.8%
Diseases of the digestive system	2568	87.3%	2673	25.0%	676	71.3%	2451	83.6%
Diseases of the ear and mastoid process	38	81.6%	26	26.9%	510	12.4%	3	33.3%
Diseases of the eye and adnexa	2	100.0%	5	100.0%	7	42.9%	1	0.0%
Diseases of the genitourinary system	852	63.0%	1790	71.5%	253	93.3%	82	86.6%
Diseases of the musculoskeletal system and connective tissue	473	35.3%	213	26.8%	83	25.3%	17	58.8%
Diseases of the nervous system	136	72.8%	115	49.6%	136	25.0%	95	86.3%
Diseases of the respiratory system	2039	82.9%	7366	95.2%	813	93.0%	856	93.3%
Diseases of the skin and subcutaneous tissue	195	87.7%	433	68.4%	35	65.7%	20	90.0%
Endocrine, nutritional and metabolic diseases	203	74.9%	322	25.5%	140	30.0%	39	69.2%
External causes of morbidity and mortality	13	69.2%	302	35.4%	5	20.0%	41	70.7%
Factors influencing health status and contact with health services	17	100.0%	3	66.7%	6	16.7%	3	66.7%
Injury, poisoning and certain other consequences of external causes	3504	79.9%	202	40.1%	327	83.5%	120	70.8%
Mental, Behavioral and Neurodevelopmental disorders	3	100.0%	17	52.9%	35	8.6%	8	37.5%
Neoplasms	912	79.3%	409	62.6%	252	61.1%	9186	21.8%
Pregnancy, childbirth and the puerperium	3905	79.3%	4	100.0%	7	71.4%	3	66.7%
Symptoms, signs and abnormal clinical and laboratory findings, not elsewhere classified	224	79.3%	716	76.8%	178	78.1%	673	92.3%
Patients diagnosed with viral causes								
Dengue	1503	9.2%	187	28.9%	28	21.4%	408	21.6%
Influenza	1310	92.1%	4497	87.1%	574	93.0%	673	97.3%
Other viral diagnoses	3213	27.1%	1769	63.4%	169	68.6%	1316	40.7%

n: total number of patients, %: proportion of patients with antibiotic treatment; *include surgical intensive care unit, traumatology, respiratory/ musculoskelectal system, and infectious diseases ward; **include internal intensive care unit, surgical nephro-urology, pediatrics and general internal medicine; #include surgical intensive care unit, general internal medicine, traumatology, and high-quality-serviced ward (provide treatment services with better quality based on patient's request); ##include internal intensive care unit, oncology, infectious diseases and surgical gastroenterology ward

For peer review only

For peer review only - http://bmjopen.bmj.com/site/about/guidelines.xhtml

Figures

Figure 1. Approach to AMS implementation in provincial hospitals and roles of partners

Figure 2. Baseline monthly distribution of antibiotic use in Days of Therapy (DOT) in 2019 calculated for all patients with antibiotic treatment administered during their admission at the study wards in each hospital per 1000 days present at these wards stratified by study group and antibiotic subgroups

Figure 3. Carbapenem resistance (CR) and multi-drug resistance (MDR) proportions among key bacteria isolated in study wards at two hospitals in 2019. Bacteria include: *Acinetobacter baumannii* (aba) or *Acinetobacter spp.* (ac-), *Escherichia coli* (eco), *Klebsiella pneumoniae* (kpn), or *Klebsiella spp.* (kl-), and *Pseudomonas aeruginosa* (pae).

For peer review only

For peer review only - http://bmjopen.bmj.com/site/about/guidelines.xhtml

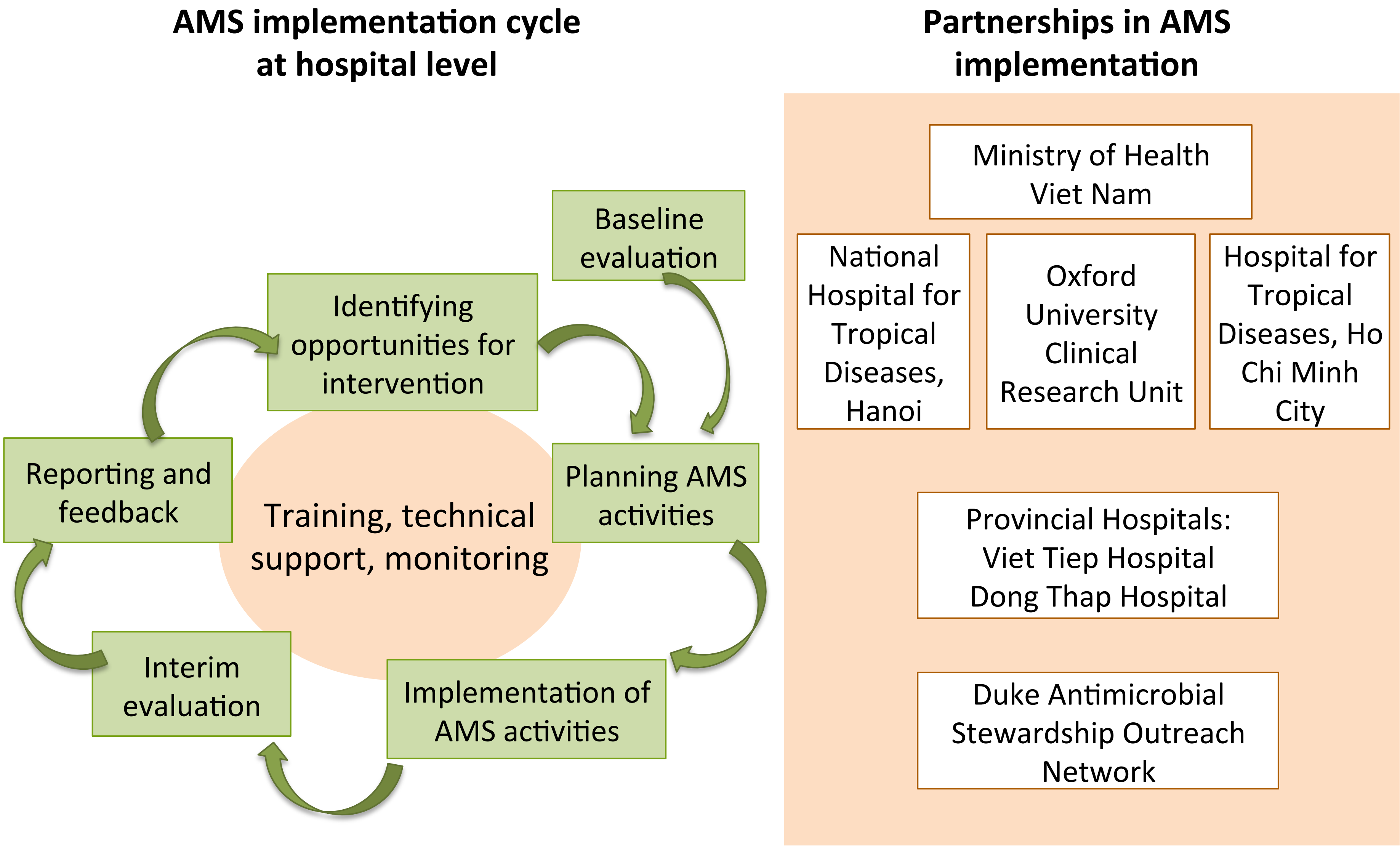
AMS implementation cycle at hospital level
Baseline evaluation
Identifying opportunities for intervention
Planning AMS activities
Implementation of AMS activities
Interim evaluation
Reporting and feedback
Training, technical support, monitoring
Partnerships in AMS implementation
Ministry of Health Viet Nam
National Hospital for Tropical Diseases, Hanoi
Oxford University Clinical Research Unit
Hospital for Tropical Diseases, Ho Chi Minh City
Provincial Hospitals: Viet Tiep Hospital Dong Thap Hospital
Duke Antimicrobial Stewardship Outreach Network

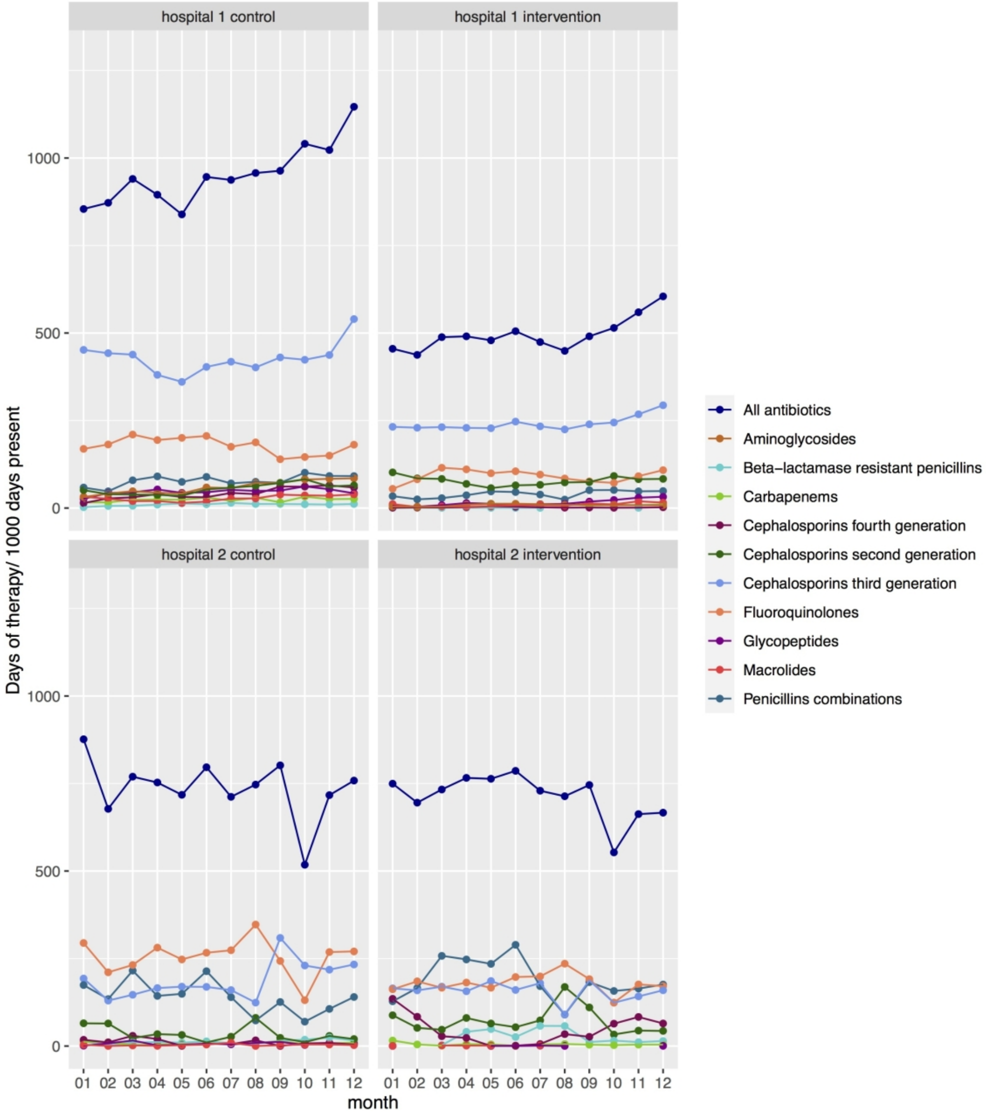

Figure 2. Monthly distribution of antibiotic use in Days of Therapy (DOT) calculated for all patients with antibiotic treatment administered during their admission at the study wards in each hospital per 1000 days present at these wards stratified by study group and antibiotic subgroups

210x238mm (300 x 300 DPI)

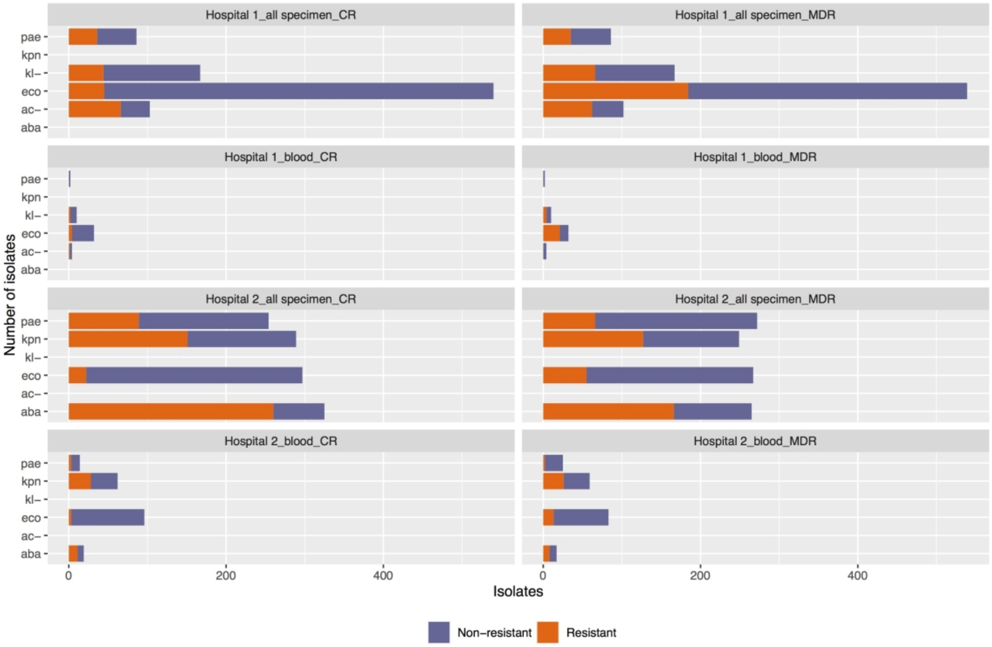

Figure 3. Carbapenem resistance (CR) and multi-drug resistance (MDR) proportions among key bacteria isolated in study wards at two hospitals in 2019. Bacteria include: Acinetobacter baumannii (aba) or Acinetobacter spp. (ac-), Escherichia coli (eco), Klebsiella pneumoniae (kpn), or Klebsiella spp. (kl-), and Pseudomonas aeruginosa (pae).

265x175mm (300 x 300 DPI)

Supplemental Material

Manuscript title: Establishing antimicrobial stewardship programs in 2 provincial-level hospitals in Viet Nam through an implementation research approach

For peer review only

Supplementary Table 1. Study outcome measures and types of data collection

Outcome	Population/ sub-group	Type of data collection
Primary outcome		
Amount of targeted agent groups and total antibiotic use	Intervention and control wards	Extraction of routine data from hospital information systems for 12 months before and 12 months after the start of intervention period
Secondary outcomes		
Changes in knowledge and perceptions of doctors before and after intervention	Intervention wards and control wards	Pre and post-intervention knowledge-attitude-practice (KAP) survey among doctors and pharmacists every 3 months
Proportions of antibiotic prescriptions which:<br>- had an indication documented<br>- had review/stop date documented<br>- were used as surgical prophylaxis for greater than 24 hours<br>- were not compliant with guidelines currently endorsed at the hospital<br>- were inappropriate	Intervention wards	Retrospective medical chart review of 200 patients receiving antibiotics in 4 intervention wards (pre and post-intervention every 3 months)
Proportion of resistant isolates for priority antibiotic-organism combinations	Intervention and control wards	WHONET data – 12 months before and 12 months after intervention
Cost of antibiotic treatment and hospitalizations<br>In-hospital mortality<br>Hospital length of stay	Intervention and control departments	Extraction of routine data from hospital information systems for 12 months before and 12 months after the start of intervention period

*categorized as suboptimal (there may be a mild or non-life-threatening allergy mismatch; or antibiotic prescription is an unreasonable choice for the likely causative or cultured pathogens) or inadequate (antibiotic prescription is unlikely to treat the likely causative or cultured pathogens; or documented or presumed indication does not require any antibiotic treatment; there may be a severe or possibly life-threatening allergy mismatch, or the potential risk of toxicity due to drug interaction; for surgical prophylaxis, the duration is greater than 24 hours (except where local guidelines endorse this) (Source: National Antimicrobial Prescribing Survey (NAPS) - Australia; https://www.naps.org.au/Default.asp)

For peer review only

Supplementary Table 2. Assessment of criteria for clinical microbiology data reported in the study using the Microbiology Investigation Criteria for Reporting Objectively (MICRO) framework

Item	Number	Recommendation	Assessment of microbiology data reported in this manuscript
Methods			
Study design	1*	Specimen types: Describe the types of specimen included, i.e. clinical (e.g. blood cultures) or non-diagnostic surveillance (e.g. admission and other screening swabs to diagnose carriage). If specimens were obtained for diagnostic reasons, clinical syndromes should be described where possible, and specimens/isolates stratified by clinical syndrome.	We used data from microbiology laboratory on the antimicrobial susceptibility testing results (from WHONET software) of routine clinical specimens collected for diagnostic reasons from any patients admitted to the hospital. Data were not recorded on the syndromes of patients tested. Screening samples for carriage is not common practice in these hospitals, so one should assume that all samples have been taken for diagnostic purposes.
	2*	Sampling period: State the collection timeframe for specimens yielding isolates for which data is reported, e.g. from MM/YY to MM/YY to be able to identify variability between seasons.	Data reported in this manuscript were for patients admitted to the hospitals in the year 2019 (01/JAN/2019 to 31/DEC/2019).
	3*	Sampling strategy: Describe the strategy for specimen collection, e.g. asymptomatic screening, sampling of all febrile patients, sampling at clinician discretion, sampling of specific patient groups and convenience sampling (e.g. use of isolates from an existing sample repository). Specify whether sampling followed routine clinical practice or was protocol driven. Classify specimens as from community-acquired (CAI) or hospital-acquired (HAI) infections. The definition of HAI used (e.g. HAI defined by specimen collection > 48 h after hospital admission) should be provided and should use ideally an international standard (e.g. US Centers for Disease Control).	Data were from routine sampling of specimens from any patients admitted at the hospital at clinician discretion. The data didn't allow for classification in HAI vs CAI.
	4	Target organisms: Explicitly state which organisms/organism groups were included in the report. Nomenclature should follow international standards (i.e. using approved genus/species names as summarised in the International Journal of Systematic and Evolutionary Microbiology). Lists of approved bacterial names can be downloaded from Prokaryotic Nomenclature Up-to-Date (https://www.dsmz.de/bacterial-diversity/prokaryotic-nomenclature-up-	Data was collected for all organisms identified at the routine microbiology lab of each hospital. The 5 species most frequently isolated were kept in the analysis and the present report: *Escherichia coli*, *Staphylococcus aureus*, *Klebsiella pneumoniae*, *Acinetobacter baumanii*, and *Pseudomonas aeruginosa*.

For peer review only

Item	Number	Recommendation	Assessment of microbiology data reported in this manuscript
		to-date.html) and the List of Prokaryotic Names with Standing in Nomenclature (http://www.bacterio.net/). Organisms considered contaminants should be listed, if appropriate (e.g. coagulase negative staphylococci or *Corynebacterium* spp..	
Setting	5*	Geographical setting: Describe the geographical distribution of specimens/patients from which isolates were obtained, at least to a country level, but preferably to a sub-national level or a geoposition.	Isolates reported were from patients in the catchment area of each hospital (in Hai Phong and Dong Thap province, Viet Nam).
	6*	Clinical setting: Describe the type and level of the healthcare facilities (e.g. primary, secondary, tertiary) from which specimens were obtained. If stating a microbiology laboratory, the centres served by the laboratory should be specified.	Microbiology data were from the routine microbiology laboratory at each of the two provincial hospitals, which are general hospitals in each province in the Viet Nam public healthcare system (consisting of national, provincial and district level hospitals).
Laboratory work	7	Specimen processing: If applicable, describe specimen collection and handling, processing and sub-culture methods for all types of specimen included. For example, if reporting AST results for blood culture and cerebrospinal fluid culture isolates, the processing of these specimens by the laboratory should be briefly explained, including how specimens are sub-cultured, the media used, incubation conditions and duration. A summary of specimen processing steps (e.g. pre-processing steps, nucleic acid extraction method (if applicable), amplification platform, contamination avoidance strategy) should be provided for molecular-only workflows (e.g. to detect *Mycobacterium tuberculosis* and rifampicin resistance using the Cepheid Xpert MTB/RIF system).	Specimen collection and handling, processing and sub-culture methods followed hospital Standard Operating Procedures developed based on the guidelines issued by the Ministry of Health (MoH) in 2017, the subsequent trainings from MoH and OUCRU. Blood culture is performed with biphasic blood culture bottles (Nam Khoa Biotek, Vietnam) in Hospital 1 and with BacT-Alert automate in Hospital 2 (Biomerieux, France). Cultures are performed on homemade media (Oxoid dehydrated culture media, UK) or ready-to-use media (DEKA, Vietnam) for blood agar, chocolate agar, and Mueller-Hinton.
	8*	Target organism identification: Details of identification methodology should be reported briefly. Where identification databases were used (e.g. bioMerieux API/bioMerieux VITEK-MS/Bruker Biotyper), the version should be specified.<br>In general, all pathogens should be identified to species level. In the case of *Salmonella* species, organisms should be identified to at least the *S. Typhi*, *S. Paratyphi*, or non-typhoidal Salmonella (NTS) level. Strain subtyping methods should be reported according to STROME-ID.	Identification was based on the conventional biochemical methods in both hospitals: API galleries (Biomerieux, France) and with manual phenotypic tests in Hospital 1, and with Vitek II (Biomerieux, France; identification database version 9.02) in Hospital 2.

For peer review only

Item	Number	Recommendation	Assessment of microbiology data reported in this manuscript
	9*	Antimicrobial susceptibility testing: Describe the antimicrobial susceptibility testing methods used, internal quality control processes and their interpretation, with reference to a recognised international standard, e.g. CLSI, EUCAST. Where an international standard was followed, the specific edition(s) of guidelines used should be referenced. Deviations from standard methodology should be described, along with evidence of validation. Handling of any changes to interpretative criteria during the sampling period should be documented. State whether the raw AST data (zone diameters and/or minimum inhibitory concentrations) were re-categorised with updated breakpoints or left as-is.	Antimicrobial susceptibility testing methods: disc diffusion at both hospitals; Automated Vitek was also used by Hospital 2;<br>Routine AST data at the microbiology labs was entered into WHONET 5.6 by hospital technicians. Extracted files were converted to WHONET format using BacLink, a free tool included in WHONET. AST results were categorised in S/I/R according to CLSI 2020 guidelines for the present report.
	10	Additional tests performed to identify resistance mechanisms: Describe the testing methods used for adjunctive/confirmatory antimicrobial susceptibility tests, such as enzymatic/molecular assays (e.g. Xpert MTB/RIF, mecA PCR) and inducible resistance assays, with reference to a recognised international standard, where available. Where an international standard was followed, the specific edition of guidelines used should be referenced. Deviations from standard methodology should be described, along with evidence of validation.	Not applicable.
	11*	Antimicrobial resistance definitions: Define resistance for each antimicrobial class (i.e. are isolates in the 'intermediate' category included within 'susceptible' or 'resistant' or analysed as a distinct category). If using the term, define MDR (e.g. ≥ 1 agent in ≥ 3 classes tested). For each organism type, an MDR test panel must be defined, consisting of the minimum panel of individual antimicrobial agents/classes against which an isolate must be tested for that isolate to be considered tested for MDR status. Antimicrobials to which an organism is intrinsically resistant cannot be part of the test panel or contribute to MDR status.	Definitions of resistance to carbapenem and MDR were described in the main methods section of the manuscript based on the criteria proposed by Magiorakos et al [14] as follows:<br>- For *Acinetobacter baumannii* and *Acinetobacter spp.*: MDR is defined as resistant to at least 3 of the following: cephalosporin (ceftriaxone or cefepime), aminoglycosides (amikacin, gentamicin or tobramycin), ciprofloxacin and carbapenem (imipenem or meropenem); carbapenem resistance is defined as resistant to imipenem or meropenem;<br>- For *Escherichia coli, Klebsiella pneumoniae,* and *Klebsiella spp.*: MDR is defined as resistant to at least 3 of the following: carbapenem (ertapenem, imipenem or meropenem), cephalosporin (ceftriaxone or cefepime),

Item	Number	Recommendation	Assessment of microbiology data reported in this manuscript
			aminoglycosides (amikacin, gentamicin or tobramycin) and ciprofloxacin; carbapenem resistance is defined as resistant to ertapenem, imipenem, or meropenem;<br>- For *Pseudomonas aeruginosa*: MDR is defined as resistant to at least 3 of the following 4 agents: imipenem, ceftazidime, ciprofloxacin and tobramycin; carbapenem resistance is resistant to imipenem or meropenem;
Quality assurance	12*	External quality assurance: State whether the microbiology laboratory participates in an external quality control programme and, if so, provide scheme details. Examples include the UK National External Quality Assurance Scheme (www.ukneqasmicro.org.uk) and the American College of Pathologists External Quality Assurance/Proficiency Testing Program (https://www.cap.org/)	Hospital 1: participated in two external quality control programs: UK NEQAS and Ho Chi Minh City quality assurance program<br>Hospital 2: participated in two external quality control programs: UK NEQAS and Hanoi Medical University – Viet Nam
	13	Accreditation: State whether the laboratory is accredited through a national or international body (e.g. the International Standards Organisation, ISO) and specify which assays are covered in the accreditation.	No ISO certification. Hospital 2 has participated in the Strengthening Laboratory Management Toward Accreditation (SLMTA) program.
Bias	14*	Duplicate and sequential isolates: The strategy for accounting for duplicate and sequential isolates from the same patient should be clearly detailed. Duplicate isolates are multiple isolates of the same phenotypic organism (i.e. same species and same resistance profile) from the same patient on the same date cultured either from the same clinical specimen, or from two separate clinical specimens, such as blood and CSF. Sequential isolates are isolates of the same phenotypic organism from the same patient at different dates, such as blood cultures taken on different dates. Various strategies for the handling of duplicate and sequential isolates exist, and the strategy used should be transparent as it will bias pooled resistance results. For example, inclusion of all isolates (the 'all isolate strategy') has been shown to shift pooled resistance proportions toward greater resistance, whilst inclusion of only the first isolate per patient (the 'first isolate strategy') or only the first isolate per	WHONET data were de-duplicated, one isolate representing one patient. Only the first isolate per patient, per pathogen, per reporting period (i.e. for the whole year 2019) at each hospital was included.

For peer review only

Item	Number	Recommendation	Assessment of microbiology data reported in this manuscript
		infection episode (the 'episode-based strategy') will shift pooled results toward susceptibility.	
Results			
	15*	Population: Describe the demographics of the population from which clinical specimens and subsequent isolates have been obtained, disaggregating age and gender data.	Population: patients admitted to each hospital in one calendar year (2019) and stratified by clinical wards. Data on the isolates were described with age and gender information in the manuscript.
	16*	Denominators: Patient and isolate denominators should be used appropriately to ensure clarity regarding the numbers included in each analysis. Of particular importance is the reporting of resistance where first- and second-line AST panels were used (i.e. not all isolates of a particular species were tested against all agents). For drugs where only a subset of isolates were tested, reporting of a percentage without the numbers of isolates tested/resistant may be highly misleading.	Denominators were presented for each percentage described in the manuscript main text. No second-line AST is used in either one of the hospitals.
	17	Site/place of acquisition: AST data from CAI and HAI should be reported and analysed separately.	Not reported from the microbiology data from lab.
	18*	Reporting resistance proportions for single agent and class resistance: Proportions of resistant isolates should be reported as number of isolates susceptible or resistant to a given antimicrobial agent/class out of actual number of isolates tested for susceptibility to that agent/class.	The manuscript focuses on specific types of resistance for each pathogen and reported the proportions of resistant isolates out of the actual number of isolates tested for susceptibility to the specific agents under each type.
	19	Reporting multidrug resistance proportions: If defined, the proportion of MDR isolates should be expressed as the number of MDR isolates out of the number of isolates tested (i.e. the number undergoing the MDR test panel specific to that organism). Single agent/class resistance should be always be reported, regardless of MDR reporting.	As criterion 18. We reported proportions of resistance to single agent or class in Supplementary Table 4.
Discussion			
	20	Discuss any reasons why bias may have been introduced into the reported	We reported the baseline AST data of the study wards in each

For peer review only - http://bmjopen.bmj.com/site/about/guidelines.xhtml

Item	Number	Recommendation	Assessment of microbiology data reported in this manuscript
Limitations		data, due to patient/specimen selection, isolation of organisms, or otherwise. Consider factors which may have either introduced bias into the types of organisms isolated or the antimicrobial susceptibility profiles, e.g. receipt of antimicrobials prior to specimen collection will reduce the yield of certain species and also select for more resistant organisms.	hospitals using the routine data collected for diagnostic reasons. No diagnostic stewardship program existed at baseline in either one of the 2 hospitals. Therefore, it is expected that the clinicians' testing habits have impacted the selection of samples and isolates subsequently tested for antimicrobial susceptibility. Because reimbursement of laboratory test is based on strict clinical criteria, the isolates may not represent the population of pathogens, but this bias remains constant between wards, between hospitals, and with time.

*Core 'must include' items

For peer review only

Supplementary Table 3. Summary of National AMS Stakeholder Meeting in March 2019

**Participating stakeholders**	Medical Services Administration (MSA) Viet Nam<br>Vietnamese Pharmaceutical Association<br>Viet Nam Centers for Disease Control and Prevention<br>Hanoi Medical University, Hanoi<br>Hanoi University of Pharmacy, Hanoi<br>Drugs Administration Viet Nam, MoH<br>MSD Pharmaceuticals<br>National Hospital for Tropical Diseases, Hanoi<br>Hospital for Tropical Diseases, Ho Chi Minh City<br>Viet Nam National Children Hospital, Hanoi<br>Cho Ray Hospital, Ho Chi Minh city<br>Uong Bi Viet Nam – Sweden Hospital, Quang Ninh<br>Can Tho General Hospital<br>Da Nang General Hospital<br>Hue General Hospital<br>Children's Hospital 1, Ho Chi Minh City<br>Hoan My Private Hospital, Viet Nam<br>World Health Organization (WHO) – Viet Nam<br>Oxford University Clinical Research Unit (OUCRU)<br>US Centers for Disease Control and Prevention – Viet Nam<br>Woolcock Viet Nam<br>PATH Viet Nam
**Key presentations and discussions**	MSA/ WHO implementation of antimicrobial stewardship in hospitals: review of current state and hospital survey;<br>AMS implementation experience of Cho Ray Hospital, Hue Central Hospital, National Hospital for Tropical Diseases, and Hoan My Hospital;<br>MSD program on antimicrobial stewardship in Viet Nam: a perspective from the pharmaceutical company;<br>OUCRU qualitative study on AMS implementation in seven hospitals in the AMR surveillance network;<br>Group discussions on the challenges faced in AMS and proposed solutions to improve the implementation;<br>WHO Coordination and Finding the Way Forward.
**Main areas for actions identified**	1. Strengthening a supportive policy environment for AMS: With the National Action Plan to combat AMR and subsequent guidelines, the legal framework has been set up and shows the strong commitment of the government in AMS implementation in Viet Nam. However, national guidelines

For peer review only

	clearly need revisions and updates to fit in the different contexts where AMS work is required, particularly in small and resources-limited healthcare settings.<br>2. Developing AMS training curriculum and core AMS related competencies: Training modules on AMS should be standardized and formalized into the undergraduate and Continuing Professional Development programs and available in a variety of formats including online courses. These should also be available to any healthcare professionals taking on AMS roles in hospital and other settings.<br>3. Establishing coordination and collaboration in AMS activities nationally and regionally: Coordination of activities across the country and in each region is crucial to achieve a synergy of efforts and minimize duplications and wastes of resources. Smaller hospitals can learn from and utilize resources available from larger hospitals in the same region. Hospitals with experience in implementing AMS programs and those having been identified as centers of excellence can share experience with and support other hospitals in this process.

For peer review only

Supplementary Table 4. Baseline proportions of resistance for common pathogens to each agent/class under evaluation

**Resistant / Tested (%)**	***E. coli***		***K. pneumoniae**** **or *Klebsiella spp.*****		***A. baumannii**** **or *Acinetobacter spp.*****		***P. aeruginosa***		***S. aureus***	
	All specimens	Blood	All specimens	Blood	All specimens	Blood	All specimens	Blood	All specimens	Blood
**Hospital 1**										
Carbapenem	45/540 (8.3)	4/32 (12.5)	44/167 (26.3)	2/10 (20.0)	66/103 (64.1)	1/4 (25.0)	36/86 (41.9)	0/2 (0.0)	-	-
Cephalosporin	360/540 (66.7)	27/32 (84.4)	95/167 (56.9)	6/10 (60.0)	87/103 (84.5)	1/4 (25.0)	34/86 (39.5)	0/2 (0.0)	-	-
Aminoglycoside	245/540 (45.4)	21/32 (65.6)	71/167 (42.5)	5/10 (50.0)	86/103 (83.5)	2/4 (50.0)	47/86 (54.7)	1/2 (50.0)	115/203 (56.7)	25/40 (62.5)
Ciprofloxacin	420/539 (77.9)	29/32 (90.6)	111/167 (66.5)	5/10 (50.0)	85/102 (83.3)	1/4 (25.0)	45/86 (52.3)	0/2 (0.0)	94/210 (44.8)	26/42 (61.9)
Ceftazidime	288/540 (53.3)	24/32 (75.0)	98/167 (58.7)	4/10 (40.0)	79/103 (76.7)	1/4 (25.0)	35/85 (41.2)	0/2 (0.0)	-	-
Cefoxitin	163/540 (30.2)	12/32 (37.5)	72/167 (43.1)	3/10 (30.0)	-	-	-	-	166/210 (79.0)	-
MDR	184/539 (34.1)	21/32 (65.6)	66/167 (39.5)	4/10 (40.0)	62/102 (60.8)	0/4 (0.0)	35/86 (40.7)	0/2 (0.0)		
**Hospital 2**										
Carbapenem	22/297 (7.4)	3/96 (3.1)	151/289 (52.2)	28/62 (45.2)	260/325 (80.0)	11/19 (57.9)	89/254 (35.0)	3/14 (21.4)	-	-
Cephalosporin	69/273 (25.3)	17/83 (20.5)	141/256 (55.1)	28/61 (45.9)	226/269 (84.0)	11/17 (64.7)	41/175 (23.4)	2/11 (18.2)	-	-
Aminoglycoside	137/297 (46.1)	39/96 (40.6)	149/288 (51.7)	32/62 (51.6)	229/326 (70.2)	11/19 (57.9)	137/254 (53.9)	6/14 (42.9)	79/178 (44.4)	14/32 (46.9)
Ciprofloxacin	197/275 (71.6)	58/88 (65.9)	188/253 (74.3)	36/60 (60.0)	252/306 (82.3)	11/19 (57.9)	124/207 (59.9)	6/12 (50.0)	71/170 (41.8)	11/31 (35.5)
Ceftazidime	121/295 (41.1)	31/95 (32.6)	182/288 (63.2)	35/62 (56.5)	273/323 (84.5)	12/19 (63.2)	64/253 (25.3)	2/14 (14.3)	-	-
Cefoxitin	17/55 (30.9)	8/23 (34.8)	29/60 (48.3)	3/5 (60.0)	-	-	-	-	79/103 (76.7)	8/17 (47.1)
MDR	55/267 (20.6)	13/83 (15.7)	127/249 (51.0)	26/59 (44.1)	166/265 (62.6)	8/17 (47.1)	66/272 (24.3)	2/25 (8.0)	-	-

* in Hospital 2; ** in Hospital 1. Cephalosporin: ceftriaxone or cefepime; Aminoglycoside: amikacin, gentamicin, or tobramycin; Carbapenem: imipenem or meropenem (for *A. baumannii*, *Acinetobacter spp.*, *P. aeruginosa*); Carbapenem: ertapenem, imipenem, or meropenem (for *E. coli, K. pneumoniae, Klebsiella spp.*).
MDR for *A. baumannii* and *Acinetobacter spp.*: resistant to at least 3 of the following: cephalosporin (ceftriaxone or cefepime), aminoglycosides (amikacin, gentamicin or tobramycin), ciprofloxacin and carbapenem (imipenem or meropenem). MDR for *E. coli, K. pneumoniae, and Klebsiella spp.*: resistant to at least 3 of the following: carbapenem (ertapenem, imipenem or meropenem), cephalosporin (ceftriaxone or cefepime), aminoglycosides (amikacin, gentamicin or tobramycin) and ciprofloxacin; MDR for *P. aeruginosa*: resistant to at least 3 of the following 4 agents: imipenem, ceftazidime, ciprofloxacin and tobramycin.

For peer review only

Standards for Reporting Implementation Studies: the StaRI checklist for completion

Notes: A key concept of the StaRI standards is the dual strands of describing, on the one hand, the implementation strategy and, on the other, the clinical, healthcare, or public health intervention that is being implemented. These strands are represented as two columns in the checklist.

The primary focus of implementation science is the implementation strategy (column 1) and the expectation is that this will always be completed.

The evidence about the impact of the intervention on the targeted population should always be considered (column 2) and either health outcomes reported or robust evidence cited to support a known beneficial effect of the intervention on the health of individuals or populations.

The StaRI standardsrefers to the broad range of study designs employed in implementation science. Authors should refer to other reporting standards for advice on reporting specific methodological features. Conversely, whilst all items are worthy of consideration, not all items will be applicable to, or feasible within every study.

Checklist item		Reported on page #	Implementation Strategy	Reported on page #	Intervention
			"Implementation strategy" refers to how the intervention was implemented		"Intervention" refers to the healthcare or public health intervention that is being implemented.
**Title and abstract**					
Title	**1**	1	Identification as an implementation study, and description of the methodology in the title and/or keywords		
Abstract	**2**	2	Identification as an implementation study, including a description of the implementation strategy to be tested, the evidence-based intervention being implemented, and defining the key implementation and health outcomes.		
**Introduction**					
Introduction	**3**	4-5	Description of the problem, challenge or deficiency in healthcare or public health that the intervention being implemented aims to address.		
Rationale	**4**	6-7	The scientific background and rationale for the implementation strategy (including any underpinning theory/framework/model, how it is expected to achieve its effects and any pilot work).	4	The scientific background and rationale for the intervention being implemented (including evidence about its effectiveness and how it is expected to achieve its effects).
Aims and objectives	**5**	5	The aims of the study, differentiating between implementation objectives and any intervention objectives.		
**Methods: description**					
Design	**6**	5-8	The design and key features of the evaluation, (cross referencing to any appropriate methodology reporting standards) and any changes to study protocol, with reasons		

Context	**7**	5	The context in which the intervention was implemented. (Consider social, economic, policy, healthcare, organisational barriers and facilitators that might influence implementation elsewhere).		
Targeted 'sites'	**8**	5-6	The characteristics of the targeted 'site(s)' (e.g locations/personnel/resources etc.) for implementation and any eligibility criteria.	6	The population targeted by the intervention and any eligibility criteria.
Description	**9**	5-8	A description of the implementation strategy	10-11	A description of the intervention
Sub-groups	**10**	6	Any sub-groups recruited for additional research tasks, and/or nested studies are described		
**Methods: evaluation**					
Outcomes	**11**	7	Defined pre-specified primary and other outcome(s) of the implementation strategy, and how they were assessed. Document any pre-determined targets	7	Defined pre-specified primary and other outcome(s) of the intervention (if assessed), and how they were assessed. Document any pre-determined targets
Process evaluation	**12**	7-8	Process evaluation objectives and outcomes related to the mechanism by which the strategy is expected to work		
Economic evaluation	**13**	*	Methods for resource use, costs, economic outcomes and analysis for the implementation strategy	*	Methods for resource use, costs, economic outcomes and analysis for the intervention
Sample size	**14**	6**	Rationale for sample sizes (including sample size calculations, budgetary constraints, practical considerations, data saturation, as appropriate)		
Analysis	**15**	8-9	Methods of analysis (with reasons for that choice)		
Sub-group analyses	**16**	8-9	Any a priori sub-group analyses (e.g. between different sites in a multicentre study, different clinical or demographic populations), and sub-groups recruited to specific nested research tasks		

For peer review only

For peer review only - http://bmjopen.bmj.com/site/about/guidelines.xhtml

**Results**					
Characteristics	**17**	11-14	Proportion recruited and characteristics of the recipient population for the implementation strategy	NA	Proportion recruited and characteristics (if appropriate) of the recipient population for the intervention
Outcomes	**18**	12-14#	Primary and other outcome(s) of the implementation strategy	NA##	Primary and other outcome(s) of the Intervention (if assessed)
Process outcomes	**19**	10-12	Process data related to the implementation strategy mapped to the mechanism by which the strategy is expected to work		
Economic evaluation	**20**	*	Resource use, costs, economic outcomes and analysis for the implementation strategy	*	Resource use, costs, economic outcomes and analysis for the intervention
Sub-group analyses	**21**	NA##	Representativeness and outcomes of subgroups including those recruited to specific research tasks		
Fidelity/ adaptation	**22**	10-12	Fidelity to implementation strategy as planned and adaptation to suit context and preferences	NA	Fidelity to delivering the core components of intervention (where measured)
Contextual changes	**23**	10-12	Contextual changes (if any) which may have affected outcomes		
Harms	**24**	NA##	All important harms or unintended effects in each group		
**Discussion**					
Structured discussion	**25**	14-17	Summary of findings, strengths and limitations, comparisons with other studies, conclusions and implications		
Implications	**26**	18	Discussion of policy, practice and/or research implications of the implementation strategy (specifically including scalability)	18	Discussion of policy, practice and/or research implications of the intervention (specifically including sustainability)
**General**					
Statements	**27**	9-10	Include statement(s) on regulatory approvals (including, as appropriate, ethical approval, confidential use of routine data, governance approval), trial/study registration (availability of protocol), funding and conflicts of interest		

* We mentioned about recording the economic indicators, however methods and analysis will be done and reported separately; the costing methodology employed will be the time-driven activity based costing approach. ** We selected 4 intervention wards and 4 control wards based on the funding availability. #We presented in this manuscript the outcomes of our implementation strategy that has

For peer review only - http://bmjopen.bmj.com/site/about/guidelines.xhtml

a description of the process in setting up the program at each hospital and baseline data for outcome indicators of antibiotic use and resistance. ##These will be described at the end of the intervention period in the next report.

For peer review only
